# Elucidating the Role of Energy- and Electron-Transfer Processes and the Route to Highly Reduced Products in the Dual Ir^III^/Fe^III^ Photocatalyzed CO_2_ Reduction Reaction

**DOI:** 10.1021/jacs.6c11427

**Published:** 2026-09-16

**Authors:** Stephen P. DiLuzio, Keon Ha Hwang, Kaitlin M. Luedecke, Erica Sutcliffe, Ryan G. Hadt

**Affiliations:** Division of Chemistry and Chemical Engineering, Arthur Amos Noyes Laboratory of Chemical Physics, 6469 California Institute of Technology, Pasadena, California 91125, United States

## Abstract

Mechanistic elucidation of a photochemically driven CO_2_ reduction reaction (CO_2_RR) using an Ir^III^ photosensitizer and an iron metalloporphyrin reduction catalyst, which has been proposed to transform CO_2_ into CH_4_, has been conducted using various steady-state and time-resolved absorption and emission spectroscopic methods, cyclic voltammetry, reaction kinetics, quantum yield measurements, spectroelectrochemistry, and isotope-labeling experiments. A dynamic reactivity landscape was subsequently uncovered where the key photochemical mechanistic steps are altered as a function of reaction progress from an initial photoinduced single electron transfer to an energy transfer mechanism, which is supported by the *in situ* generation of a ferrous-carbonyl intermediate that was subjected to Stern–Volmer analysis and transient absorption spectroscopy to determine the overall photochemical mechanism using a select Ir^III^ photosensitizer. This alteration of the photochemical mechanistic steps stymies further photoinduced reduction of the ferrous-carbonyl intermediate, which is a proposed on-cycle step toward the photoevolution of CH_4_. Instead, cyclic voltammetry experiments implicated a plausible CH_4_ evolution mechanism involving degradation of the porphyrin structure through attack of a second equivalent of the reduced metalloporphyrin catalyst. This mechanistic picture is consistent for the observed formation of ethane under steady-state irradiation conditions, which also does not arise from the CO_2_RR but instead represents a degradation product of the sacrificial electron donor, triethylamine, following single electron oxidation.

## Graphical abstract

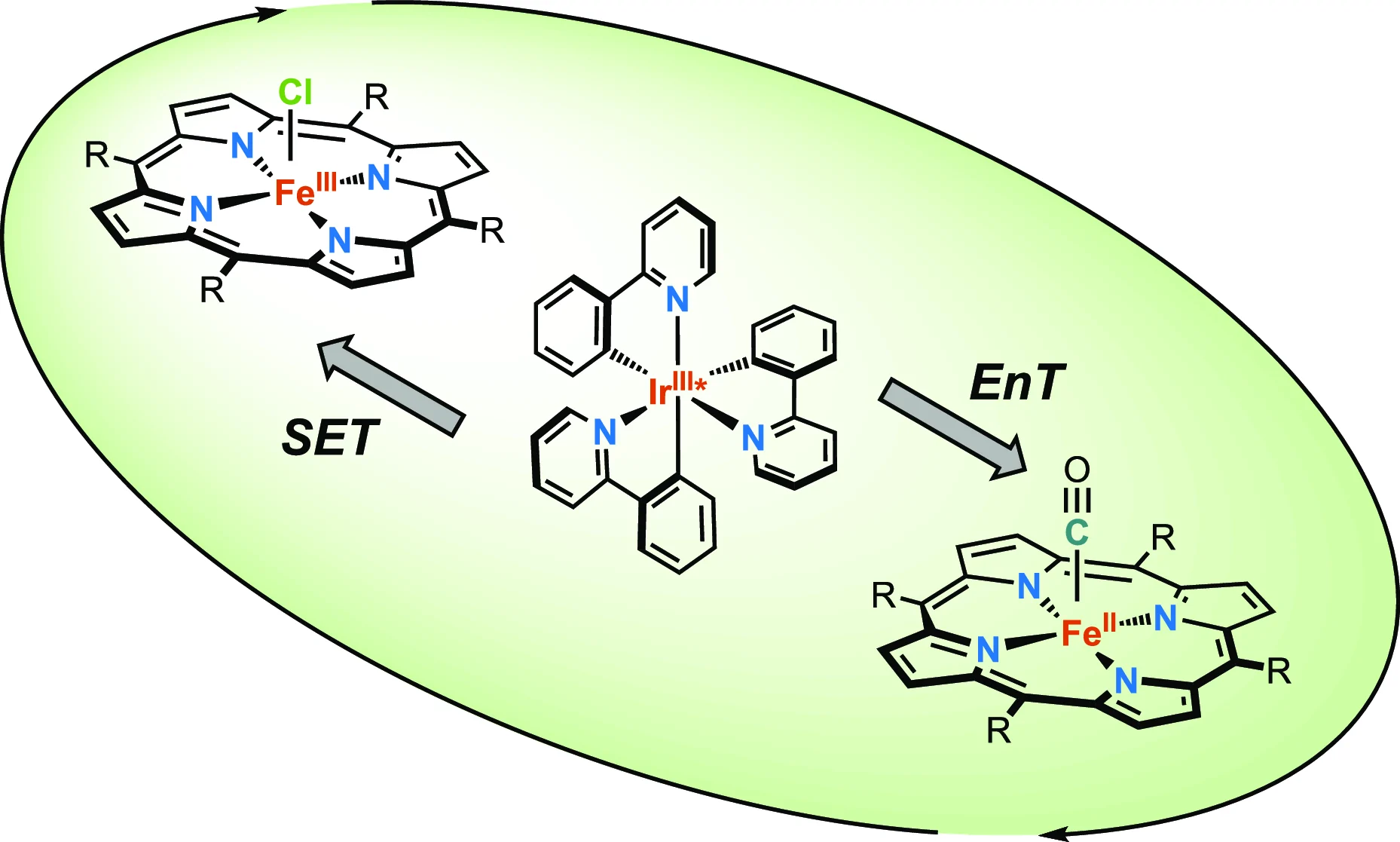

## Introduction

The homogeneous photo- and electrochemical CO_2_ reduction reaction (CO_2_RR) presents an opportunity to mitigate the deleterious effects of increased CO_2_ concentration in Earth’s atmosphere through the reduction of CO_2_ into economically viable products or energy dense fuels.^1–15^ While CO_2_ reduction into 2 H^+^/2 e^–^ reduced products such as CO are well documented,^16^ further reduction into 6 H^+^/6 e^–^ or 8 H^+^/8 e^–^ reduced C_1_ products such as CH_3_OH or CH_4_ transforms CO_2_ into combustible fuels that can be translated into the energy sector.^1,17–22^ Mechanistic pathways for the generation of these products often invoke reduction of on-cycle metal–carbonyl intermediates in both the photo/electrochemical CO_2_RR.^17^ Because these intermediates are generated *in situ*, their mechanistic analysis is challenging.

Metalloporphyins are commonly adopted CO_2_RR catalysts that, in select cases, demonstrate the ability to reduce CO_2_ into desirable highly reduced products (e.g., CH_4_, CH_3_OH).^23–26^ Fe^III^-5,10,15,20-Tetrakis­(trimethylammoniophenyl)­porphyrin tetrachloride ([(*p*–TMA)­Fe^III^–Cl]^4+^, Figure 1A) represents a metalloporphyrin complex that functions both as an electrocatalyst for the CO_2_RR^27,28^ and a light-harvesting species for the photochemical reduction of CO_2_ to CO.^29,30^ In the presence of a photosensitizer (PS) with a highly reducing excited state, such as fac-[Ir­(ppy)_3_] (**Ir1**, *E*_1/2_^IV/^* = −1.73 V vs S.C.E., ppy: 2-phenylpyridine), the Robert group has demonstrated the ability to further transform CO_2_ into the 8 H^+^/8 e^–^ reduced CH_4_ product with isotopically labeled ^13^CO_2_. Systems using triethylamine (TEA) as a sacrificial electron donor (SED) and trifluoroethanol (TFE) as a proton source produced CH_4_ with a quantum yield (QY, Φ) of 0.18%–0.47% (Figure 1A, Scheme S1).^31–33^ The photoevolution of CH_4_ was observed with inorganic or organic PSs possessing a potent excited-state reduction potential. Given the stringent requirement of a highly reducing excited state (PS*), oxidative quenching of PS* has been proposed to both initiate and drive the photochemical CO_2_RR; however, related reductive quenching pathways also produce CH_4_.^34^ Recent work has demonstrated electrochemical evolution of CH_4_ that does not arise as a CO_2_ reduction product, but rather is proposed to arise from hydrolysis induced decomposition of the anilinium-methyl groups in the porphyrin structure.^16^

**Figure 1:**
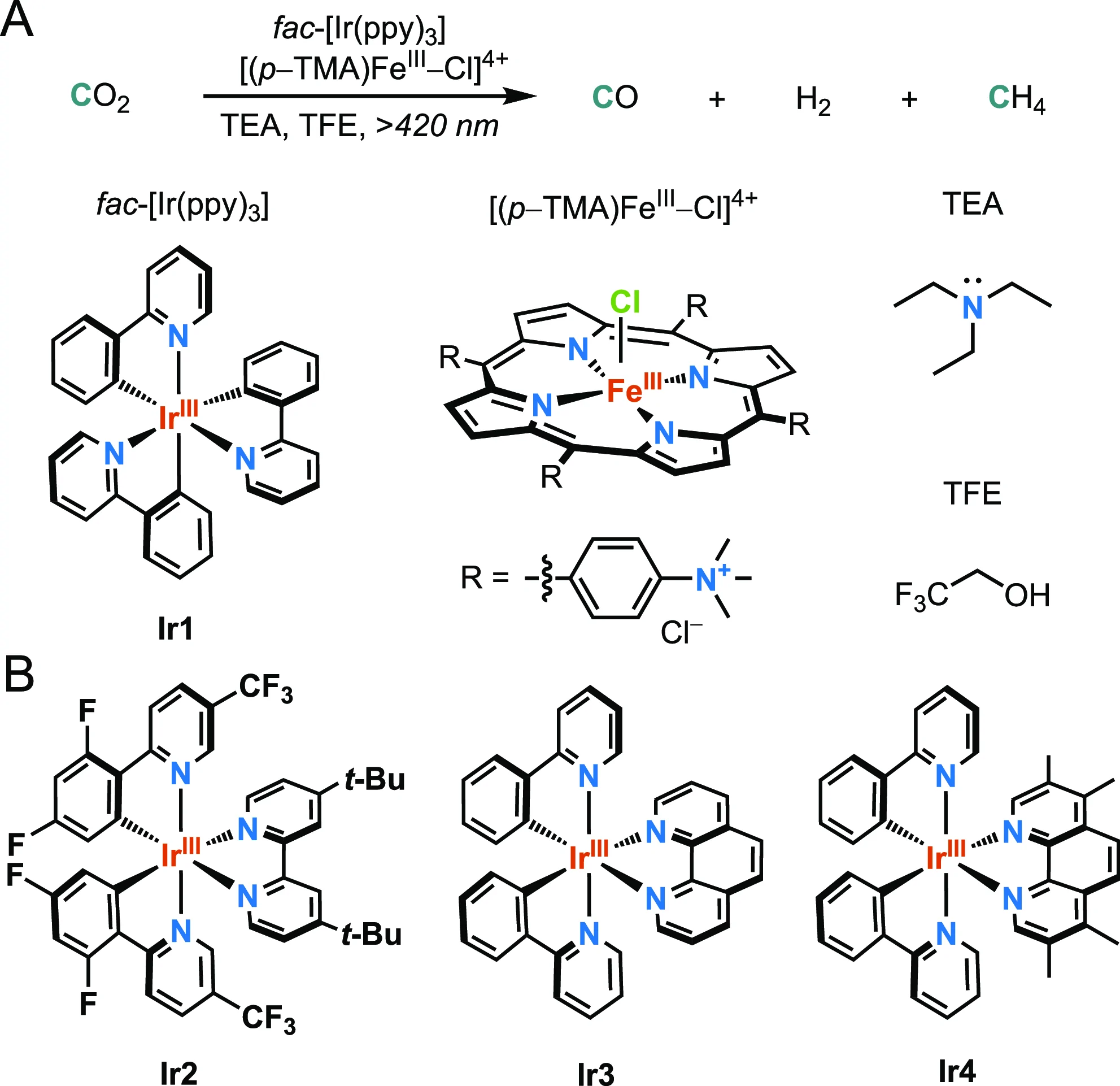
Reactivity and chemical structures considered in this work. (A) Reported PS and CO_2_RR capable of transforming CO_2_ into CO and CH_4_. (B) Structures of heteroleptic Ir^III^ complexes studied here.

Photoredox catalysis modulates the CO_2_RR catalyst’s oxidation state through bimolecular single electron transfer (SET) reactions. In addition to photoinduced SET, photochemical reaction platforms also allow for alternative energy transfer (EnT) reaction steps to proceed where excited-state energy is transferred from PS* to a suitable acceptor species instead of the formation of freely diffusing redox products.^35^ Significant spectral overlap between the photoluminescent spectral profile of PS* and the absorption profile of the acceptor represents a prerequisite for EnT processes such as Förster Resonance Energy Transfer (FRET). [(*p*–TMA)­Fe^III^–Cl]^4+^ represents a unique quencher in this capacity with readily accessible Fe^III/II^ reduction potentials (i.e., ∼−0.3 V vs S.C.E) concurrent with significant visible broadband absorption, making both SET and EnT possible. The accessible excited-state quenching processes likely depend both on the excited-state reduction potentials of the PS that control the driving force for SET, as well as the spectral overlap of the PS* photoluminescence spectra and the absorption spectrum of [(*p*–TMA)­Fe^III^–Cl]^4+^. Crucially, as the oxidation state of the [(*p*–TMA)­Fe^III^–Cl]^4+^ precatalyst continues to change upon SET reduction, so will the thermodynamic driving force for SET and the UV–vis absorption spectra of the CO_2_RR catalyst, setting the stage for highly dynamic photochemical processes that are altered as a function of reaction progress.

To investigate this phenomena, time-resolved photoluminescence and single wavelength transient absorption (TA) spectroscopy paired with spectroelectrochemistry have been conducted on a variety of Ir^III^ PSs (Figure 1B, **Ir1–Ir4**) that together indicated a change in the excited-state quenching pathway from an initial photoinduced SET through an oxidative quenching mechanism between [(*p*–TMA)­Fe^III^–Cl]^4+^ and Ir1* to that of an EnT mechanism between Ir1* and the ferrous-carbonyl intermediate, [(*p*–TMA)­Fe^II^–CO]^4+^, which is on-cycle in the photochemical CO_2_RR. These spectroscopic methods are correlated to reaction kinetics under steady-state irradiation conditions, where alternative reaction dynamics yield a nearly two-order of magnitude increase in reaction QYs for CO photoevolution. CH_4_ and C_2_H_6_ are both observed as photoproducts under optimized conditions; however, isotopically labeled ^13^CO_2_ confirms that these products do not form from the CO_2_RR. Instead, cyclic voltammetry implicates formation of these products from a degradation mechanism between the reduced metalloporphyrin, [(*p*–TMA^••^)­Fe^II^]^2+^, with the methyl-anilinium groups for CH_4_ and the protonated TEA (TEA-H) for C_2_H_6_. These results altogether highlight the importance of considering the explicit mechanistic steps between PS* and the various speciation/oxidation states of the CO_2_RR catalyst throughout the reaction cycle, as well as the source of highly reduced product formation using commonly adopted catalysts and sacrificial electron donors.

## Results

### Photocatalytic Product Speciation

We began our investigation with separate optimization studies using homoleptic **Ir1** (Figures S1–S4 and Table S1), which was originally used since it possesses a highly reducing excited state, and the highly photo-oxidizing heteroleptic [Ir­(dF­(CF_3_)­ppy)_2_(dtbbpy)]^+^PF_6_^–^ (**Ir2**, *E*_1/2_*^/•–^ = +1.24 V vs S.C.E., Figures S5–S9 and Table S2).^36,37^ TEA was used as the sacrificial electron donor, and TFE acted as the proton source in accordance with original reports.^31,32^ The optimized **Ir1** system produces CO over a three-day period, demonstrating exceptional photostability, which was previously experimentally observed (Φ_CO_ = 0.135%, Figure 2A).^31^ CH_4_ photoevolution is also observed. However, the Turnover Numbers (TONs) do not exceed one, rendering this process not catalytic (Figure 2B). Alternatively, the system using **Ir2** represents a much more highly active system (Φ_CO_ = 14.1%), evolving CO and C_2_H_6_. However, photoevolution of these products concludes after 2 h (Figure 2A,B). GC–MS experiments with ^13^CO_2_ indicate that neither CH_4_ using **Ir1** nor C_2_H_6_ using **Ir2** include carbon from CO_2_ (Figure 2C) despite ^13^CO production (Figure S10). The hydrogen evolution reaction (HER) also proceeds in both systems (Tables S1 and S2).

**Figure 2:**
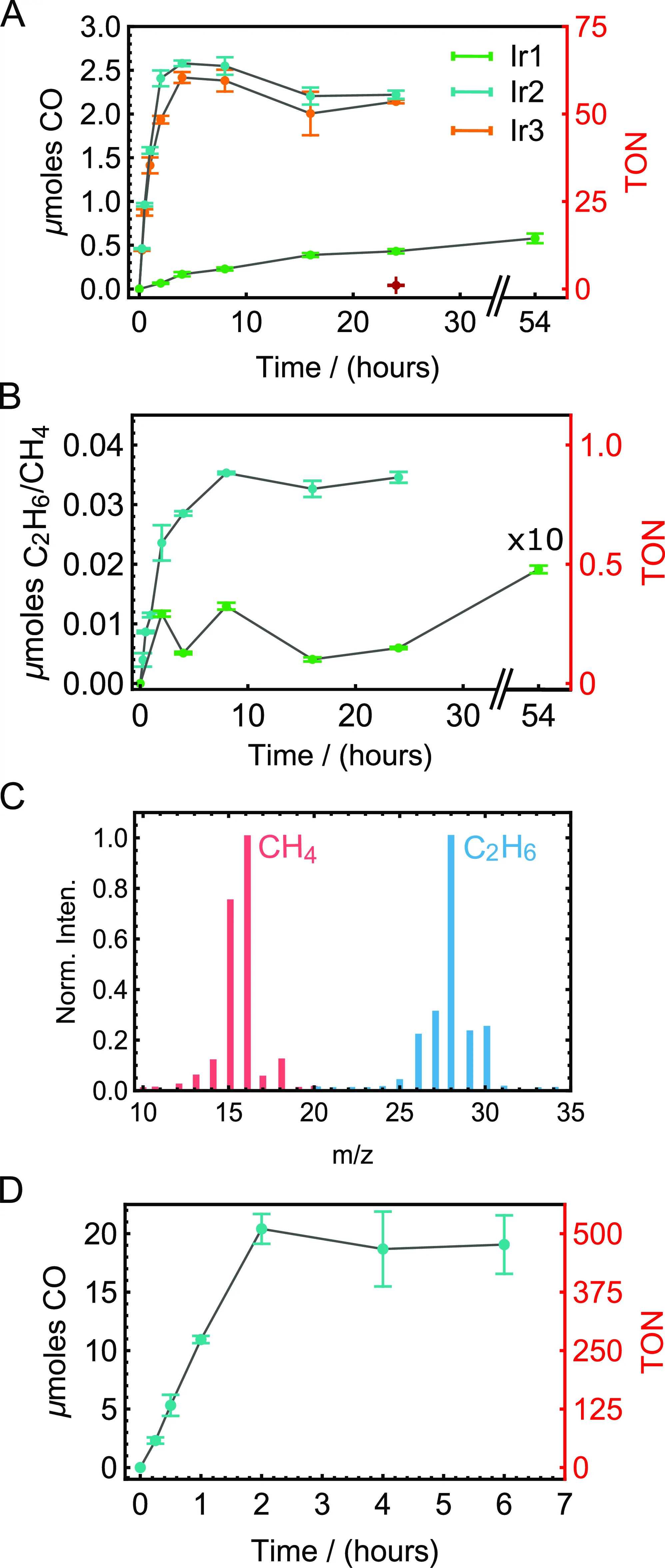
Optimized photochemical reaction conditions. (A) Photoevolution of CO under optimized conditions for **Ir1** (green), **Ir2** (teal), and **Ir3** (orange). The red point represents the no PS control. (B) Photoevolution of CH_4_ (green, ×10 magnification) and C_2_H_6_ (blue) under the optimized conditions using **Ir1** and **Ir2** as the PS, respectively. (C) Independently normalized GC–MS results using ^13^CO_2_, indicating ^12^CH_4_ as the major source of methane detected when using **Ir1** and detected ^12^C_2_H_6_ under optimized conditions for **Ir2**. (D) Photoevolution of CO using **Ir2** as a PS and 2TBA^+^ Ox^2–^ as the sacrificial electron donor.

Since CH_4_ and C_2_H_6_ are both desirable highly reduced products from the CO_2_RR, we sought to identify the source of these products since they do not arise from CO_2_ under the conditions reported here. With respect to the **Ir2** systems, which unexpectedly evolve C_2_H_6_ (Figure 2B), screening of the sacrificial electron donors (Table 1, structures shown in Table S3) shows catalytic activity for CO evolution, yet no observation of C_2_H_6_ (triethanolamine, however, does yield C_2_H_4_), rendering C_2_H_6_ photoevolution explicitly linked to the use of TEA.

**Table 1:** μmoles of CO, C_2_H_4_, and C_2_H_6_ Evolved with Various Sacrificial Electron Donors under the Optimized Conditions for **Ir2**

SED	μmoles CO	TON_Fe_	C_2_H_4_	C_2_H_6_
TEA	2.11	55	N.D	0.032
TEOA	1.02	25.6	0.019	N.D
DMA	0.024	0.6	N.D	N.D
BIH	1.78	44.3	N.D	N.D
DIPEA	1.05	26.3	N.D	N.D
DABCO	0.004	0.1	N.D	N.D
2TBA^+^ Ox^2–^	17.5	485.3	N.D	N.D
TBA^+^ Br^–^	0.008	0.2	N.D	N.D

Reversible electron donors such as DMA, DABCO, and Br^–^ yield systems that do not demonstrate catalytic activity for CO photoevolution. Intriguingly, when tetrabutylammonium oxalate (2TBA^+^ Ox^2–^) acts as the electron donor, the TON of CO with respect to Fe approaches ∼500 (Figure 2D). As this system demonstrates exceptional catalytic activity, yet no formation of CO_2_RR products beyond CO, the need for CO to act as an intermediate may be unnecessary. The oxalate anion acts as the electron donor that reductively quenches Ir2* photoluminescence, which then forms Ox^+^ and subsequently decomposes into CO_2_ and the highly reducing CO_2_ radical anion (*E*_1/2_ ∼ −2.1 V vs S.C.E., CO_2_^•–^).^38^ In this capacity, 2TBA^+^ Ox^2–^ acts as an irreversible two-electron donor under catalytic conditions. The tertiary amines that yield catalytic systems for CO possess known deleterious reactivity pathways that often hydrogenate aromatic systems, which likely gives rise to the lower TONs for TEA, TEOA, DIPEA, and BIH.^39,40^ In total, TEA likely acts as the source of C_2_H_6_, although the exact mechanism is not yet clear. GC–MS experiments using TEA-*d*_15_ does increase the measured *m*/*z* of C_2_H_6_, further supporting TEA degradation as the source of C_2_H_6_ (Figure S11). No C_2_H_6_ is observed in the absence of [(*p*–TMA)­Fe^III^–Cl]^4+^ (Table S2), implicating its role in C_2_H_6_ production as well.

### Time-Resolved Spectroscopy

To help elucidate these different reactivity pathways, we first wanted to uncover the source of the significant disparity between the QYs of CO production when using **Ir1** (Φ_CO_ = 0.135%) and **Ir2** (Φ_CO_ = 14.1%), along with the resultant kinetic profiles. Steady-state and time-resolved photoluminescence and single-wavelength TA spectroscopy were conducted to probe both the magnitude and mechanism of the excited-state quenching processes between the excited PSs and various catalysts and electron donors in solution. No interaction between Ir1* and TEA is observed, whereas TEA quenches Ir2* photoluminescence with a bimolecular rate constant of 6.04 × 10^9^ M^–1^ s^–1^ (Figure S12). Correspondingly, it is reasonable to suggest that the photochemical systems where the PS is solely interacting with the metalloporphyrin CO_2_RR catalyst proceed with lower reaction QYs compared to systems where reductive quenching pathways are available to drive the catalytic cycle. Given the broadband absorption of [(*p*–TMA)­Fe^III^–Cl]^4+^ across the visible region, which will attenuate the measured steady-state emission intensity through inner-filter effects (see Section S8 of the Supporting Information, Figure 3A),^41^ we turned to nanosecond time-resolved emission to unambiguously measure potential interactions between [(*p*–TMA)­Fe^III^–Cl]^4+^ and Ir1*–Ir4*. All four PSs were investigated in these experiments to elucidate what photochemical quenching pathways could be available when using [(*p*–TMA)­Fe^III^–Cl]^4+^ as a quencher since these PSs have differing excited-state triplet energies and reduction potentials. Stern–Volmer analysis using time-resolved emission demonstrates the ability of [(*p*–TMA)­Fe^III^–Cl]^4+^ to quench all four PSs emission, where *k*_q_ = 8.07 × 10^9^ M^–1^ s^–1^ for **Ir1** and *k*_*q*_ = 1.58 × 10^9^ M^–1^ s^–1^, 2.01 × 10^9^ M^–1^ s^–1^, and 1.38 × 10^9^ M^–1^ s^–1^ for **Ir2**–**Ir4**, respectively (Figures 3B, S13–S16 and Table 2).

**Figure 3:**
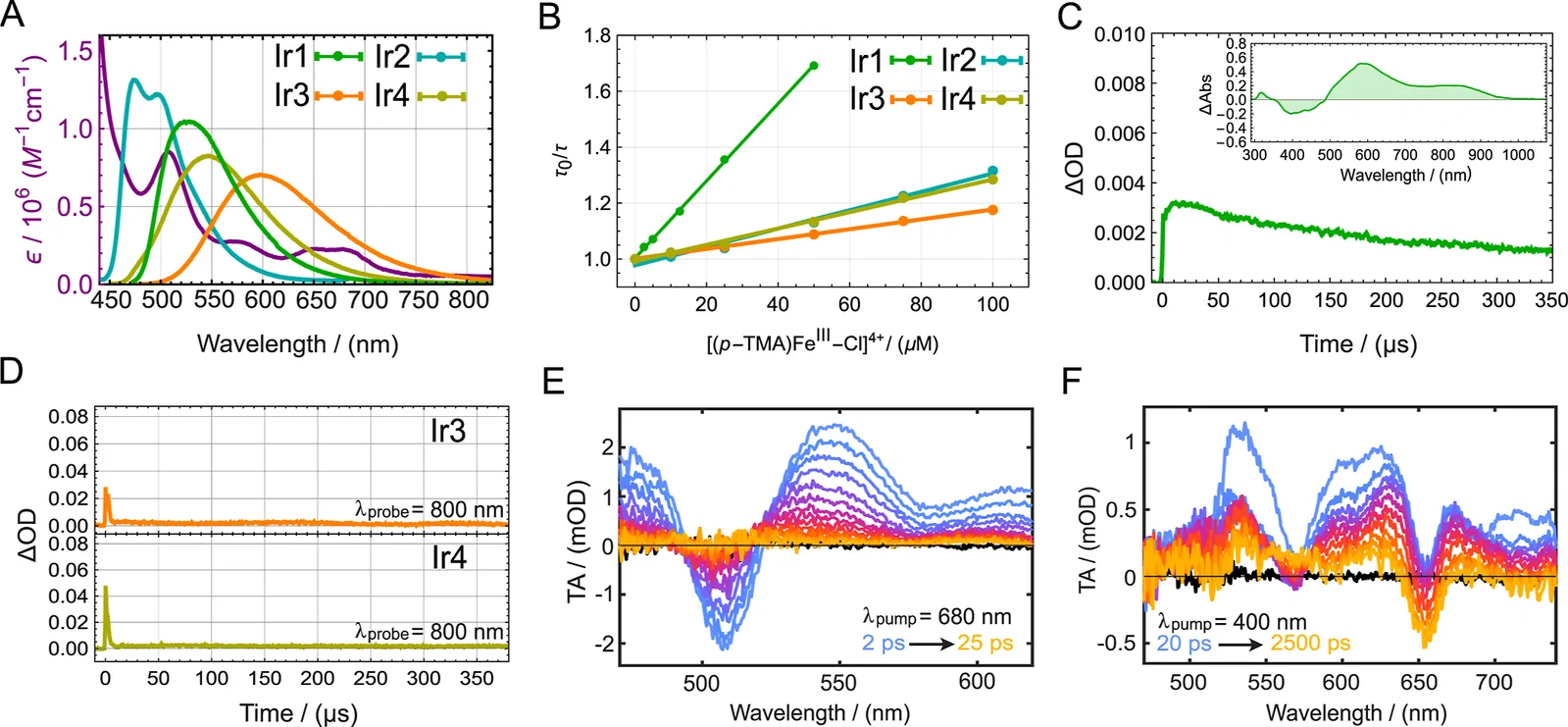
Spectroscopic characterization of photochemical mechanistic steps using [(*p*–TMA)­Fe^III^–Cl]^4+^. (A) UV–vis absorption spectra of [(*p*–TMA)­Fe^III^–Cl]^4+^ (purple) and photoluminescent spectra of **Ir1** (green), **Ir2** (teal), **Ir3** (orange), and **Ir4** (yellow). (B) Stern–Volmer analysis obtained using time-resolved emission data between Ir1* (green), Ir2* (teal), Ir3* (orange), and Ir4* (yellow) and [(*p*–TMA)­Fe^III^–Cl]^4+^. (C) Single-wavelength nanosecond TA of [(*p*–TMA)­Fe^III^–Cl]^4+^ and **Ir1** following a 450 nm pump excitation. λ^probe^: 800 nm, [(*p*–TMA)­Fe^III^–Cl]^4+^: 50 μM. Inset: spectroelectrochemical spectra of **Ir1** ^**+**^. (D) Single-wavelength nanosecond TA of [(*p*–TMA)­Fe^III^–Cl]^4+^ and **Ir3** (orange) and **Ir4** (yellow) following a 450 nm pump. λ^probe^: 800 nm, [(*p*–TMA)­Fe^III^–Cl]^4+^: 50 μM. (E). Longer-lived component in the femtosecond TA of [(*p*–TMA)­Fe^III^–Cl]^4+^ following 680 nm pump excitation, [(*p*–TMA)­Fe^III^–Cl]^4+^: 50 μM. (F) Longer-lived component in the femtosecond TA of [(*p*–TMA)­Fe^III^–Cl]^4+^ following 400 nm pump excitation demonstrating formation of a long-lived photoproduct following the higher energy pump excitation wavelength.

**Table 2:** Bimolecular Quenching Rate Constants, Overlap Integrals, and FRET Term Product for **Ir1**–**Ir4** with [(*p*–TMA)­Fe^III^–Cl]^4+^ and [(*p*–TMA)­Fe^II^–CO]^4+^

PS	*k*_q_ × 10^9^ M^–1^s^–1^	*J*_Overlap_ (cm^3^/M) × 10^–14^	*k*_p_ × *J*_Overlap_ (cm^3^/Ms × 10^–8^)
**Ir1**	8.07	3.66	1.92
**Ir2**	1.58	3.88	1.13
**Ir3**	2.01	3.16	0.78
**Ir4**	1.38	3.42	0.89
^a^ **Ir1**	4.28	0.96	0.48

a Stern–Volmer data using [(*p*–TMA)­Fe^II^–CO]^4+^.

**Ir1** possesses both a reducing excited-state reduction potential and significant spectral overlap between its photoluminescence spectrum and the absorption spectrum of [(*p*–TMA)­Fe^III^–Cl]^4+^ (Figure 3A), enabling both SET and EnT possibilities. The driving force for oxidative quenching is ∼−1.5 V and would produce [Ir­(ppy)_3_]^+^. Single-wavelength TA spectroscopy of a solution containing **Ir1** and [(*p*–TMA)­Fe^III^–Cl]^4+^ produces a positive signal that persists into the microsecond regime (λ^pump^ = 450 nm, λ^probe^ = 800 nm, Figure 3C). Spectroelectrochemical measurements conducted at +0.75 V vs S.C.E., a slightly more positive potential than the Ir^IV/III^ redox couple, demonstrates broad absorption features of **Ir1** ^**+**^ between 550–1000 nm (Figures 3C, inset, S17 and S18). This first quenching event can consequently be assigned to oxidative quenching, in accordance with the previously proposed mechanism, though the presence of EnT processes will be further examined (vide infra). Alternatively, TA spectroscopy of **Ir3** and **Ir4** in the presence of [(*p*–TMA)­Fe^III^–Cl]^4+^ yields no detectable redox products that could be assigned to oxidative or reductive quenching (Figures 3D, S19 and S20). We therefore assigned the quenching mechanism to an EnT process for **Ir3/Ir4**.

Considering the similarity in magnitude of the bimolecular rate constants, we calculated the overlap integrals between the emission spectral profiles of **Ir1**–**Ir4** and the absorption spectrum of [(*p*–TMA)­Fe^III^–Cl]^4+^ (Figure 3A), which together modulate the theoretical rate constant for a FRET process for a bimolecular, homogeneous EnT process (eq 1).^42,43^

1 $$k{\left(r\right)}_{FRET}=\frac{Q_{D}\kappa^{2}}{\tau_{o}r^{6}}\left(\frac{9000\mathrm{Ln}\left(10\right)}{128\pi^{5}Nn^{4}}\right)\int_{\lambda }^{\lambda +d\lambda }F_{D}\left(\lambda \right)\varepsilon_{A}\left(\lambda \right)\lambda^{4}d\lambda$$

briefly, *k* (*r*)_FRET_ represents the theoretical magnitude of the observed FRET rate constant between a donor and acceptor at a fixed distance (*r*). For homogeneous, bimolecular FRET processes, various physical constants such as *r* and the dipole orientation factor (κ) are not fixed and consequently explicit calculation of *k* (*r*)_FRET_ becomes untenable. Nevertheless, the PS photoluminescence QY (*Q*_*D*_), exited-state lifetime (τ_o_), and overlap integral between the area normalized emission spectrum of the donor (*F*_D_ (λ)) and the absorption spectrum of the acceptor (ε_A_ (λ)) will modulate *k* (*r*)_FRET_ for bimolecular processes. As demonstrated by Kudisch et al., a FRET processes can be implicated among a set of PSs if the magnitude of the experimentally measured quenching rate constants increase or decrease concurrent with the numerous physical constants in eq 1. (i.e., *k*_p_, *F*_D_ (λ), ε_A_ (λ)), bypassing the need for explicit determination of *r* and κ.^44^

The product of the spectral overlap integrals (*J*_overlap_) with the radiative rate constant (*k*_*p*_, Figure S21, see Section S9 of the Supporting Information for further discussion) are remarkably similar and in excellent agreement with the experimentally measured *k*_*q*_ values available from Stern–Volmer analysis (Table 2). Specifically, a FRET analysis suggests that the measured quenching rate constants should be similar in magnitude, which is observed experimentally. Crucially, when comparing **Ir2**–**Ir4**, all experimentally measured values of *k*_*q*_ and *k*_*p*_ × *J*_overlap_ are within a factor of 2. Considering the substantial difference in the excited-state reduction potentials between **Ir1**–**Ir4** (Table S4), which would modulate the driving force for SET processes, oxidative or reductive quenching between Ir2*–Ir4* and [(*p*–TMA)­Fe^III^–Cl]^4+^ is less probable, because the magnitude of the quenching rate constants do not correlate well with the thermodynamic driving force for photoinduced SET. Such observations are more consistent with a common, energy-transfer quenching mechanism.^42^ We note that photoinduced oxidative quenching could still be possible and is thermodynamically accessible with **Ir2**–**Ir4**; however, the presented data suggests that EnT would have a larger rate constant than the photoinduced electron transfer, rendering EnT a higher quantum yield process. While a FRET mechanism appears dominant with **Ir2**–**Ir4**, the spectral overlap between Ir1* and [(*p*–TMA)­Fe^III^–Cl]^4+^ absorption suggests that FRET can still occur concurrently with photoinduced SET, with each respective process proceeding with their associated QYs. [(*p*–TMA)­Fe^III^–Cl]^4+^ can reasonably act as an EnT and SET acceptor given both the significant absorption spectral overlap with Ir1* and the highly exothermic thermodynamic driving force for SET (Δ*E* = −1.5 V). Indeed, the measured quenching rate constant is of reasonable magnitude to incorporate a FRET process (Table 2) and photoinduced SET. Further, while FRET appears as a plausible mechanistic pathway and has been observed between other Ir^III^ PSs and metalloporphyrin species,^46,47^ a Dexter EnT process cannot be entirely ruled out and could also contribute to the overall magnitude of the bimolecular quenching rate constants. While both EnT and SET are available mechanistic pathways at the reaction onset, mechanistic elucidation cannot be determined with time-resolved spectroscopy alone.

To investigate any excited-state reactivity of [(*p*–TMA)­Fe^III^–Cl]^4+^, we performed femtosecond (fs) TA spectroscopy in acetonitrile (Figures 3E,F, S22 and S23).^42^ At λ^pump^ = 680 nm, [(*p*–TMA)­Fe^III^–Cl]^4+^* transient excited-state absorption (ESA) and ground-state bleach (GSB) signals are well fit using two decay components with 0.9 and 3.0 ps time constants, which we attributed to internal conversion/vibrational relaxation followed by ground-state recovery (Figure 3E). Similar time constants are observed for monomeric [(*p*–TMA)­Fe^III^–Cl]^4+^ in aqueous media.^48^ While this short-lived excited state precludes bimolecular diffusion-based SET to [(*p*–TMA)­Fe^III^–Cl]^4+^*, photochemical reactivity for CO evolution is observed in control experiments without PS, in accordance with previous reports (Figure 2A, Table S1).^29^ We consequently conducted fs-TA measurements with λ^pump^ = 400 nm, a higher-energy wavelength more similar to our experimental excitation conditions, and observed more complex transient dynamics that ultimately yielded a long-lived photoproduct that existed beyond the time scales of our measurements (Figure 3F). ns-TA spectroscopy following λ^pump^ = 450 nm, the experimental wavelength used to drive the photocatalysis (Figure S9B), also shows a long-lived product that is not well fit with a single exponential decay (Figure S24). Ligand-to-metal charge transfer (LMCT) excited states of high valent ferric species are well documented and often undergo homolytic photolysis of the metal–ligand bond.^49,50^ At higher pump energies, LMCT photolysis of the Fe^III^–Cl bond would yield Fe^II^ and Cl^•^, albeit with a small QY given the low intensity of these photoproducts in the ultrafast TA and the likely strongly overlapping absorption from the Soret band. Inclusion of **Ir1** in the photocatalytic systems clearly enhances the TON for CO evolution (Figure 2A). We therefore conclude that oxidative quenching represents a higher QY pathway at the reaction onset.

The system using **Ir2** proceeds with a much higher QY that operates through a reductive quenching pathway since [TEA] ≫ [Fe]_total_. Photochemical systems using **Ir3**, which is an analogous heteroleptic complex that is generally a stronger photooxidant compared to the homoleptic **Ir1**, demonstrates a nearly identical kinetic profile with **Ir2** and consequently a similar mechanism (Figure 2A).^51^ The ability to form the reduced PS consistently allows for rapid access to the required [(*p*–TMA^••^)­Fe^II^]^2+^ species needed for CO_2_ binding. Triply reduced [(*p*–TMA^••^)­Fe^II^]^2+^ is described as an Fe^II^ center with a doubly reduced porphyrin structure.

### Further Photochemical Transformations

Since TEA does not quench Ir1* emission, we wanted to consider what interactions occur between Ir1* and the various Fe-porphyrin species involved in the CO_2_RR. Following the initial oxidative quenching event, **Ir1** ^**+**^ and Fe^II^ are formed, and regeneration of **Ir1** occurs through a dark SET from TEA to yield TEA^+^. Deprotonation of the α–C–H bond results in the formation of a strongly reducing α-amino radical (∼−1.58 V vs S.C.E.^52^), which can facilitate further reduction of the CO_2_RR catalyst to the required [(*p*–TMA^••^)­Fe^II^]^2+^ state without the need for further oxidative quenching pathways. Protonation of the [(*p*–TMA)­Fe^II^–CO_2_]^2+^ species can result in cleavage of the C–O bond and the formation of water and the ferrous–carbonyl intermediate, which was proposed as a key species on-cycle for CH_4_ photoevolution (Scheme S1). However, ferrous-carbonyl intermediates (e.g., [(*p*–TMA)­Fe^II^–CO]^4+^) are challenging to isolate since they require a constant CO environment to maintain CO coordination, which consequently hinders investigations into the photochemical interactions with the PS.

We consequently sought to access [(*p*–TMA)­Fe^II^–CO]^4+^ *in situ* and perform Stern–Volmer analysis and TA spectroscopy by leveraging the observed wavelength-dependent reactivity in the fs-TA (Figure 3E,F). While minimal changes in the UV–vis spectrum of [(*p*–TMA)­Fe^III^–Cl]^4+^ are observed upon continued irradiation with 370 nm light in an argon headspace (Figure S25), supporting a reversible photolysis process, significant spectral evolution is observed in a CO headspace during irradiation (Figure 4A). We attribute this to the strong binding of CO to [(*p*–TMA)­Fe^II^]^4+^ that yields photogeneration of [(*p*–TMA)­Fe^II^–CO]^4+^. Cyclic voltammetry reveals a transition from a reversible Fe^III/II^ couple in an argon headspace into an irreversible Fe^III^ wave under a CO headspace, consistent with previous observations in related porphyrins and enhanced oxidative stability of [(*p*–TMA)­Fe^II^–CO]^4+^, which suppresses the photolysis back reaction (Figure 4B,C).^16^ This can tentatively be attributed to a transition from a high-spin *S* = 2 ferrous intermediate to a low-spin *S* = 0 upon coordination of CO.^33^ Finally, to assign the spectral evolution to the formation of a ferrous–carbonyl intermediate, spectroelectrochemical measurements were conducted at a fixed potential of −0.5 V vs S.C.E. in a CO environment. These conditions ensure that [(*p*–TMA)­Fe^II^]^4+^ is generated in the presence of CO. This experiment yields a spectrum that demonstrates excellent agreement with the terminal spectra measured following irradiation of [(*p*–TMA)­Fe^III^–Cl]^4+^ in CO (Figure 4A) as well as previous literature reports.^53^ Stern–Volmer analysis was consequently performed using time-resolved emission data collected in a CO headspace to quantify the interaction between Ir1* and [(*p*–TMA)­Fe^II^–CO]^4+^, which yields a bimolecular rate constant of 4.28 × 10^9^ M^–1^ s^–1^ (Figures 4D and S26). Ferrous-carbonyl complexes are well-known to undergo photodissociation of CO following visible light excitation.^54–56^ Single-wavelength ns-TA measurements at 550 and 600 nm probe wavelengths following a 450 nm pump excitation probes the bleach of [(*p*–TMA)­Fe^II^–CO]^4+^ and formation of [(*p*–TMA)­Fe^II^]^4+^, respectively (Figure 4E). These long-lived signals can be adequately fit with a single exponential decay, consistent with a pseudo-first order decay of [(*p*–TMA)­Fe^II^]^4+^ in the CO saturated solution (i.e., [CO] ≫ [(*p*–TMA)­Fe^II^]^4+^), in agreement with previous measurements (*k*_obs_^550^ = 420 μs^–1^, *k*_obs_^600^ = 342 μs^–1^)).^54–56^ These long-lived components complicate measurement of diagnostic signatures of **Ir1** ^**+**^ that would form if another oxidative quenching mechanism arises between Ir1* and [(*p*–TMA)­Fe^II^–CO]^4+^. However, if [(*p*–TMA^•^)­Fe^II^]^3+^ and **Ir1** ^**+**^ are both generated following the ns pulse, the decay probed at 600 nm (which is a diagnostic peak wavelength for both [(*p*–TMA)­Fe^II^]^4+^ and **Ir1** ^**+**^, Figure 3C) would require a multiexponential fit since the only viable route to regenerate ground state **Ir1** would be through bimolecular back electron transfer between **Ir1** ^**+**^ and [(*p*–TMA^•^)­Fe^II^]^3+^.^57^ The decay measured at 600 nm for a solution of **Ir1** and [(*p*–TMA)­Fe^II^–CO]^4+^ remains well fit by a single exponential decay, implicating an EnT mechanism and a shift in the excited state quenching mechanism from SET to EnT when [(*p*–TMA)­Fe^II^–CO]^4+^ builds up in solution (Figure 4F, *k*_obs_^600^ = 194 μs^–1^, *R* ^2^ = 0.999). The origin of this shift, despite SET remaining thermodynamically viable, likely arises from the nearly 1 V loss of driving force for the SET to [(*p*–TMA)­Fe^III^–Cl]^4+^ compared to [(*p*–TMA)­Fe^II^–CO]^4+^ (Figure 4C). Spectroelectrochemical data suggest [(*p*–TMA)­Fe^II^–CO]^4+^ still absorbs strongly across the visible region where Ir1* emits, and the magnitude of this interaction is smaller compared to [(*p*–TMA)­Fe^III^–Cl]^4+^ but matches better with the assigned EnT reactions between **Ir2**–**Ir4** and [(*p*–TMA)­Fe^III^–Cl]^4+^ (Table 1), consistent with an EnT mechanism.

**Figure 4:**
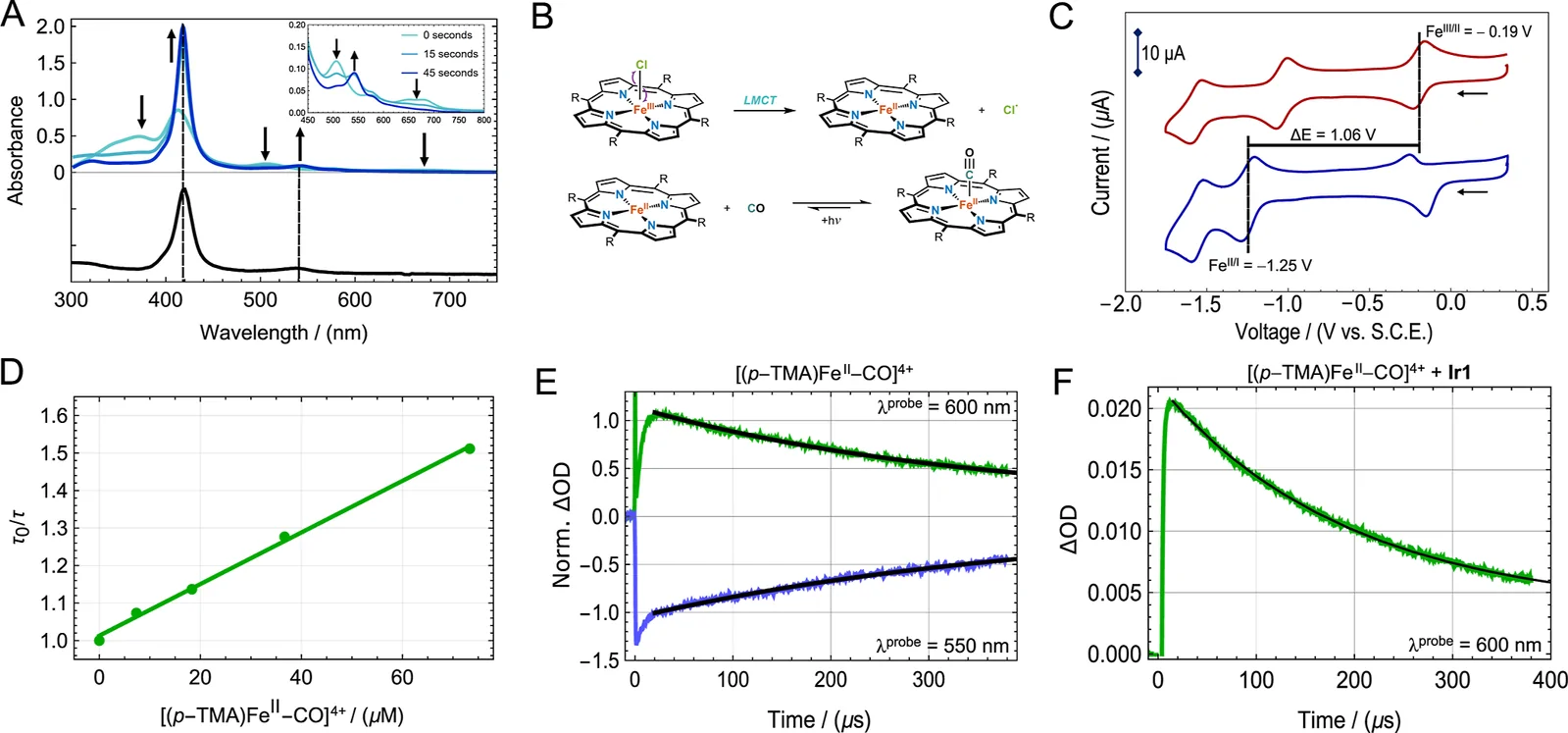
*In situ* formation, electronic structure, and excited-state reaction pathways of [(*p*–TMA)­Fe^II^–CO]^4+^. (A) Spectral evolution of a solution of [(*p*–TMA)­Fe^III^–Cl]^4+^ in a CO environment during 370 nm irradiation. Black trace: measured spectra from spectroelectrochemistry of a [(*p*–TMA)­Fe^III^–Cl]^4+^ solution at −0.5 vs S.C.E. in a CO headspace. (B) LMCT photolysis of the Fe^III^–Cl followed by reversible CO bonding. (C) Cyclic voltammetry of [(*p*–TMA)­Fe^III^–Cl]^4+^ in an argon (red) and CO (blue) headspace using TBA^+^ PF_6_^–^ as the supporting electrolyte. Scan rate: 100 mV/s. (D) Stern–Volmer analysis obtained using time-resolved emission data between Ir1* and [(*p*–TMA)­Fe^II^–CO]^4+^. (E) Single-wavelength nanosecond TA of [(*p*–TMA)­Fe^II^–CO]^4+^ following a 450 nm pump. λ^probe^: 600 nm (green)/λ^probe^: 520 nm (blue). (F) Single-wavelength nanosecond TA of [(*p*–TMA)­Fe^II^–CO]^4+^ and **Ir1** following a 450 nm pump. λ^probe^: 600 nm, data is well fit using a single exponential decay.

We were then curious to uncover a general mechanism for both CH_4_ and C_2_H_6_ production observed herein, since introduction into a CH_4_ photoevolution cycle does not arise from an oxidative quenching step. Unlabeled CH_4_ has been observed using [(*p*–TMA)­Fe^III^–Cl]^4+^ as an electrocatalyst in DMF, which was proposed to arise from hydrolysis of the peripheral methyl-anilinium groups.^16^ Recent work by Mitchell et al.^58^ has implicated reactivity of reduced iron­(tetraphenylporphyrinato) chloride ([Fe-TPP]) with the tetraalkyl ammonium salts that are often used as supporting electrolytes in organic solutions. [(*p*–TMA^••^)­Fe^II^]^2+^ could react in a similar manner with the trimethylanilinium groups in the porphyrin structure. Cyclic voltammetry experiments beginning at the open circuit potential exhibit three reversible waves that are attributed to the Fe^III/II^ couple at −0.19 V, then two porphyrin centered reductions at −1.04 V and −1.56 V (V vs S.C.E., Figure 4C).^59^ In a similar fashion to the experiments performed by Mitchell et al., holding the potential at −1.85 V for various times followed by an anodic scan yields growth of a new feature in the CV ca. −0.65 V (Figure 5). This observation may be attributable to Fe^II^-alkyl intermediates that are only formed after prolonged anodic potential holding. Crucially, when [(TPP)­Fe^III^], the structural analog without the anilinium functional groups, is exposed to the same conditions, no additional features appear in the cyclic voltammograms (Figure S27C). Attack of the methyl-anilinium groups by [(*p*–TMA^••^)­Fe^II^]^2+^ likely yields a [(*p*–TMA)­Fe^II^–CH_3_]^4+^ species (Scheme 1A, top). CV cycling of samples containing [(*p*–TMA)­Fe^II^–CH_3_]^4+^ attenuates the [(*p*–TMA)­Fe^II^–CH_3_]^4+^ oxidative feature, implicating reduction and protonation of [(*p*–TMA)­Fe^II^–CH_3_]^4+^ as a plausible CH_4_ production mechanism (Figure S27D). The photoevolution of C_2_H_6_ can similarly be rationalized by the attack of TEA-H by [(*p*–TMA^••^)­Fe^II^]^2+^ to yield a similar Fe^II^-ethyl intermediate (Scheme 1A, bottom). This process likely competes with the formation of Fe^II^–H intermediates that are responsible for the HER. TEA-H is readily formed following oxidation of TEA to yield TEA^•+^, which possesses a strongly acidic α–C–H bond that is readily deprotonated by unreacted TEA to yield TEA-H and the α-amino radical.^60^ Ethane formation increases with increasing [TEA] (Figure S6), consistent with this mechanistic picture.

**Figure 5:**
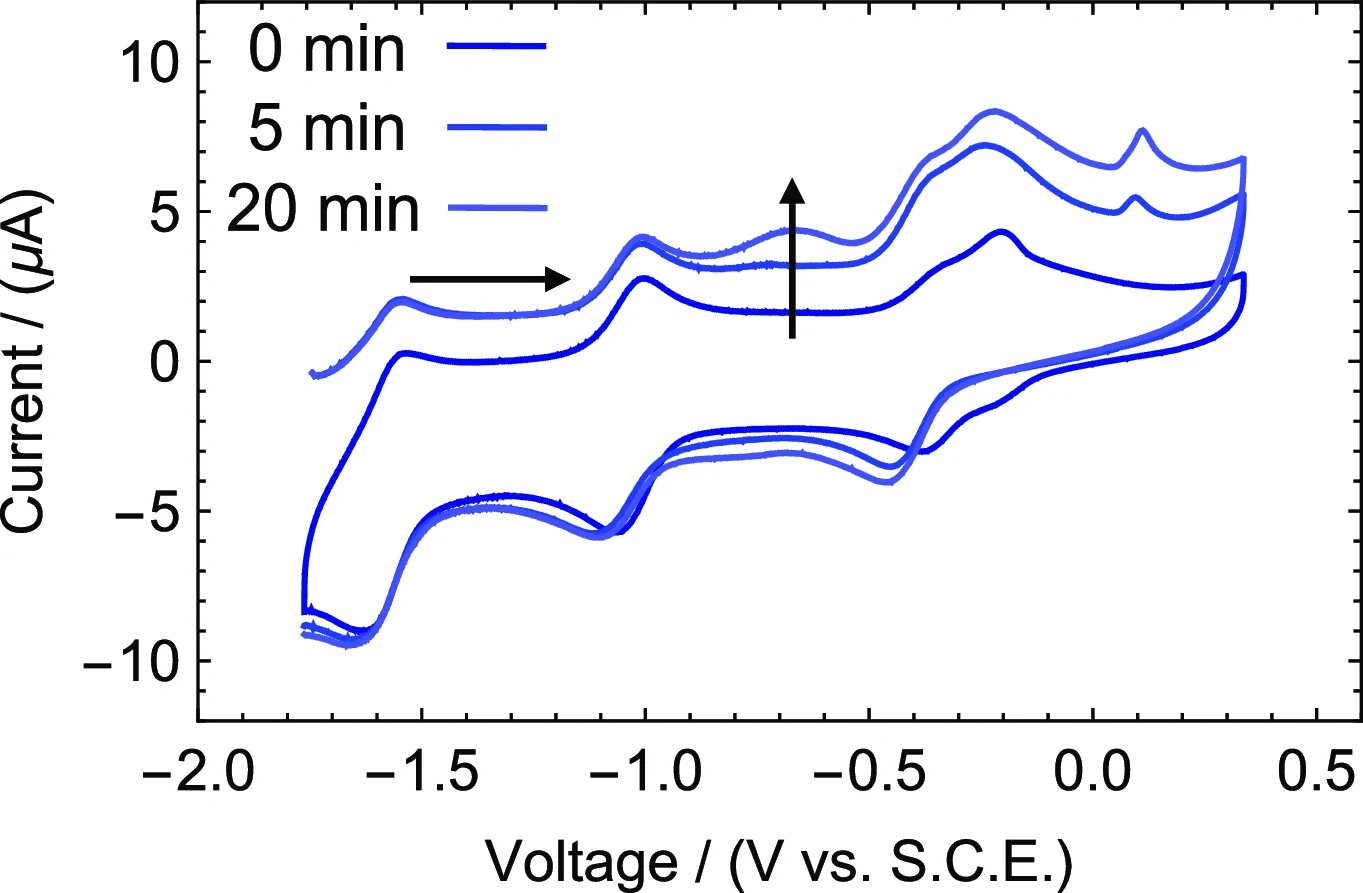
Cyclic voltammetry of [(*p*–TMA)­Fe^III^–Cl]^4+^ with different holding potential times at −1.8 V with a KPF_6_^–^ supporting electrolyte in DMF. Scan Rate: 100 mV/s.

**Scheme 1:**
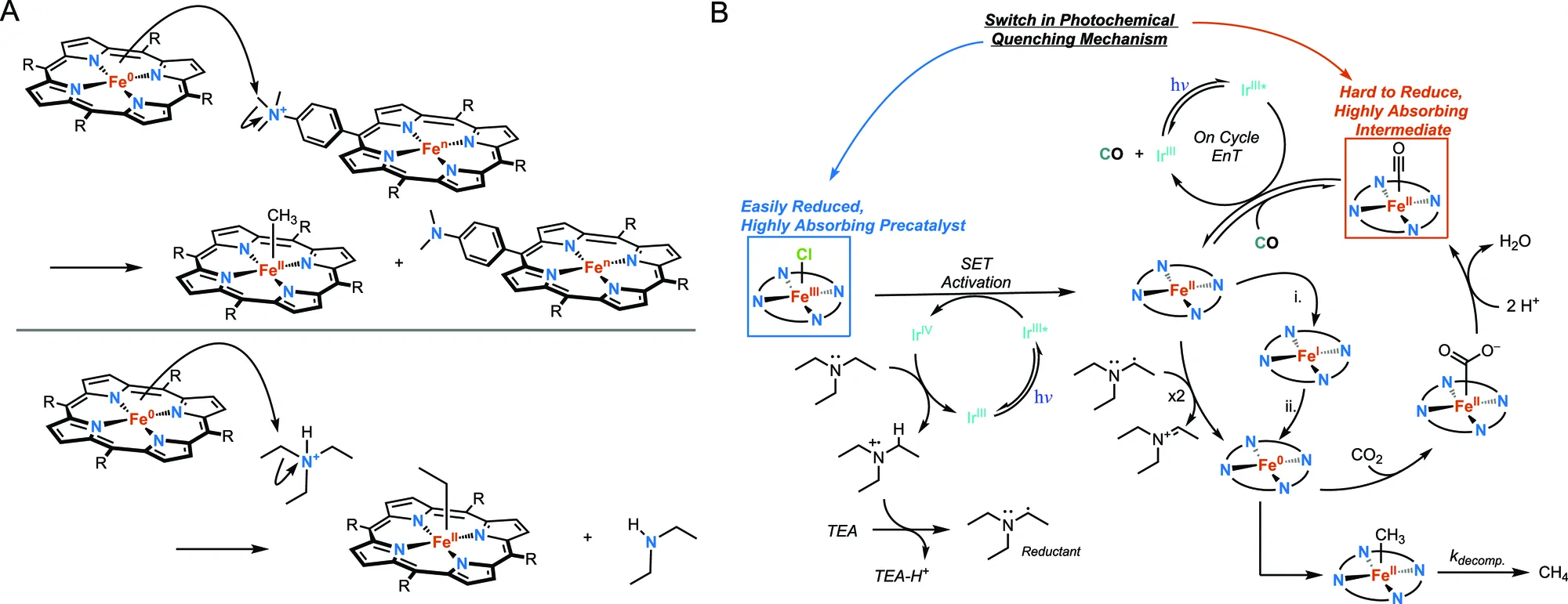
Photochemical Pathways and Mechanisms for the CO_2_RR Studied here; (A) Route for Unlabeled Highly Reduced Product Formation, Note: Fe^0^ is Depicted for Simplicity but is Equivalent with [(*p*–TMA^••^)­Fe^II^]^2+^; (B) Proposed Mechanism for the Photochemical CO_2_RR Using **Ir1**

## Discussion

CO photoevolution using **Ir1** (the highly reducing PS used in the original work^31^) begins with oxidative quenching of Ir1* by [(*p*–TMA)­Fe^III^–Cl]^4+^ to yield [(*p*–TMA)­Fe^II^]^4+^ and an α-amino radical following a thermal SET between **Ir** ^**+**^ and TEA (Scheme 1B). Since the rate of CO photoevolution is strongly dependent on [TEA], concurrent with the fact that the formation of TEA^•^ proceeds more rapidly at higher concentration of base, it is plausible that TEA^•^ facilitates reduction to the required [(*p*–TMA^••^)­Fe^II^]^2+^ state needed for CO_2_ binding (Figure S28). Whether or not two equivalents of TEA^•^ drive both reductions of [(*p*–TMA)­Fe^II^]^4+^ to [(*p*–TMA^••^)­Fe^II^]^2+^, or if Ir1* (pathways i, ii) also participates remains challenging to elucidate given the inability to access [(*p*–TMA)­Fe^II^]^4+^. Once [(*p*–TMA^••^)­Fe^II^]^2+^ is accessed, CO_2_ binding and subsequent protonation yields [(*p*–TMA)­Fe^II^–CO]^4+^, which acts as a FRET acceptor quenching Ir1* photoluminescence. FRET or direct excitation breaks the Fe^II^–CO bond. Transient [(*p*–TMA)­Fe^II^]^4+^ must be reduced before CO coordination, which likely acts as the QY limiting step. At this point, if TEA^•^ acted as the only reductant capable of accessing [(*p*–TMA^••^)­Fe^II^]^2+^ from [(*p*–TMA)­Fe^II^]^4+^, the reaction would stall, as no equivalents of TEA^•^ would exist in solution at this point in reaction progress. We therefore tentatively assign a photoinduced oxidative quenching of Ir1* by [(*p*–TMA)­Fe^II^]^4+^ to furnish [(*p*–TMA^•^)­Fe^II^]^3+^ and another equivalent of TEA^•^ to allow the mechanistic cycle to recur. Notably, the coordination of CO remains pivotal in changing the driving force for SET, altering the dominant excited-state interaction from SET to FRET.

The photoevolution of CH_4_, albeit not catalytic, yet consistently observed, arises from degradation of the methyl-anilinium groups. This observation does not rule out the ability to form CH_4_ as a CO_2_RR product. However, it does not appear likely that the CH_4_ cycle begins by oxidative quenching of Ir1* by [(*p*–TMA)­Fe^II^–CO]^4+^. Further, sequential PCET steps where the electron is supplied by the PS and the proton from the exogenous acid further remains unlikely. An alternative pathway could involve the ability of oxidized amines to act as concerted hydrogen atom donors, as these reactions required such electron donors to observe methane in previously reported works.^31,32^ Harnessing the reactivity of the oxidized imine, as it is a highly reactive intermediate, is not only challenging but also difficult to reproduce, as their steady-state concentrations will depend on numerous factors that are often not standardized (e.g., photoreactor geometry and photon flux).^61^ Future work from our group will seek to further understand various PCET reactivity pathways available to ferrous–carbonyl intermediates.

## Conclusion

The photochemical CO_2_RR presents an attractive opportunity that harnesses photon energy to create reactive intermediates *in situ* capable of breaking thermodynamically stable carbonyl bonds to yield valuable reduced products. Dual catalytic systems are often comprised of a CO_2_RR catalyst and a PS, where the latter is tasked with light absorption and shuttling energy and/or electrons via photoredox mechanisms between sacrificial electron donors and the catalyst. Highly active systems that result in the photoevolution of desirable products are highly sought after. However, realization of such systems is continually hindered, as the multiple H^+^/e^–^ transfer steps and formation of highly reactive intermediates lead to multiple possible deleterious deactivation steps/mechanisms. With various oxidation states available to both the PS and the CO_2_RR catalyst alongside highly reactive radical intermediates, the available photochemical mechanistic steps can change as a function of reaction progress, and photoevolution of products that are desirable, but do not explicitly arise from CO_2_ reduction, can occur.

Our results show that a highly desired C_2_ product from CO_2_RR, ethane, forms in the presence of PS and catalyst but does not arise from the CO_2_RR. TEA, one of the most frequently adopted sacrificial electron donors in solar energy conversion, likely decomposes following oxidation to yield C_2_H_6_ as the terminal product. Further, in solar energy conversion systems where PS* is predominately quenched by the CO_2_RR catalyst and not the sacrificial electron donor, alterations in the photochemical mechanistic steps become possible. As the CO_2_RR catalyst proceeds to lower oxidation states during CO_2_RR cycles, the driving force for SET also lowers. The delicate balance between thermodynamic driving force and proclivity for EnT processes when using transition metal CO_2_RR catalysts, especially those that strongly absorb light, creates a dynamic mechanistic landscape.

## Supplementary Material



## Notes

This material is available free of charge at. Optimization studies, GC-MS results, time-resolved photoluminescence data, cyclic voltammetry, ultrafast transient absorption spectroscopy, spectroelectrochemistry, and NMR data (PDF)
