# Supplementary material for: Elucidating the Role of Energy- and Electron-Transfer Processes and the Route to Highly Reduced Products in the Dual IrIII/FeIII Photocatalyzed CO2 Reduction Reaction
Source: J Am Chem Soc. 2026 Sep 16;148(38):41089–98. doi: 10.1021/jacs.6c11427 (PMC13632555; doi:10.1021/jacs.6c11427)
Supplement: Supplementary file 1 [file ja6c11427_si_001.pdf]

# Supporting Information

## Elucidating the Role of Energy- and Electron-Transfer Processes and the Route to Highly Reduced Products in the Dual $Ir^{III}/Fe^{III}$ Photocatalyzed $CO_2$ Reduction Reaction

Stephen P. DiLuzio, Keon Ha Hwang, Kaitlin M. Luedecke, Erica Sutcliffe, Ryan G. Hadt*

Division of Chemistry and Chemical Engineering, Arthur Amos Noyes Laboratory of Chemical Physics, California Institute of Technology, Pasadena, California 91125, United States

*Corresponding Author: rghadt@caltech.edu

# Table of Contents

## *Section 1:*

Materials and Instrumentation……….…………………………………………………….…S5-S7

Experimental………………..……….………………………………………………...…..S8-S9

## *Section 2: Optimization Studies*

**Scheme S1**: Photo-driven $CO_2$ reduction to $CH_4$ proposed mechanism……....………...……..S10

**Figure S1:** Moles of photoproduct as a function of [$Fe^{III}$-p-TMA] for **Ir1**…….………...……..…S11

**Figure S2:** Moles of photoproduct as a function of [TEA] for **Ir1**…………………….……......…S12

**Figure S3:** Moles of photoproduct as a function of [TFE] for **Ir1**…………………..……....…S13

**Figure S4:** Moles of photoproduct as a function of [**Ir1**]… ………… ………………………..S14

**Table S1:** Optimized Conditions for **Ir1**……………………………………………………......S15

**Figure S5:** Moles of photoproduct as a function of [$Fe^{III}$-p-TMA] for **Ir2**…….………...……..…S16

**Figure S6:** Moles of photoproduct as a function of [TEA] for **Ir2**…………………….……......S17

**Figure S7:** Moles of photoproduct as a function of [TFE] for **Ir2**…………………..……....…S18

**Figure S8:** Moles of photoproduct as a function of [**Ir2**].… ………… ………………………..S19

**Figure S9:** Photoreactor geometry, emission spectra, and power dependence data.………..…S20

**Table S2:** Optimized Conditions for **Ir2**…………………………………………………..…S21

## *Section 3: GC-MS results and Sacrificial Electron Donor Table*

**Figure S10:** Mass spectra of $^{13}CO$ when using $^{13}CO_2$ as the carbon source.………...……...…S22

**Table S3:** Structures of SEDs used in this work……………………..………...………....S23

**Figure S11:** Mass spectra of $^{12}C_2H^6$ when using $^{13}CO_2$ as the carbon source.………..……....S24

## *Section 4: Stern-Volmer Analysis*

**Figure S12:** Stern-Volmer analysis from steady-state emission data using TEA and TEA-HCl as quenchers……………………………………………………………………...……......S25

**Figure S13:** Stern-Volmer analysis using time-resolved emission data between **Ir1** and [$Fe^{III}$-p-TMA]…..………………………………………………………………….....……...S26

**Figure S14:** Stern-Volmer analysis using time-resolved emission data between **Ir2** and [$Fe^{III}$-p-TMA]…..…………………………..………………………………………...……....S27

**Figure S15:** Stern-Volmer analysis using time-resolved emission data between **Ir3** and [$Fe^{III}$-p-TMA]……..……………………………………………………………………...……..S28

**Figure S16:** Stern-Volmer analysis using time-resolved emission data between **Ir4** and [$Fe^{III}$-p-TMA]……...…………………………………………………………………...……..…S29

## *Section 5: Cyclic Voltammetry and Spectroelectrochemistry of the PSs*

**Figure S17:** Oxidative spectroelectrochemical spectra of **Ir1……..**………………...……...…S30

**Figure S18:** Oxidative and reductive spectroelectrochemical spectra of **Ir2…**……...……......S31

**Figure S19:** Oxidative and reductive spectroelectrochemical spectra of **Ir3…**……...………..S32

**Figure S20:** Oxidative and reductive spectroelectrochemical spectra of **Ir4…**……...……..…S33

**Figure S21:** Absorption and emission spectra and associated PLQY of **Ir3** and **Ir4.**…………S34

**Table S4:** Excited state reduction potentials of **Ir1** – **Ir4….**……………………………......S35

## *Section 6: Transient Absorption Spectroscopy*

**Figure S22:** Visible fs-TA spectroscopy of [$Fe^{III}$-p-TMA] following a 680 nm pulse……......S36

**Figure S23:** Visible fs-TA spectroscopy of [$Fe^{III}$-p-TMA] following a 400 nm pulse.....….....S37

**Figure S24:** ns-TA spectroscopy of [$Fe^{III}$-p-TMA] following a 450 nm pulse……..…..….....S38

**Figure S25:** Change in UV-vis of [$Fe^{III}$-p-TMA] following 390 nm irradiation...…..…........S39

**Figure S26:** Stern-Volmer analysis using time-resolved emission data between **Ir1** and [$Fe^{II}$-p-CO]……..…………………………………….………………………...……......S40

## *Section 7: Cyclic Voltammetry of the $CO_2$RR catalyst*

**Figure S27:** Cyclic voltammetry of [$Fe^{III}$-p-TMA] and [$Fe^{III}$-TPP] with various supporting electrolytes.. ……………………………………………………...……..........................S41

**Figure S28:** Cyclic voltammetry of [$Fe^{III}$-p-TMA] in a $CO_2$ environment…………….........S42

## *Section 8: Inner Filter Effects*

**Figure S29:** Steady-state photoluminescence and correction factors for associated Stern-Volmer analysis between **Ir1** and [$Fe^{III}$-p-TMA]……….……………………………...…..S43-S44

# *Section 9: Calibration Data*


**Figure S30:** FRET analysis……………………………………………………………………………S45


# *Section 11: Calibration Data*


**Figure S31:** GC calibration curves of $H_2$, CO, $CH_4$. $C_2H_4$, and $C_2H_6$…………………………S46

**Figure S32:** GC-MS calibration curves of CO, $CH_4$. $C_2H_4$, and $C_2H_6$…………………………S47


# *Section 12: Synthesis and NMR Data*


Synthesis of **Ir3**……………………………………………………………………………………S48

**Figure S33:** $^{1}$H NMR of **Ir3**……………………………………………………………………S49

**Figure S34:** $^{13}$C NMR of **Ir3** ……………………………………………………………………S50

Synthesis of **Ir4**……………………………………………………………………………………S51

**Figure S35:** $^{1}$H NMR of **Ir4**……………………………………………………………………S52

**Figure S36:** $^{13}$C NMR of **Ir4**……………………………………………………………………S53

Synthesis of $(TBA)_2Ox^{2-}$……………………………………………………………………………S54

Synthesis of [(*p*-TMA)$Fe^{III}$-Cl] ……………………………………………………………………S55

Photochemical synthesis of [(*p*-TMA)$Fe^{II}$-CO]………………………………………………………S56

**Refereneces**…………………………………………………………………………………………S57

# *Section 1:*

# *Materials and Instrumentation*

**Materials** All commercial chemicals were used as received. $H_2$–*p*–TMA was purchased from Frontier Specialty. Mohrs salt was purchased from Fischer Scientific. [Ir(ppy)$_3$] and [Ir(dFCF$_3$ppy)$_2$(dtbbpy)]$^+$PF$_6^-$ were purchased from TCI America and Strem chemicals, respectively. Tetrabutylammonium hexafluorophosphate and sodium hexafluorophosphate were purchased from Sigma-Aldrich. Dichlorotetrakis(2-(2-pyridinyl)phenyl)diiridium(III) ([(ppy)$_2$Ir(Cl)]$_2$) was purchased from ChemScene. 1,10-phenanthroline and 3,4,7,8-tetramethylphenanthroline were purchased from Alfa Aesar and Ambeed, respectively. 2,2,2-Trifluoroethanol was purchased from Sigma Aldrich. Triethylamine was purchased from thermo scientific. Diisopropylamine (DIPEA) was purchased from Sigma-Aldrich. 1,4-Diazabicyclo92.2.2]octane (DABO) was purchased from Sigma-Aldrich. Triethanolamine (TEOA) was purchased for Fisher Scientific. 1,3-dimethyl-2-phenyl-2,3-dihydro-1H-benzo[d]imidazole (BIH) was purchased from Ambeed. Tetrabutylammonium Bromide was purchased from TCI. Triethylamine Hydrochloride was purchased from TCI. Triethylamine-$d_{15}$ was purchased from Sigma-Aldrich. Tetrabutylammonium methoxide solution (20%) in methanol was purchased from Sigma-Aldrich. Oxalic acid dihydrate was purchased from Thermoscientific. 5,10,15,20-Tetraphenyl-21*H*,23*H*-porphine iron(III) chloride ([Fe$^{III}$(TPP)] was purchased from Sigma-Aldrich. Bulk acetonitrile (ACN) and Dimethylformaldehyde (DMF) solvent was purchased from Fischer chemical. Isotopically labeled 1 L tanks of $^{13}CO_2$ were purchased from Sigma-Aldrich. All NMR spectra were collected on a Bruker 500 MHz spectrometer and referenced to individual solvent peaks.

**Steady-State UV-vis Absorption Spectroscopy** UV-vis spectra were measured on a Cary 5000 spectrometer in acetonitrile using 1 cm pathlength quartz cuvette.

**Steady-State Emission Spectroscopy** Steady-state corrected emission spectra were recorded on a Jobin S4 Yvon Spec Fluorolog-3-11. Sample excitation was achieved via a xenon arc lamp with wavelength selection provided by a monochromator. Right angle emission was sorted using a monochromator and detected with a Hamamatsu R928P photomultiplier tube with photon counting. Samples were prepared inside an mBraun glovebox using Starna Cell quartz spectroscopic cuvettes fitted with air-tight PTFE piston seals in acetonitrile.

**Single Wavelength Nanosecond Transient Absorption Spectroscopy** Nanosecond time-resolved spectroscopic measurements were performed in the Beckman Institute Laser Resource Center at California Institute of Technology. The experimental setup has been described previously.[1] Laser excitation was provided by pulses of 8 ns duration from a Q-switched Nd:YAG laser (Spectra-Physics Quanta-Ray PRO-Series) operating at 10 Hz. The third harmonic was used to pump an optical parametric oscillator (OPO, Spectra-Physics Quanta-Ray MOPO-700) tunable in the visible region to provide laser pulses at 450 nm, which was selected as the pump excitation

wavelength to minimize overlapping absorption with the Soret band of the $CO_2$ reduction catalyst. Samples were continuously stirred throughout the duration of the experiment and difference spectra were averaged over approximately 100 shots. An 800 nm probe wavelength was used for data collection on samples containing [(p-TMA)$Fe^{III}$-Cl] to minimize absorption effects of [(p-TMA)$Fe^{III}$-Cl]. For [(p-TMA)$Fe^{II}$-CO], a 600 nm probe was used since **Ir1**$^+$ absorbs strongly at 600 nm and the [[(p-TMA)$Fe^{II}$-CO] catalyst does not absorb as strongly in the low energy region of the visible spectrum. To investigate the FRET mechanism for the heteroleptic complexes, **Ir3** and **Ir4** were used. **Ir2**, which has a more positive excited-state reduction potential, has known capabilities of oxidizing chloride anions[2] that act as the counter anions for [(p-TMA)$Fe^{III}$-Cl]. These competitive quenching pathways will complicate the observed single-wavelength transient decays. Between **Ir1**, **Ir3**, and **Ir4**, the[(p-TMA)$Fe^{III}$-Cl] was kept at 50 μM. This value was used as an optimum value that will afford observation of redox products if formed without inducing inner filter effects that would otherwise occur at higher concentrations of metalloporphyrin.

**Ultrafast Transient Absorption Spectroscopy** Ultrafast laser pulses used for the transient absorption (TA) measurements originate from a Coherent Astrella Ti:Sapphire amplifier system, generating 40 fs pulses centered on 800 nm at a 1 kHz repetition rate. These pulses were passed into an Ultrafast Systems Helios spectrometer system to carry out the transient absorption spectroscopy measurements. Pulses from the Astrella were attenuated and then delayed by the Helios' built-in 7 ns delay stage before undergoing supercontinuum generation in sapphire to generate a white-light (450 – 750 nm) probe. Most of the beam was focused onto the sample and then subsequently into a fiber spectrometer, while the remaining portion of the beam bypassed the sample and was focused into a second fiber spectrometer to act as a reference.

Pump pulses were generated through various means using a stronger portion of the Astrella output than that of the probe. To generate 680 nm pump pulses, a Coherent OPerA optical parametric amplifier was used. For pumping at 400 nm, the laser fundamental was frequency doubled in a β-barium borate (BBO) crystal (EKSMA Optics, 10 mm x 10 mm x 0.2 mm, $\theta = 29.2^o$, $\phi = 90^o$, P/P@400-800 nm) crystal. After wavelength manipulation, the pump was chopped, attenuated with a variable neutral density filter, depolarized, focused onto the sample, and subsequently blocked. Scattered pump light was minimized using spectral filters (ThorLabs FELH0450, FESH0650). A magnetic stirrer was also used to stir the sample over the course of the measurement.

Data were acquired using the Helios control software and subsequently exported to a custom MATLAB script for processing. Each datapoint was averaged for 2 s (1000 total pump-probe cycles) and an exponentially-spaced time-array was used to capture the decay of the signal. Each scan was repeated five times. Background points collected before time-zero where no signal was present were subtracted from the remaining data to remove pump scatter and other unwanted effects. Following this, the chirp in the data was corrected by tracking the position of the center of the cross-phase modulation (XPM) feature across the first picosecond of the spectrum and

resampling the data with the original time array to remove the chirp. The data were then globally fit through nonlinear least-squares to a series of exponentials convoluted with a gaussian instrument response function. In most cases, the XPM was many times larger than the actual signal and so greatly biased the fit. In this work our interest lies with the dynamics after the first picosecond so the signal between -0.15 and 0.15 ps was excluded from the fit. The fitting procedure yields several fitted time constants alongside the decay-associated spectrum (DAS) corresponding to each exponential decay.

**Cyclic Voltammetry and Spectroelectrochemistry** Samples were prepared in either acetonitrile or N,N-dimethylformamide (1 mM) with 100 mM tetrabutylammonium hexafluorophosphate supporting electrolyte. All samples were sparged with an argon stream for 10 minutes before measurements. Cyclic voltammograms were collected using a 3-electrode system comprised of a glassy carbon working electron, a platinum counter electrode, and a silver wire reference electrode at scan rates of 100 mV/s or 200 mV/s and are *i*R compensated. Potentials were referenced to the ferrocenium/ferrocene redox couple.

Spectroelectrochemical measurements were conducted using an analogous 3-electrode system that instead used a honeycomb gold working electrode sandwich between a 1 mm quartz cuvette. UV-vis spectra were collected using a StellarNet Inc. Black Comet UV-vis and DWARF-Star Miniature NIR spectrophotometer using SL4 high power tungsten halogen and deuterium lamps that fed into a fiber optic cable, passing through the cuvette, and into a secondary fiber optic cable leading into the spectrophotometer. Spectra were recorded when the current response in the chronoamperometric trace reach a plateau.

**Gas Chromatography** Direct injection measurements were conducted using an SRI instruments GC equipped with a thermoconductivity detector (TCD) and a Flame Ionization detector (FID) outfitted with a methanizer to enable detection of low concentrations of CO, $CH_4$, $C_2H_4$, and $C_2H_6$. An Argon carrier gas was flushed through the six ft Haysep-D column and a temperature ramp from 40°C – 150°C was performed for each run. Calibration curves for $H_2$, CO, $CH_4$, $C_2H_4$, and $C_2H_6$ were generated through injection of known concentrations of gases. Headspace sample injections comprised of 200 µL aliquots were conducted using a 500 µL air-tight Hamilton syringe needle.

**Gas Chromatography Mass Spectrometry (GS-MS)** GC-MS experiments were performed on an Agilent 7820 GC coupled to an Agilent 5977E quadruple MS. Separation was carried out on a GS-CarbonPlot column in split mode (10:1 split ratio, 8 mL/min helium split flow) and temperature ramp from 40 °C – 150 °C was performed for each run. Electron ionization was performed at 70 eV with the transfer line, ion source, and quadruple maintained at 250 °C, 230 °C, and 150 °C, respectively.

# *Experimental*

*Photocatalytic $CO_2$ reduction:* Photochemical $CO_2$ reduction reactions were carried out in septum sealed Chemglass 2-dram vials equipped with a magnetic stir bar and placed into a cylindrical, home-built photoreactor that contained LED strips with $\lambda^{max}$ = 450 nm (**Figure S9A/S9B**). Stock solutions of [(*p*–TMA)$Fe^{III}$–Cl] and $Ir^{III}$ PS were made and requisite amounts of TFE and TEA were added, then diluted with ACN to yield final solutions with target concentrations for each optimized system. After dilution, all samples were sparged with $CO_2$ for ten minutes and the caps were wrapped with black electrical tape and placed symmetrically inside the photoreactor. All reactions were performed in triplicate.

*Turnover Number Calculations (TON):* TON with respect to [(*p*–TMA)$Fe^{III}$–Cl] were calculated using *eq. 1*:

$$TON_{Fe} = \frac{total\ moles\ of\ select\ photoproduct}{total\ moles\ of\ Fe} \qquad (1)$$

where the total moles of Fe under the optimized conditions for each PS was 0.04 μmoles. Total moles of $H_2$, CO, $CH_4$, $C_2H_4$, and $C_2H_6$ were measured using gas chromatography.

*Quantum Yield (QY, Φ) measurements:* The reaction QY for CO and $H_2$ photoevolution was calculated using *eq. 2*:

$$\Phi = \frac{2*total\ moles\ of\ gaseous\ product\ evolved}{total\ moles\ of\ photons\ aborbed} \qquad (2)$$

where the total amount of moles of CO/$H_2$ was measured using gas chromatography. The photon flux was measured using a method described previously by our group.[3-4] Solutions were prepared in a 1 cm quartz cuvette and irradiated for 1.5 hours using a 456 nm Kessel LED operating at 25% power efficiency and placed 12 cm away from the samples. A ThorLabs thermal power meter (Model #S425C) was used to measure the average irradiation power in the cuvettes (0.053 W) and was corrected for photon energy. The area normalized flux into the solution was 0.032 μeinsteins/sec. The corresponding QY for $H_2$ evolution was 0.35% for **Ir1** and 49.7% for **Ir2** under their respective optimized conditions.

*$^{13}CO_2$ labeling experiments* Samples were prepared using the respective optimized conditions for **Ir1** and **Ir2**. Each sample was first sparged with argon for five minutes to remove atmospheric oxygen, followed by $^{13}CO_2$ for five minutes. GC-MS headspace analysis was conducted after 24 hours for the **Ir1** samples and after two hours for the **Ir2** samples.

*Relative Photoluminescence Quantum Yield:* The relative photoluminescent QY for **Ir3** and **Ir4** were calculated using *eq. 3*:

$$\Phi_{Samp.} = \Phi_{Ref.} \frac{Abs_{Ref.}}{Abs_{Samp.}} * \frac{I_{Samp.}}{I_{Ref.}} \qquad (3)$$

where $\Phi_{Samp.}$ and $\Phi_{Ref.}$ are the relative QY for **Ir3/Ir4** and the reference, $[Ru(bpy)_3]^{2+}PF_6^-$ ($\Phi$ = 6.2%). Abs. represents the absorbance at the excitation wavelength (450 nm) and $I_{Samp.}/I_{Ref.}$ is the total integrated intensity of the corrected emission spectra for the sample and reference, respectively. The absorbance of all samples was kept below 0.2 to minimize inner filter effects.

*Stern-Volmer Analysis:* The absorbance of the chromophore was kept between 0.17-0.25 for all measurements. Samples were prepared inside the glovebox using Starna Cell quartz spectroscopic cuvettes fitted with air-tight PTFE piston seals with acetonitrile collected from a solvent purification system that underwent three free-pump-thaw cycles before glovebox storage. Samples prepared using [(*p*–TMA)$Fe^{II}$–CO] were alternatively prepared outside of the glovebox and spectroscopic data was collected using 1 cm quartz screw cap cuvettes with PTFE seals and were rigorously kept under a CO headspace. Samples were excited using a 450 nm pulse excitation with 8 ns pulse duration and transient emission was measured at each respective $\lambda^{max}$ of the photoluminescence spectral profile ($\lambda^{probe}$ (**Ir1**) = 520 nm, $\lambda^{probe}$ (**Ir2**) = 470 nm, $\lambda^{probe}$ (**Ir3**) = 600 nm, $\lambda^{probe}$ (**Ir4**) = 550 nm). Time-resolved Stern-Volmer analysis was conducted using *eq. 4*:

$$\frac{\tau_o}{\tau} = K_{SV}[Q] + 1 \qquad (4)$$

Where $\tau_o$ is the deaerated excited state lifetime, $\tau$ is the measured single exponential decay at concentration [$Q$], $K_{SV}$ is the Stern-Volmer slope, and [Q] is the concentration of [(*p*–TMA)$Fe^{III}$–Cl], [(*p*–TMA)$Fe^{II}$–CO], [TEA], or [TEAH–Cl]. The reported bimolecular quenching rate constants are calculated through division of $K_{SV}$ by the experimental $\tau_o$. Stern-Volmer analysis using steady-state emission data was analogously conducted using

$$\frac{I_o}{I} = K_{SV}[Q] + 1 \qquad (5)$$

where $I_o$ and I are the integrated emission spectrum with and without quencher. Note: ns emission data using [(*p*–TMA)$Fe^{III}$–Cl] and [(*p*–TMA)$Fe^{II}$–CO] present complications due to the highly absorbing nature of these quenchers. Concentrations using the Stern-Volmer analysis were necessarily low; however, they did span over an order of magnitude, demonstrating the reliability of these results.

# Section 2: Optimization Studies

**Scheme S1:** Previously proposed catalytic cycles relevant to the photochemical evolution of $CH_4$.

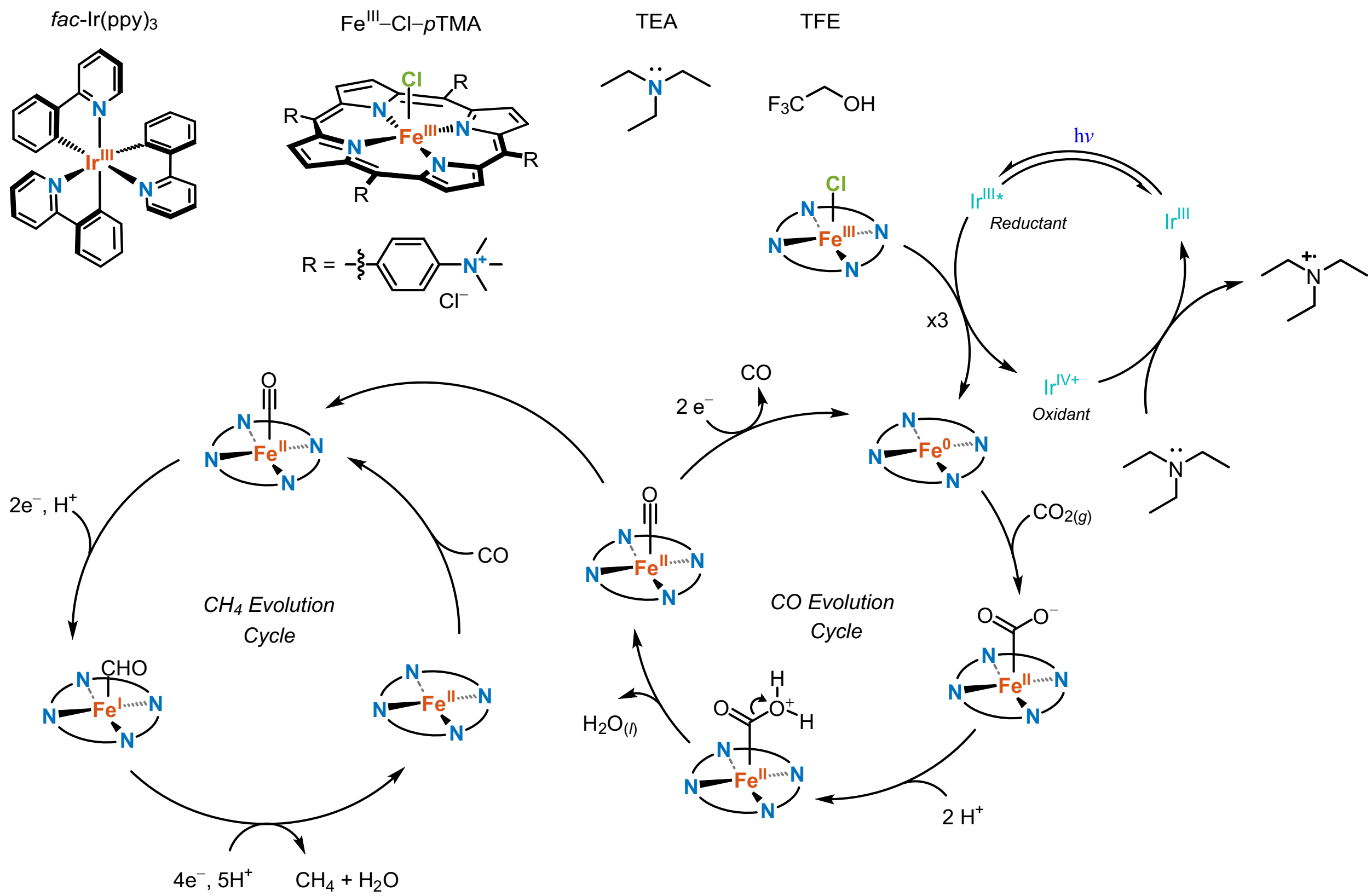

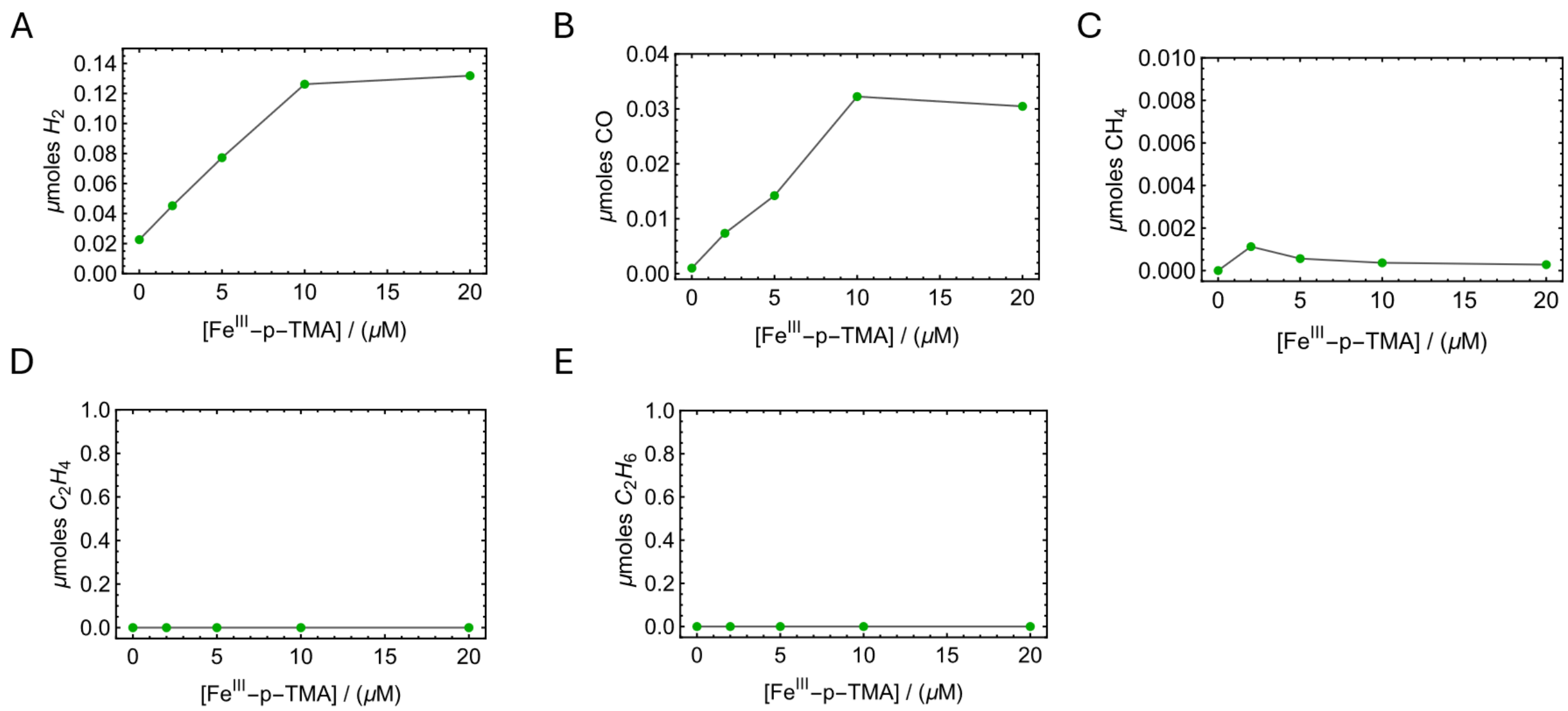


**Figure S1:** Total amount of $H_2$ (**A**), CO (**B**), $CH_4$ (**C**), $C_2H_4$ (**D**), $C_2H_6$ (**E**) as a function of [$Fe^{III}$-p-TMA]. [Ir1] = 0.1 mM, [TEA] = [TFE] = 0.1 M. Solvent: $CH_3CN$, $\lambda_{max}^{irr}$ = 450 nm, 16h, $CO_2$ headspace (4.5 mL), $V_{tot}$: 4 mL.

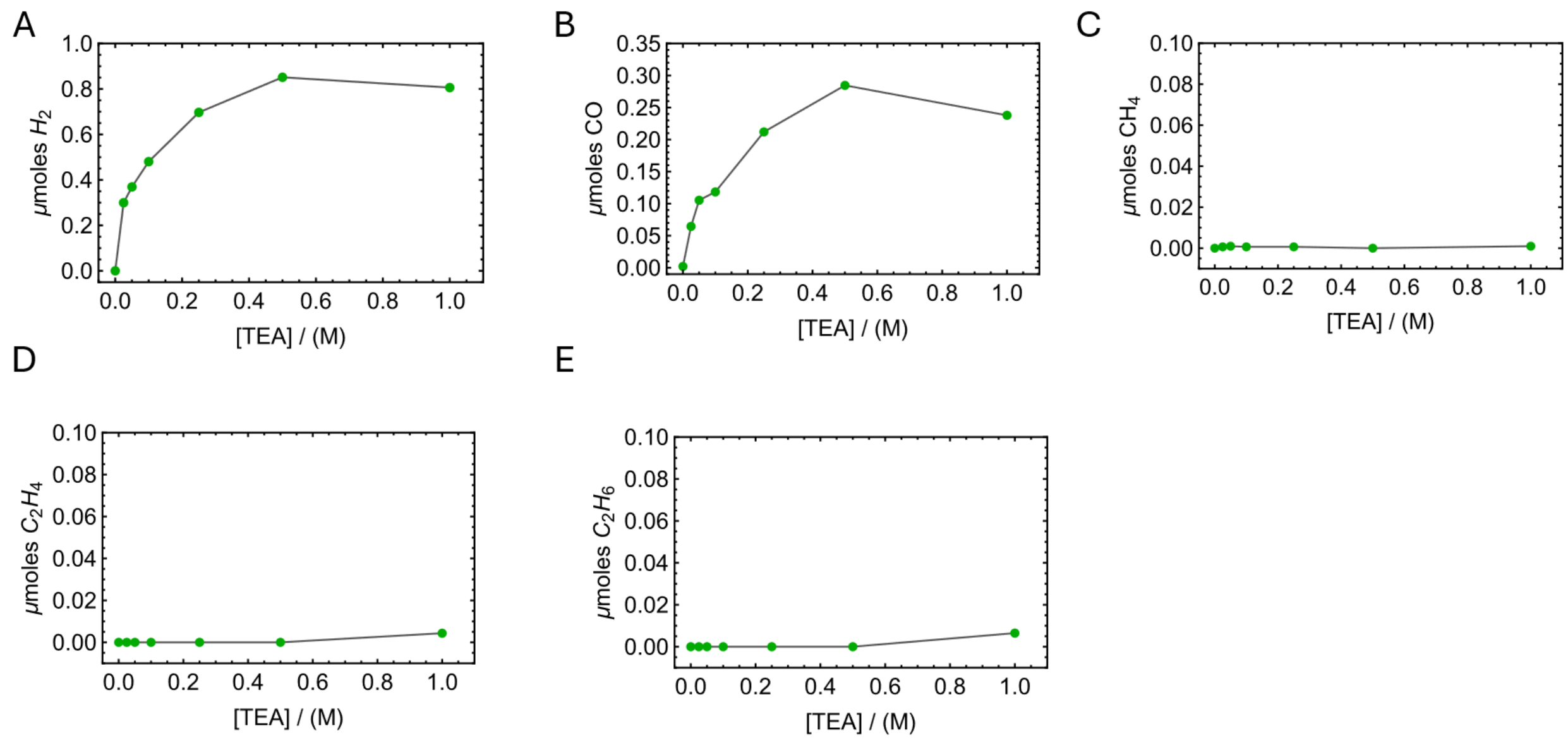


**Figure S2:** Total amount of $H_2$ (**A**), CO (**B**), $CH_4$ (**C**), $C_2H_4$ (**D**), $C_2H_6$ (**E**) as a function of [TEA]. [Ir1] = 0.1 mM, [$Fe^{III}$] = 10 µM, [TFE] = 0.25 M. Solvent: $CH_3CN$, $\lambda_{max}^{irr}$ = 450 nm, 16h, $CO_2$ headspace (4.5 mL), $V_{tot}$: 4 mL.

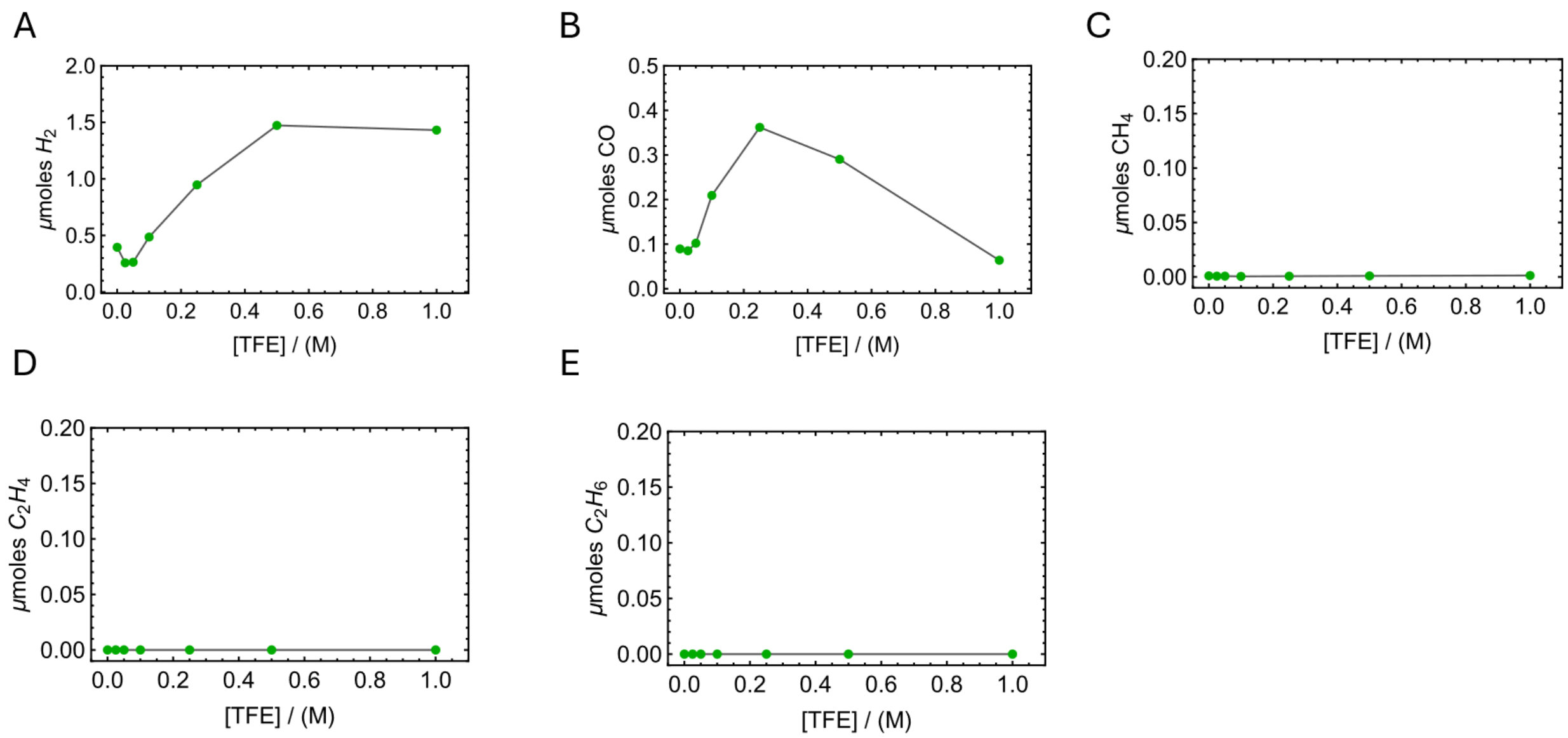


**Figure S3:** Total amount of $H_2$ (**A**), CO (**B**), $CH_4$ (**C**), $C_2H_4$ (**D**), $C_2H_6$ (**E**) as a function of [TFE]. [Ir1] = 0.1 mM, $[Fe^{III}]$ = 10 μM, [TEA] = 0.25 M. Solvent: $CH_3CN$, $\lambda_{max}^{irr}$ = 450 nm, 16h, $CO_2$ headspace (4.5 mL), $V_{tot}$: 4 mL.

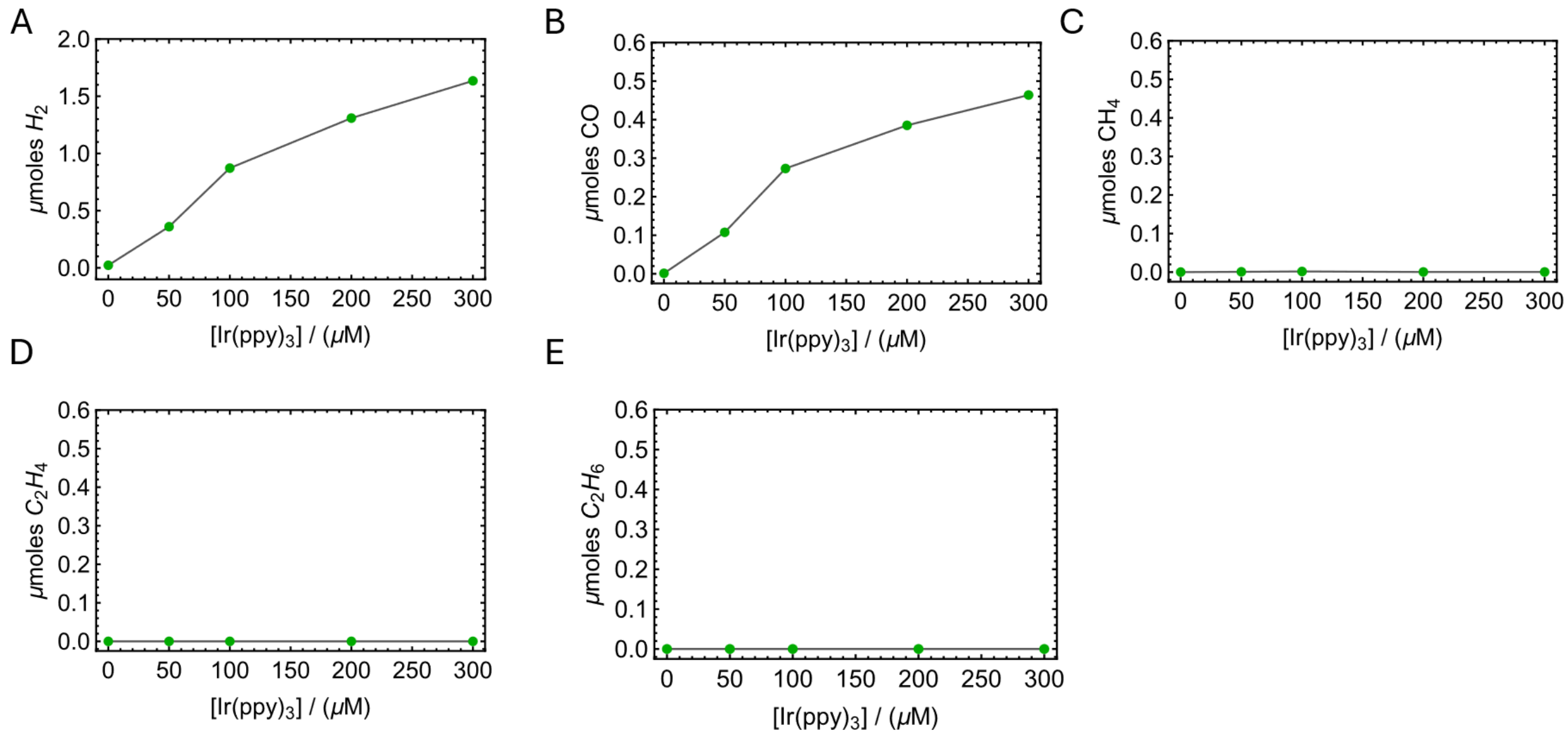


**Figure S4:** Total amount of $H_2$ (**A**), CO (**B**), $CH_4$ (**C**), $C_2H_4$ (**D**), $C_2H_6$ (**E**) as a function of [Ir(ppy)$_3$]. [$Fe^{III}$] = 10 μM, [TEA] = 0.5 M, [TFE] = 0.25 M. Solvent: $CH_3CN$, $\lambda_{max}^{irr}$ = 450 nm, 16h, $CO_2$ headspace (4.5 mL), $V_{tot}$: 4 mL.

**Table S1:** µmoles of product formation under optimized conditions for **Ir1**.

| (µmoles) | $H_2$ | CO | $CH_4$ | $C_2H_4$ | $C_2H_6$ |
|---|---|---|---|---|---|
| Opt. Cond | 1.4 | 0.4 | 0.004 | N/D | N/D |
| No PS | 0.02 | 0.001 | N/D | N/D | N/D |
| No Fe | 0.02 | 0.0008 | N/D | N/D | N/D |
| No TEA | N/D | 0.001 | N/D | N/D | N/D |
| No TFE | 0.4 | 0.8 | 0.001 | N/D | N/D |
| No $CO_2$ | N/D | 0.01 | 0.04 | 0.001 | 0.005 |
| No Light | N/D | N/D | N/D | N/D | N/D |

[Ir1] = 0.1 mM, [$Fe^{III}$] = 10 µM, [TEA] = 0.5 M, [TFE] = 0.25 M. Solvent: $CH_3CN$, $\lambda_{max}^{irr}$ = 450 nm, 16h, $CO_2$ headspace (4.5 mL), $V_{tot}$: 4 mL.

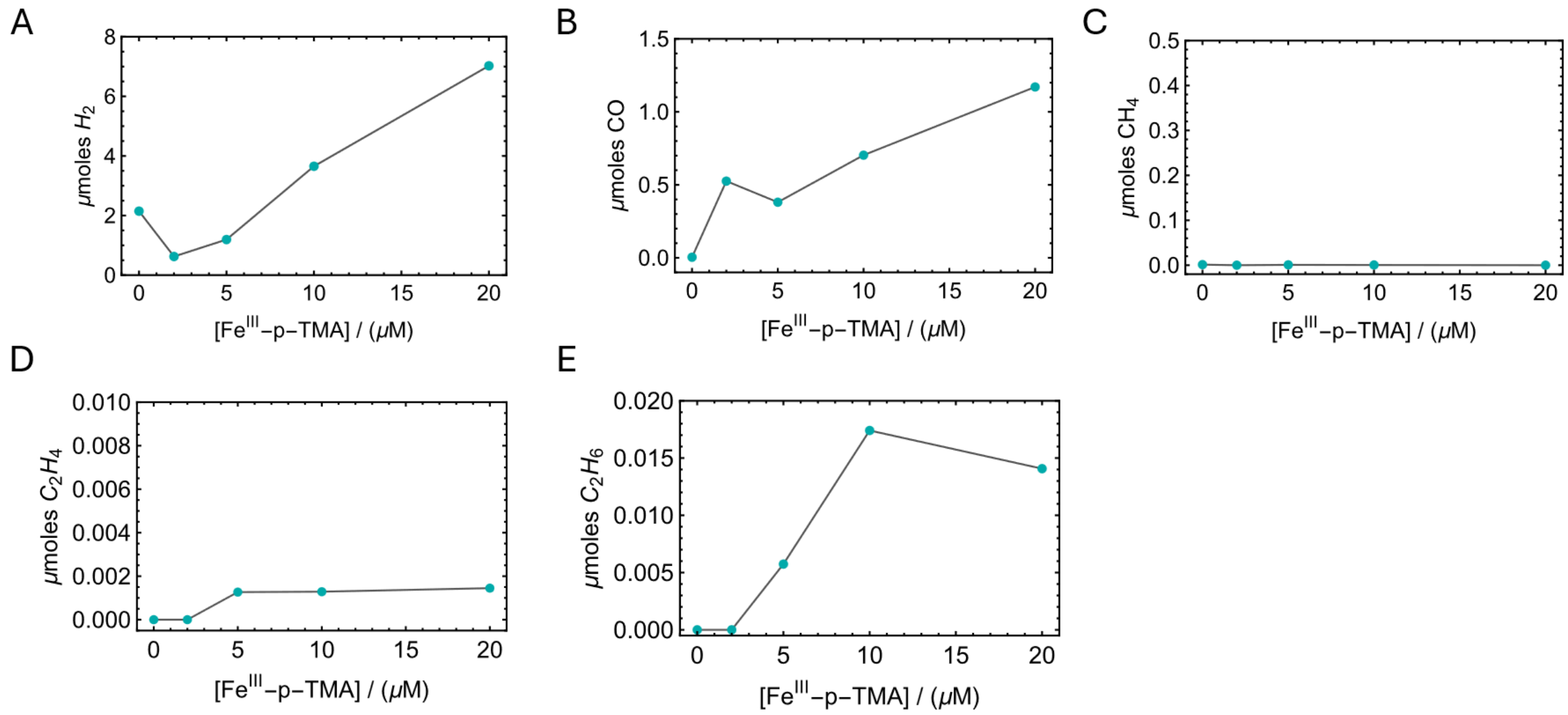


**Figure S5:** Total amount of $H_2$ (**A**), CO (**B**), $CH_4$ (**C**), $C_2H_4$ (**D**), $C_2H_6$ (**E**) as a function of [Fe$^{III}$-p-TMA]. [Ir2] = 0.1 mM, [TEA] = [TFE] = 0.1 M. Solvent: $CH_3CN$, $\lambda_{max}^{irr}$ = 450 nm, 16h, $CO_2$ headspace (4.5 mL), $V_{tot}$: 4 mL.

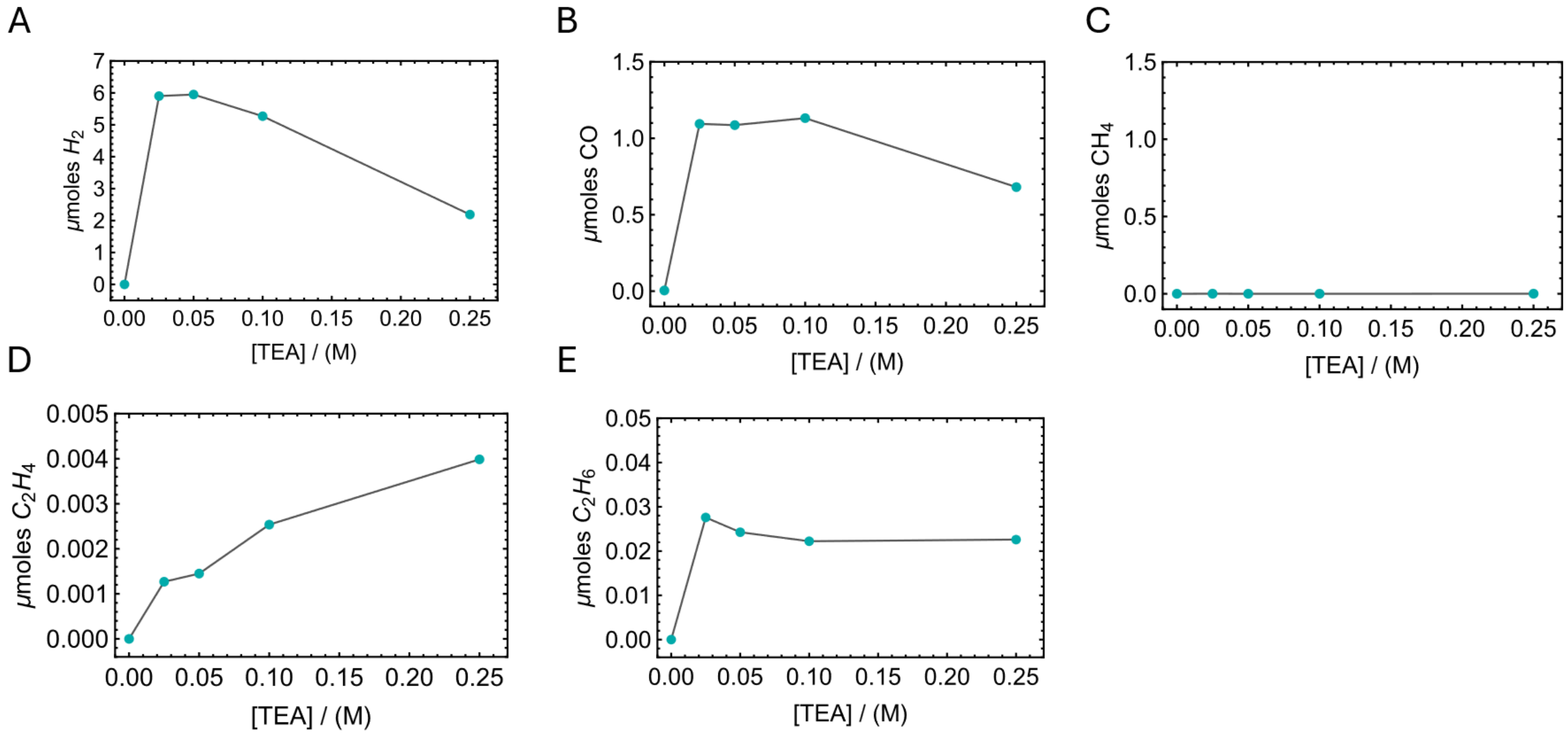


**Figure S6:** Total amount of $H_2$ (**A**), CO (**B**), $CH_4$ (**C**), $C_2H_4$ (**D**), $C_2H_6$ (**E**) as a function of [TEA]. [Ir2] = 0.1 mM, [$Fe^{III}$] = 10 μM, [TFE] = 0.1 M. Solvent: $CH_3CN$, $\lambda_{max}^{irr}$ = 450 nm, 16h, $CO_2$ headspace (4.5 mL), $V_{tot}$: 4 mL.

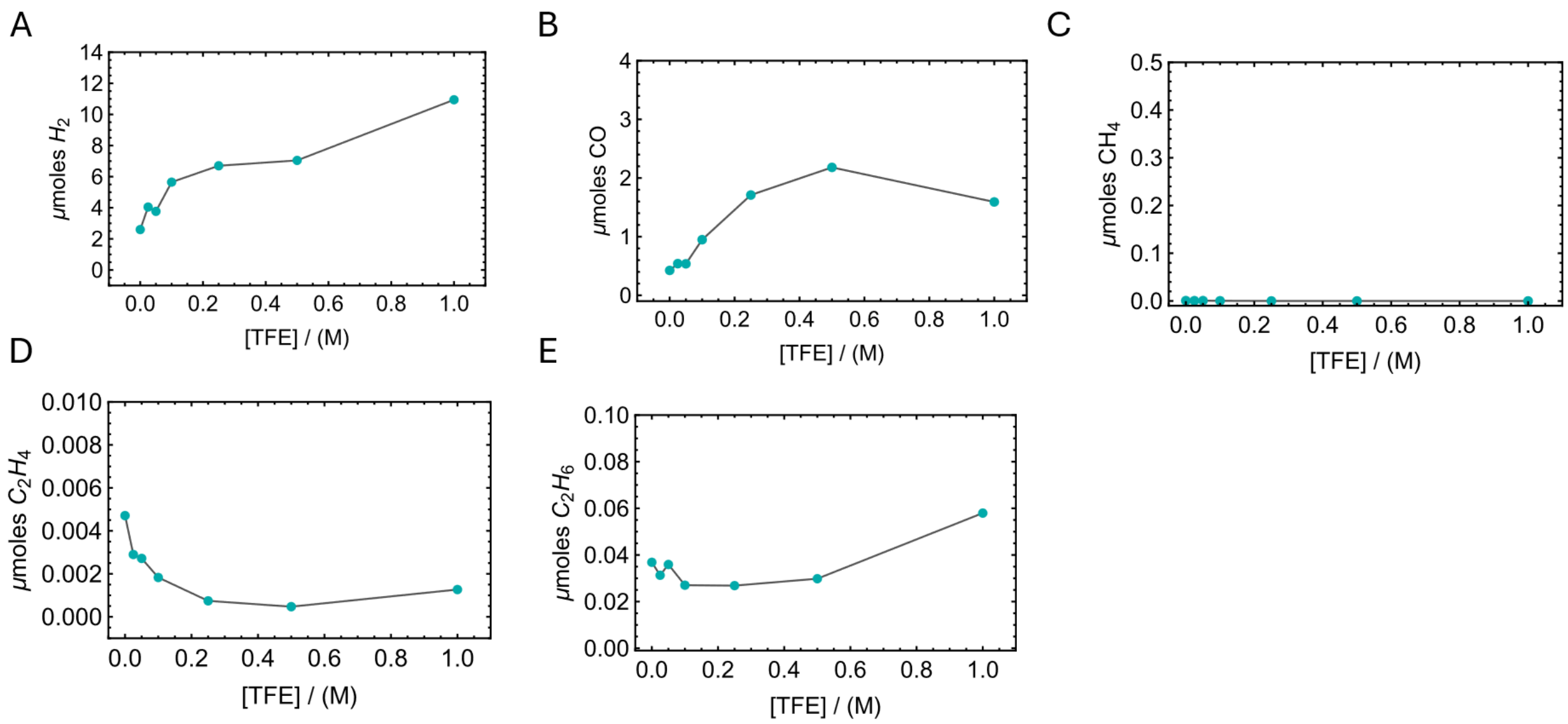


**Figure S7:** Total amount of $H_2$ (**A**), CO (**B**), $CH_4$ (**C**), $C_2H_4$ (**D**), $C_2H_6$ (**E**) as a function of [TFE]. [Ir2] = 0.1 mM, $[Fe^{III}]$ = 10 µM, [TEA] = 0.05 M. Solvent: $CH_3CN$, $\lambda_{max}^{irr}$ = 450 nm, 16h, $CO_2$ headspace (4.5 mL), $V_{tot}$: 4 mL.

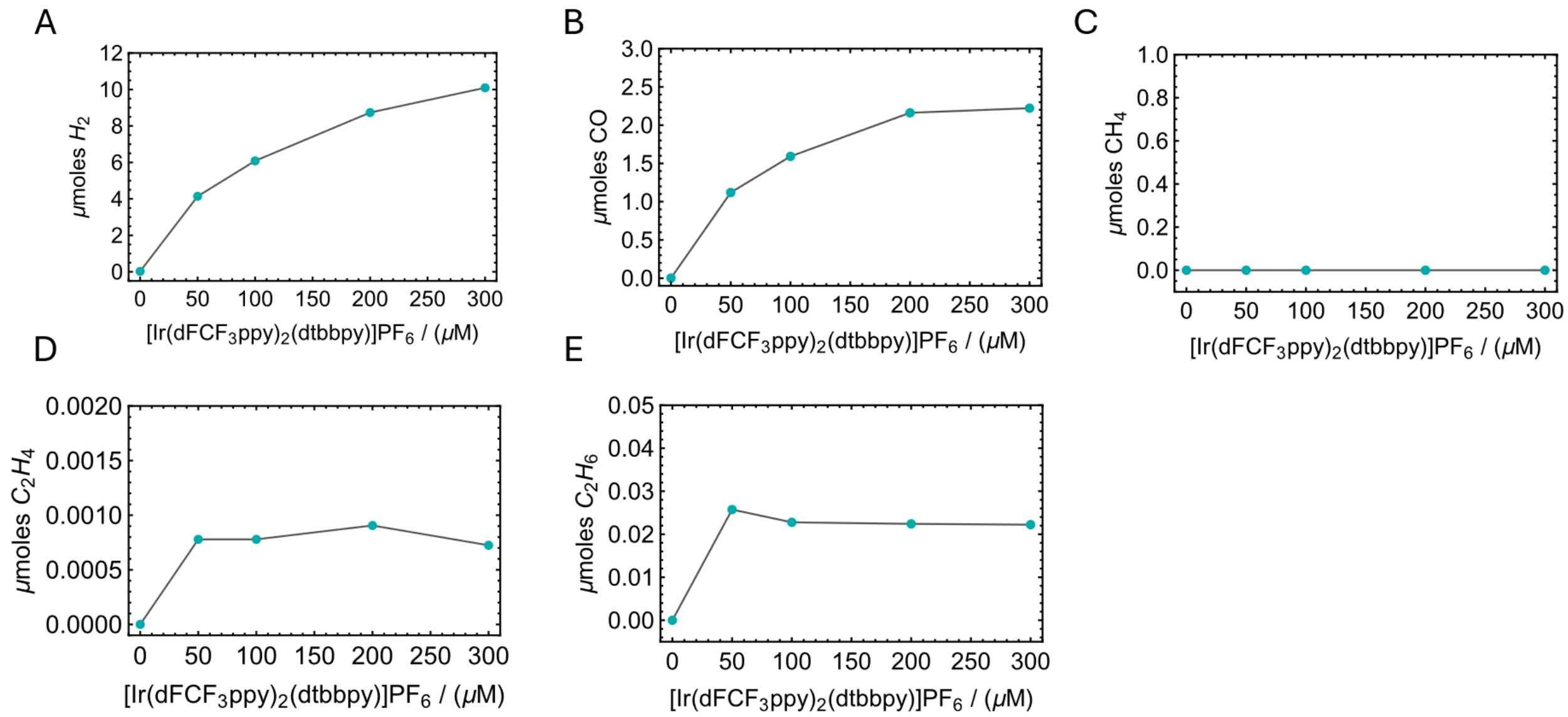


**Figure S8:** Total amount of $H_2$ (**A**), CO (**B**), $CH_4$ (**C**), $C_2H_4$ (**D**), $C_2H_6$ (**E**) as a function of $[Ir(dFCF_3ppy)_2(dtbbpy)]^+PF_6^-$. $[Fe^{III}]$ = 10 μM, [TEA] = 0.05 M, [TFE] = 0.5 M. Solvent: $CH_3CN$, $\lambda_{max}^{irr}$ = 450 nm, 16h, $CO_2$ headspace (4.5 mL), $V_{tot}$: 4 mL.

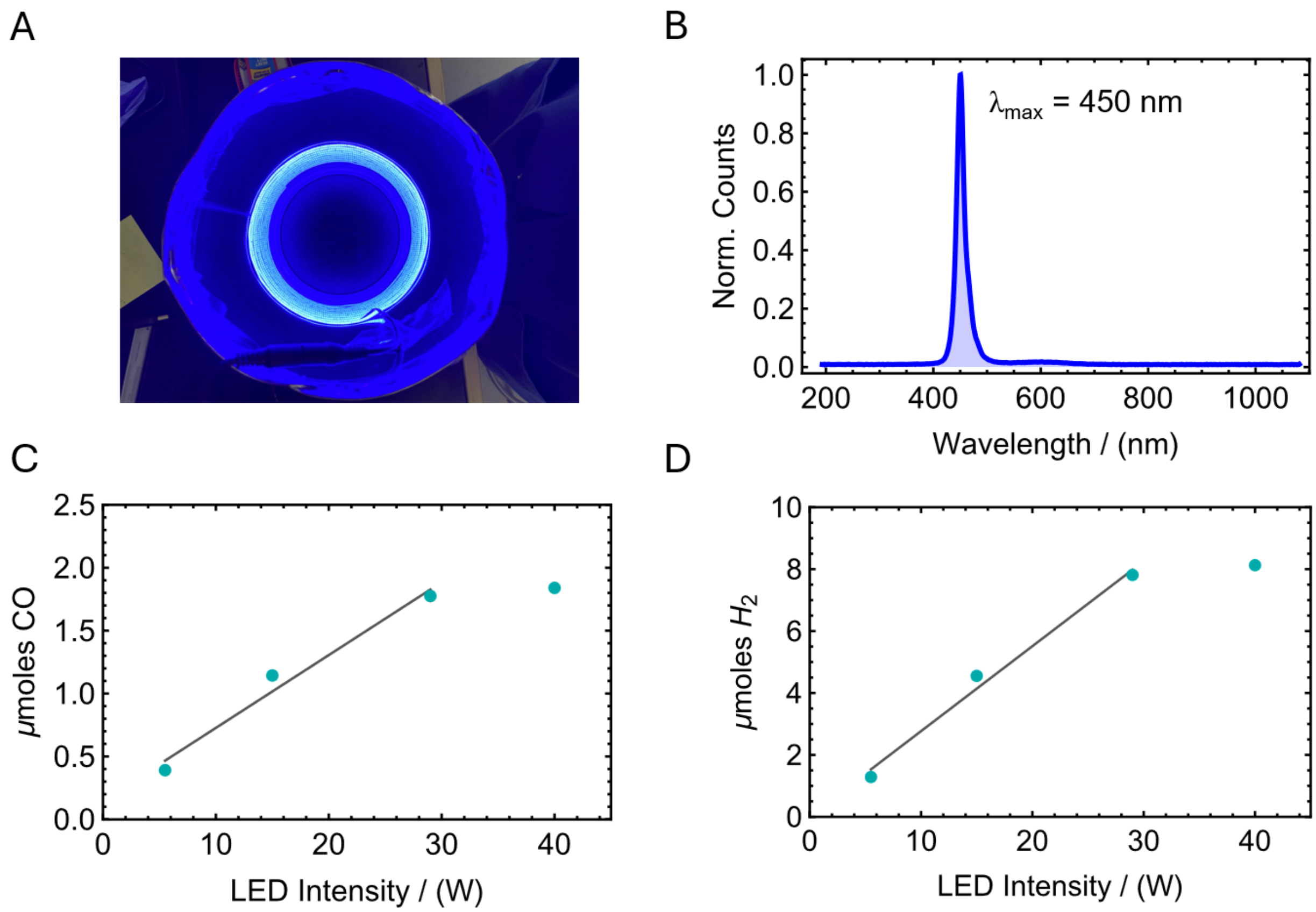


**Figure S9:** (**A**) Photoreactor used to drive the photochemical $CO_2RR$ in this work. (**B**) Emission spectra of the LEDs used to drive the photochemical reaction. Power dependence on $H_2$ (**C**) and CO (**D**) photoevolution. Experiments were conducted at 15 W output power where a linear dependence was observed. [Ir2] = 0.1 mM, [$Fe^{III}$] = 10 μM, [TEA] = 50 mM, [TFE] = 0.5 M. Solvent: $CH_3CN$, $\lambda_{max}^{irr}$ = 450 nm, 30 min, $CO_2$ headspace (4.5 mL), $V_{tot}$: 4 mL.

**Table S2:** µmoles of product formation under optimized conditions for **Ir2**.

| (µmoles) | $H_2$ | CO | $CH_4$ | $C_2H_4$ | $C_2H_6$ |
|---|---|---|---|---|---|
| Opt. Cond | 7.13 | 2.42 | 0.0006 | N/D | 0.03 |
| No PS | 0.02 | 0.001 | N/D | N/D | N/D |
| No Fe | 2.7 | 0.004 | N/D | N/D | N/D |
| FeTPP | 1.14 | 0.35 | N/D | 0.0007 | 0.24 |
| No TEA | N/D | 0.005 | N/D | N/D | N/D |
| No TFE | 2.6 | 0.47 | 0.0006 | N/D | 0.037 |
| No $CO_2$ | N/D | 0.003 | N/D | N/D | N/D |
| No Light | N/D | N/D | N/D | N/D | N/D |

[Ir2] = 0.1 mM, [$Fe^{III}$] = 10 µM, [TEA] = 50 mM, [TFE] = 0.5 M. Solvent: $CH_3CN$, $\lambda_{max}^{irr}$ = 450 nm, 16h, $CO_2$ headspace (4.5 mL), $V_{tot}$: 4 mL.

## *Section 3: GC-MS results and Sacrificial Electron Donor Table*

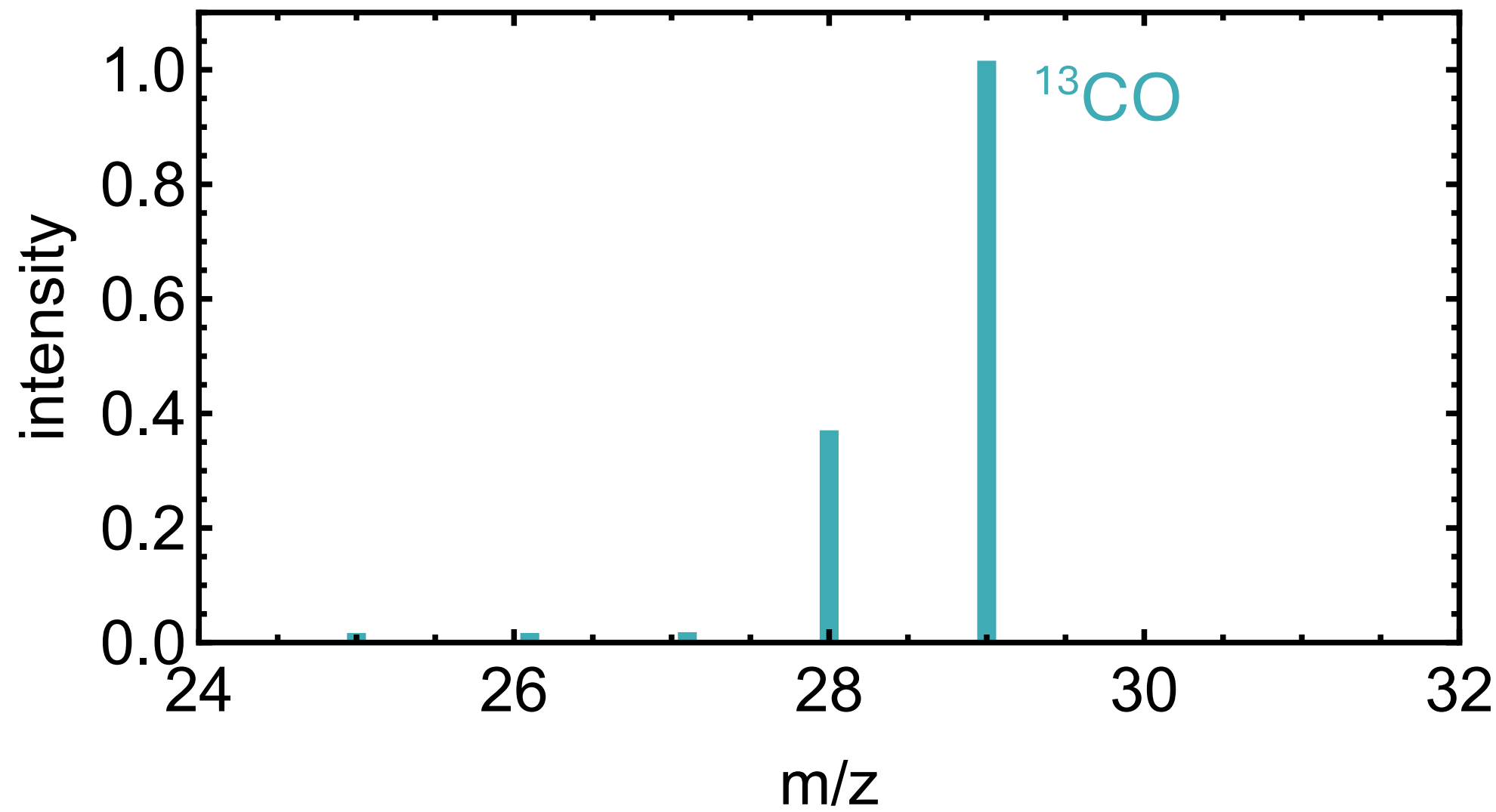


**Figure S10:** GC-MS results for $^{13}CO$ when using $^{13}CO_2$ as the carbon source. [Ir2] = 0.1 mM, [$Fe^{III}$] = 10 µM, [TEA] = 50 mM, [TFE] = 0.5 M. Solvent: $CH_3CN$, $\lambda_{max}^{irr}$ = 450 nm, 16h, $^{13}CO_2$ headspace (4.5 mL), $V_{tot}$: 4 mL.

**Table S3:** Sacrificial electron donors used in this work.

| Acronym | Name | Structure |
|---|---|---|
| TEA | Triethylamine | 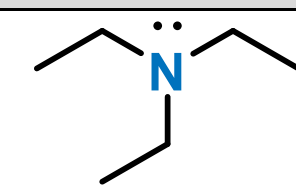 |
| TEOA | Triethanolamine | 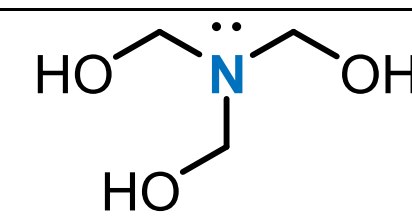 |
| DMA | N,N-Dimethylaniline | 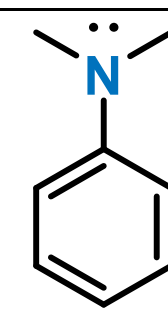 |
| BIH | 2,3-Dihydro-1,3-dimethyl-2-phenylbenzimidazole | 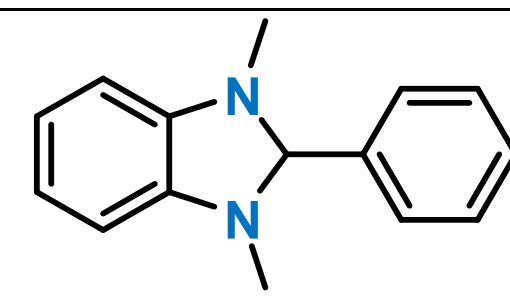 |
| DIPEA | *N*,*N*-Diisopropylethylamine | 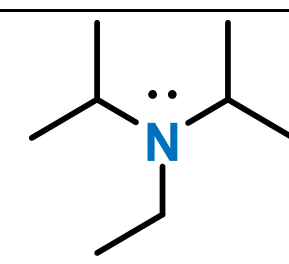 |
| DABCO | 1,4-Diazabicyclo[2.2.2]octane | 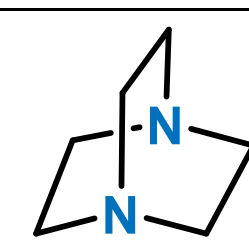 |
| 2TBA$^+$ Ox$^{2-}$ | Tetrabutylammonium Oxalate | 2 $N^+(Bu)_4$ 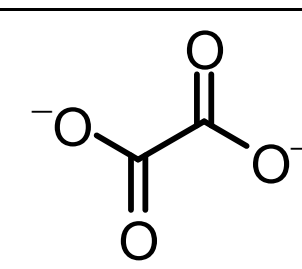 |
| TBA$^+$ Br$^-$ | Tetrabutylammonium Bromide | $N^+(Bu)_4$ $Br^-$ |

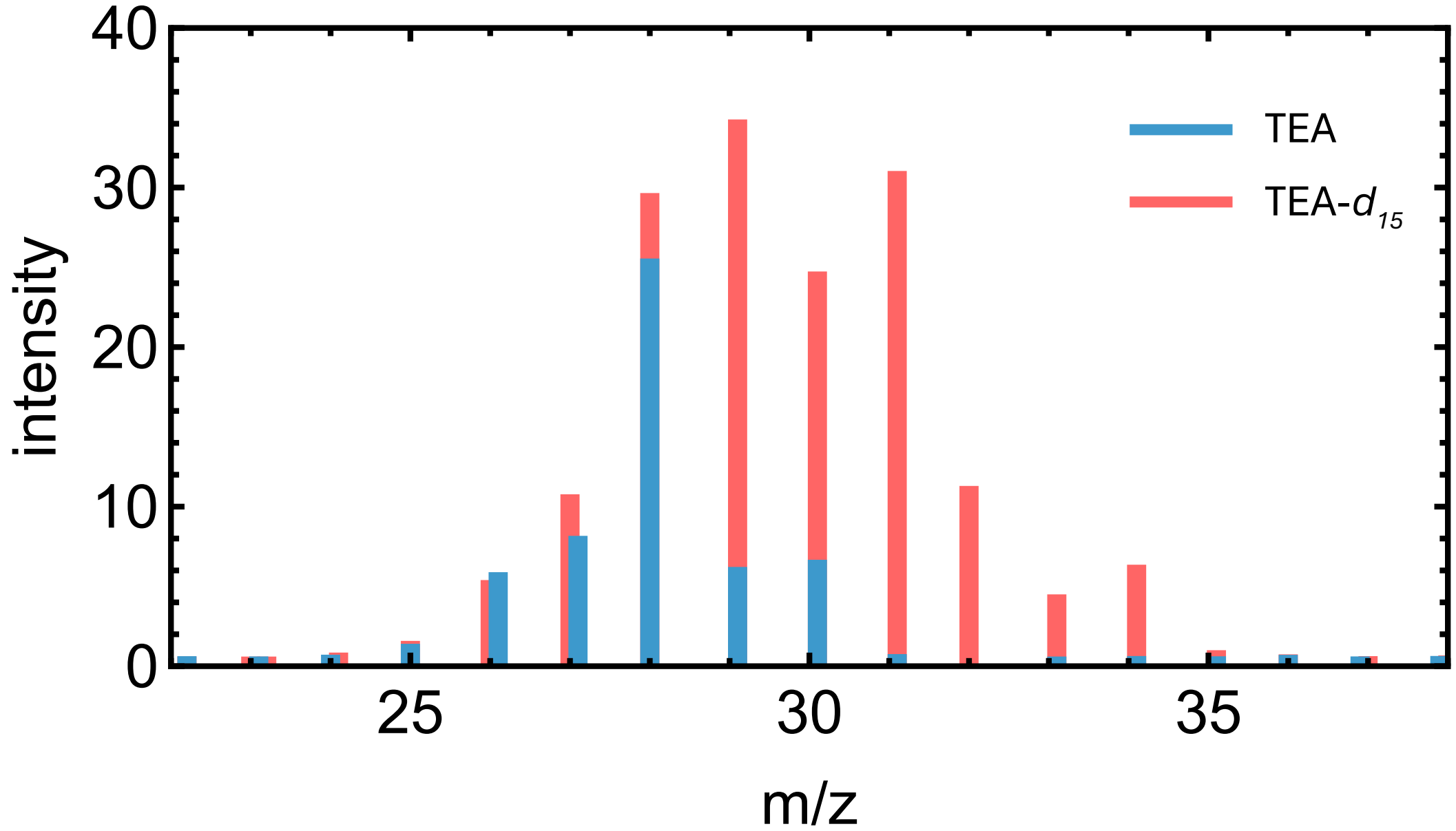


**Figure S11:** GC-MS results for $C_2H_6$ when using TEA-$d_{15}$ as the sacrificial electron donor. [Ir2] = 0.1 mM, [$Fe^{III}$] = 10 µM, [TEA-$d_{15}$] = 50 mM, [TFE] = 0.5 M. Solvent: $CH_3CN$, $\lambda_{max}^{irr}$ = 450 nm, 16h, Ar headspace (4.5 mL), $V_{tot}$: 4 mL.

# *Section 4: Stern-Volmer Analysis using [Fe$^{III}$-p-TMA]*

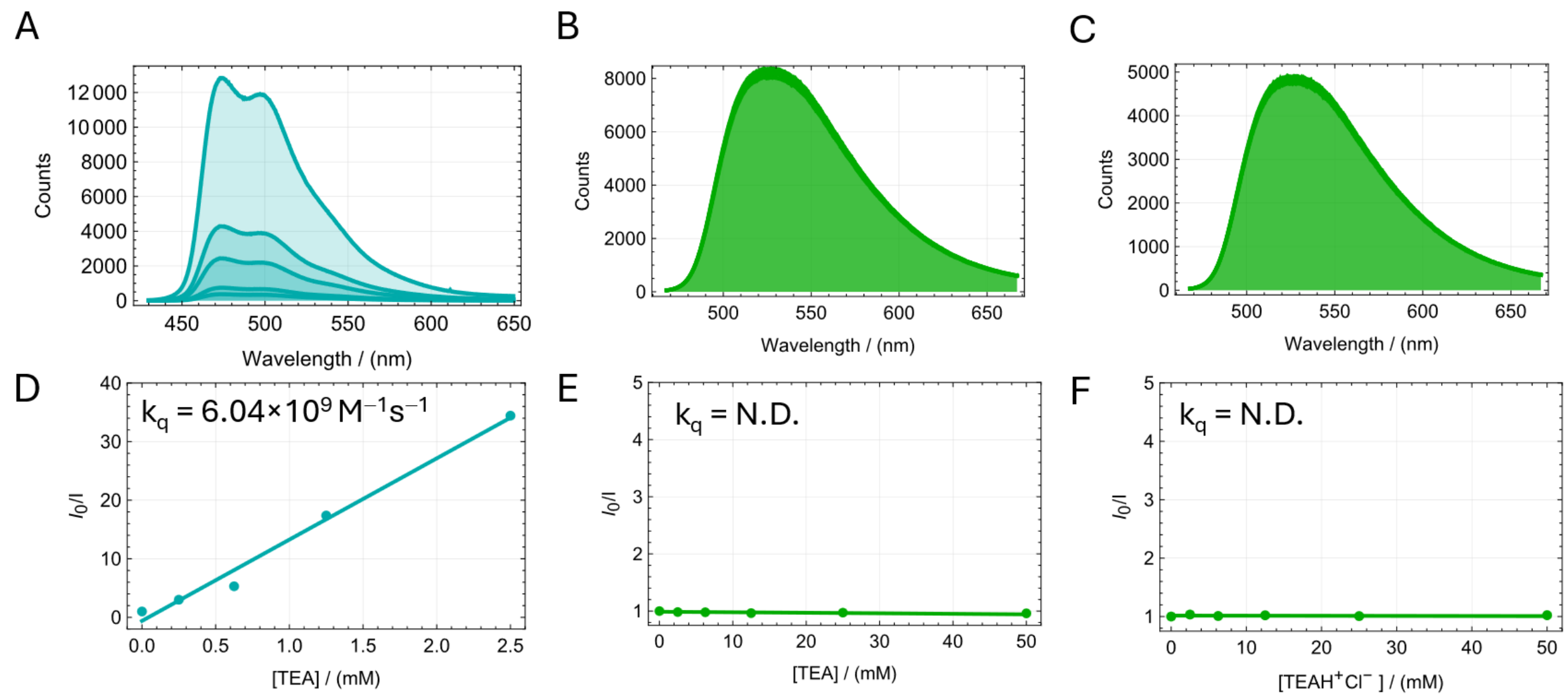


**Figure S12:** Steady-state photoluminescent data of **Ir2** and **Ir1** with various concentrations of TEA (**A**/**B**), respectively. (**C**) Steady-state photoluminescent data of **Ir1** and TEA-$H^+$ $Cl^-$. Associated Stern-Volmer plots between **Ir2** and TEA (**D**), **Ir1** and TEA (**E**), and **Ir1** and TEA-$H^+$ $Cl^-$ (**F**).

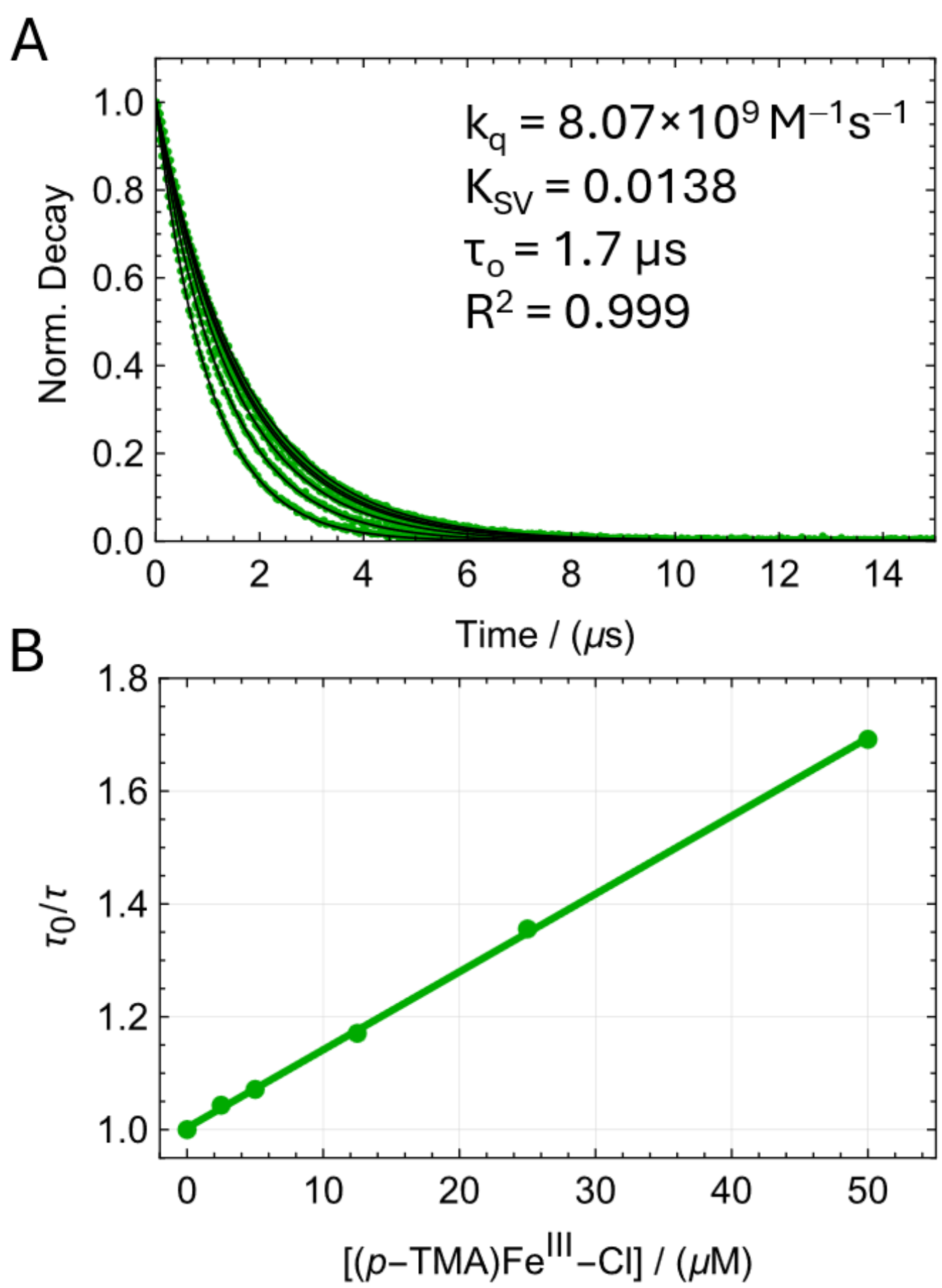


**Figure S13:** Time-resolved photoluminescent decay trace of **Ir1** with various concentration of [(*p*–TMA)$Fe^{III}$–Cl] (**A**) and associated Stern-Volmer analysis (**B**). Fitting parameters for the Stern-Volmer plot are shown in (**A**).

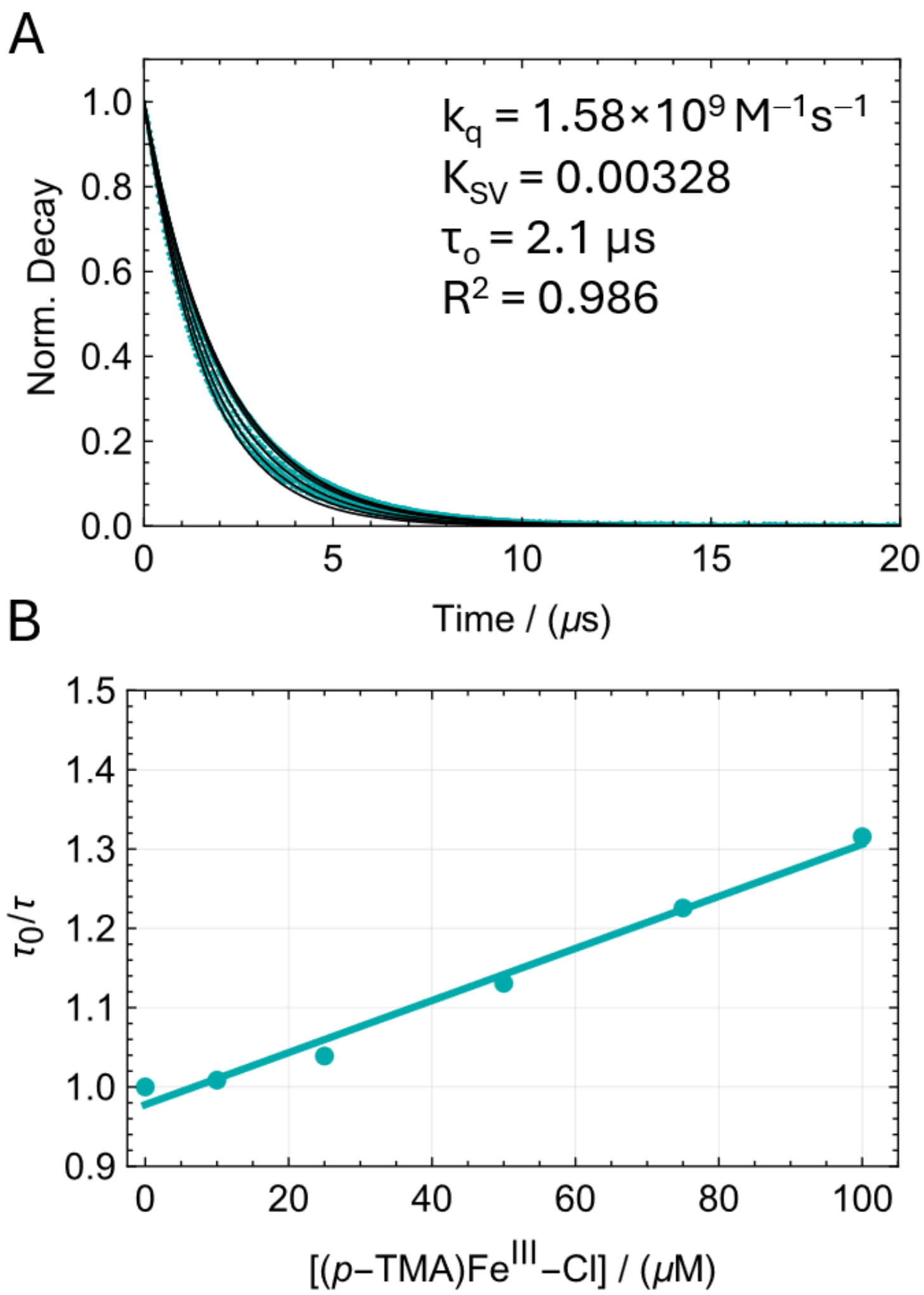


**Figure S14:** Time-resolved photoluminescent decay trace of **Ir2** with various concentration of [(*p*–TMA)$Fe^{III}$–Cl] (**A**) and associated Stern-Volmer analysis (**B**). Fitting parameters for the Stern-Volmer plot are shown in (**A**).

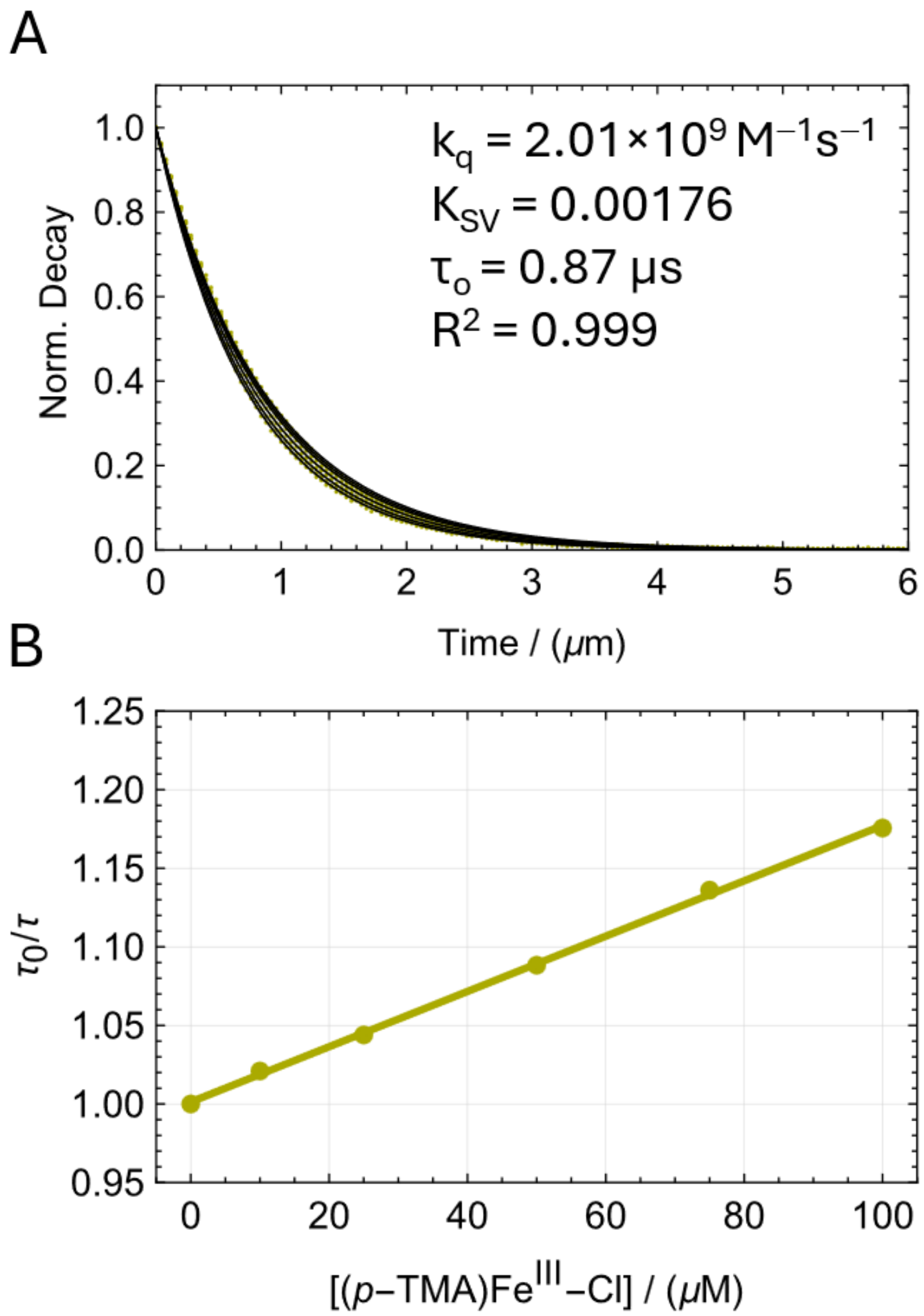


**Figure S15:** Time-resolved photoluminescent decay trace of **Ir3** with various concentration of [(*p*–TMA)$Fe^{III}$–Cl] (**A**) and associated Stern-Volmer analysis (**B**). Fitting parameters for the Stern-Volmer plot are shown in (**A**).

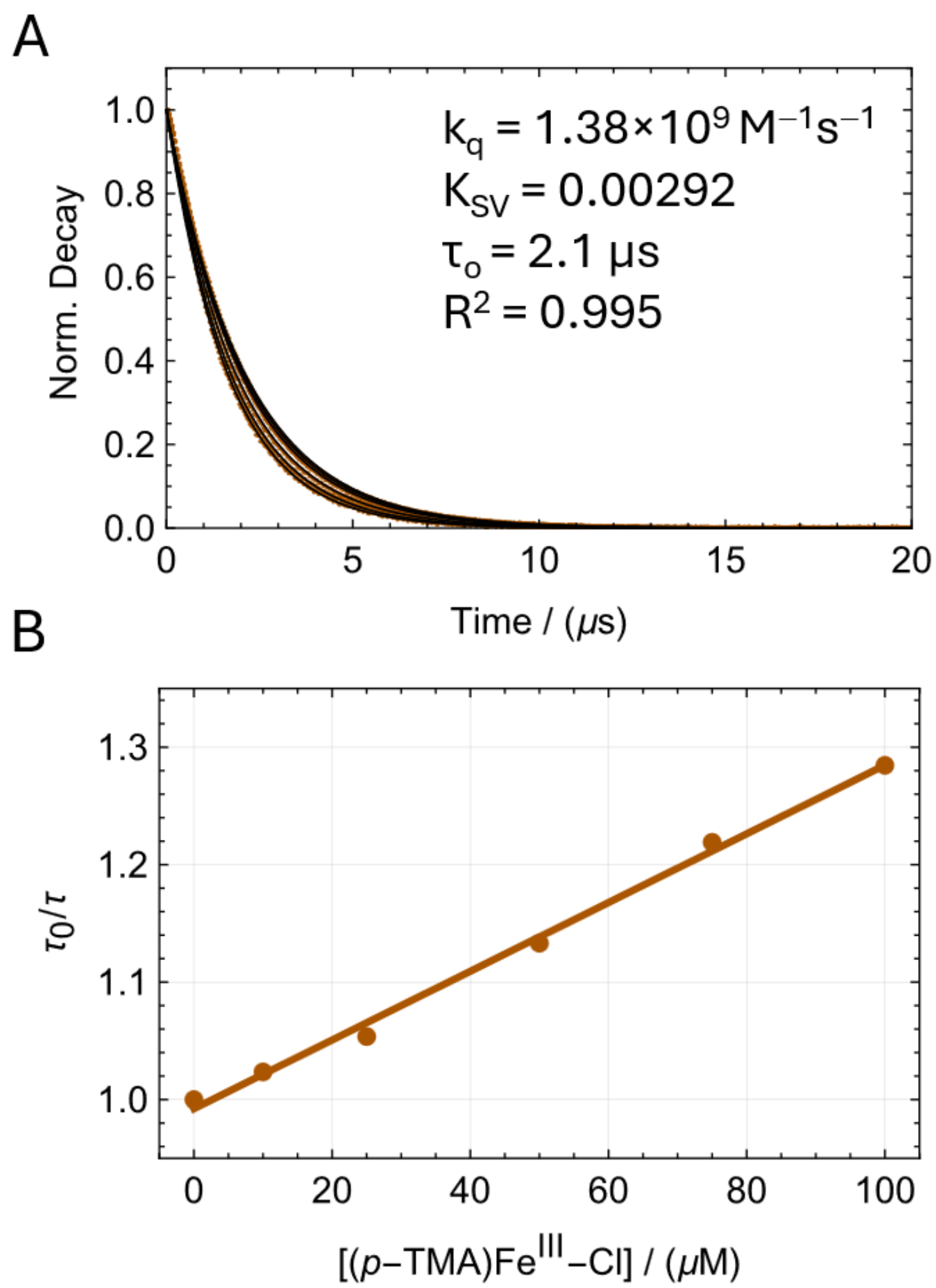


**Figure S16:** Time-resolved photoluminescent decay trace of **Ir4** with various concentration of [(*p*–TMA)$Fe^{III}$–Cl] (**A**) and associated Stern-Volmer analysis (**B**). Fitting parameters for the Stern-Volmer plot are shown in (**A**).

# *Section 5: Cyclic Voltammetry and Spectroelectrochemistry of the PSs*

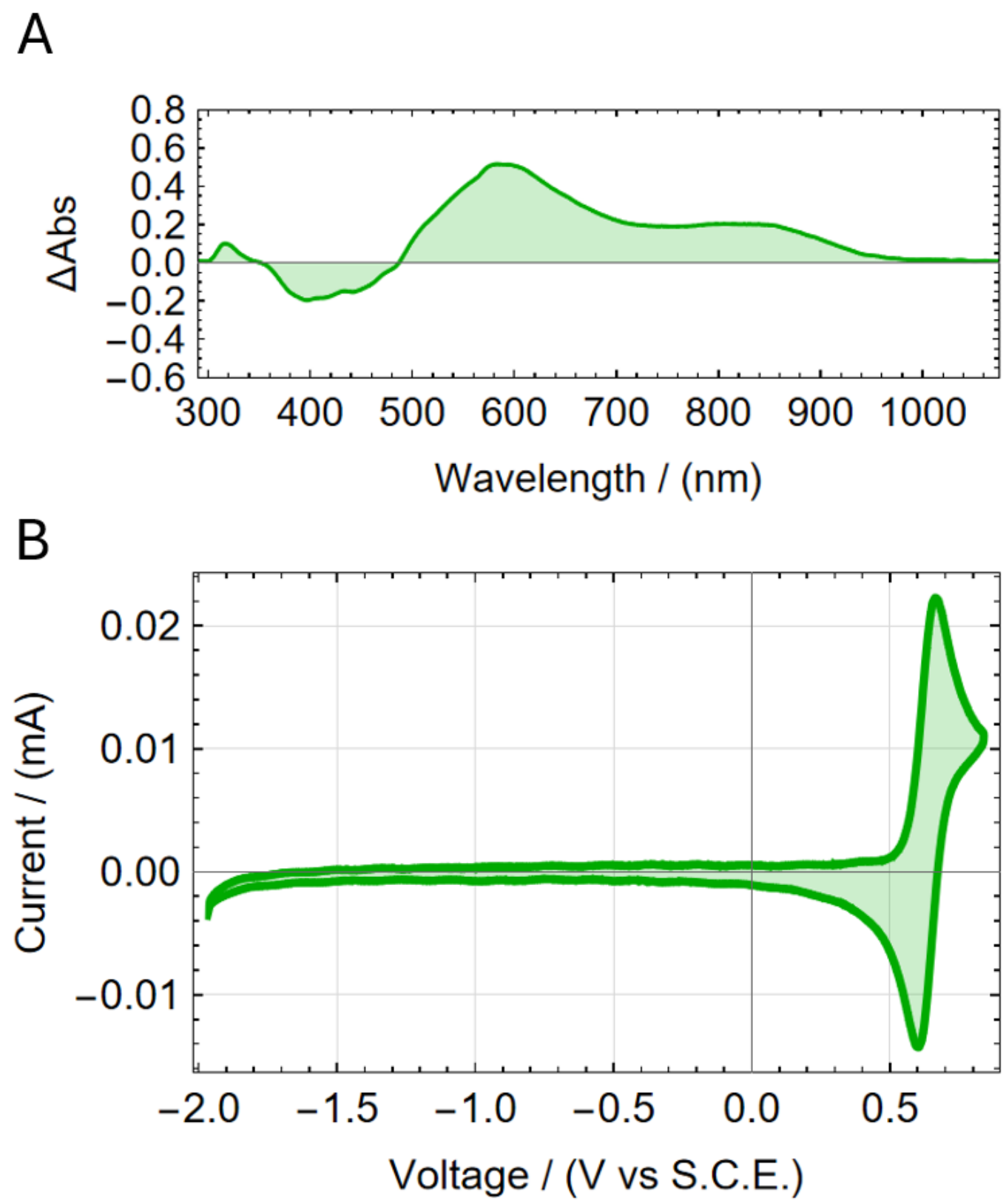


**Figure S17:** (**A**) Change in absorbance of a solution of **Ir1** following application of a +0.75 V vs S.C.E. potential. (**B**) Cyclic voltammogram of **Ir1** measured in deaerated acetonitrile using tetrabutylammonium hexafluorophosphate as a supporting electrolyte. Scan rate: 100 mV/s.

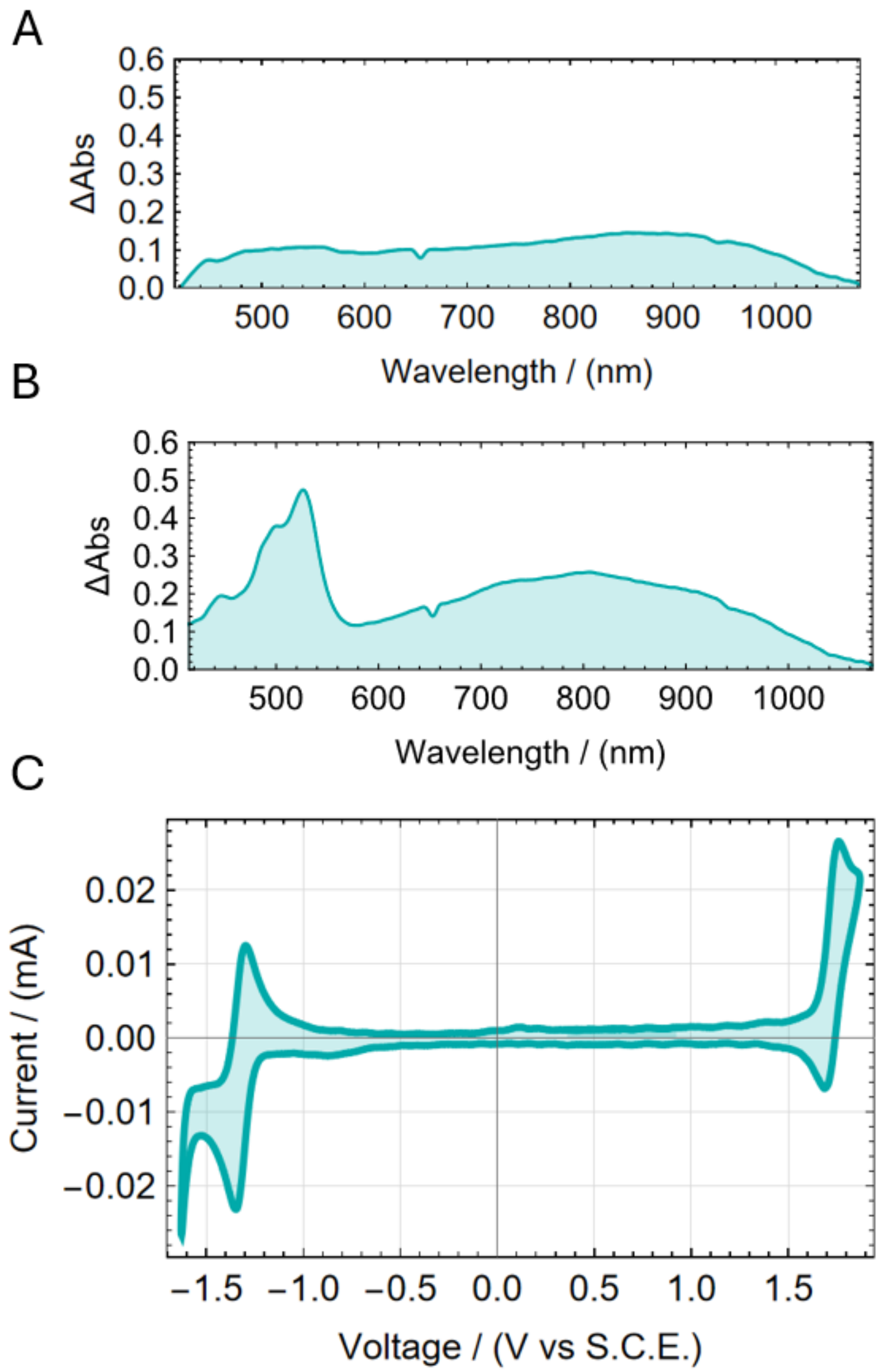


**Figure S18:** Change in absorbance of a solution of **Ir2** following application of a +1.75 V vs S.C.E. (**A**) and −1.5 V vs S.C.E. (**B**) potential. (**C**) Cyclic voltammogram of **Ir2** measured in deaerated acetonitrile using tetrabutylammonium hexafluorophosphate as a supporting electrolyte. Scan rate: 100 mV/s.

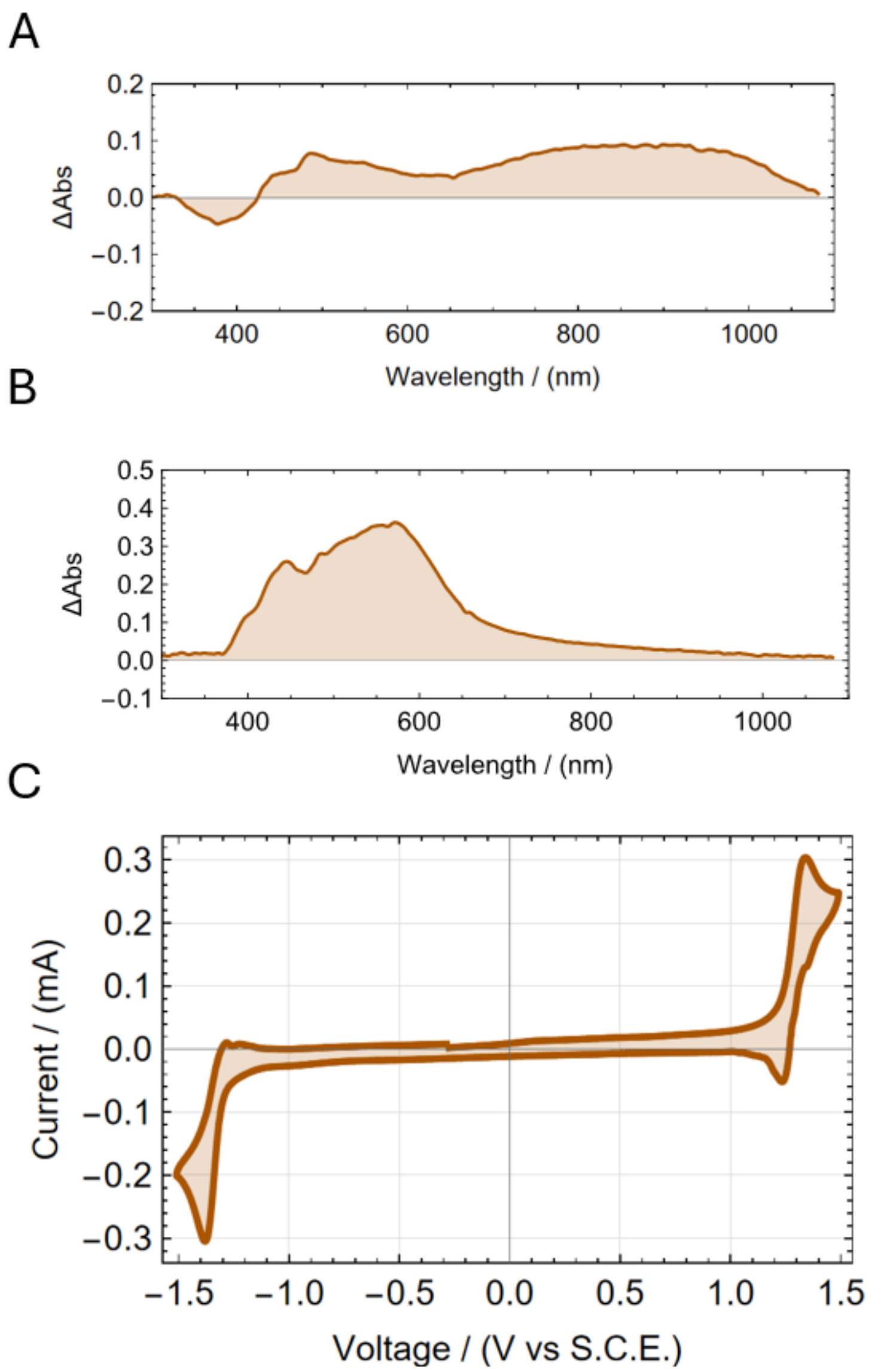


**Figure S19:** Change in absorbance of a solution of **Ir3** following application of a +1.5 V vs S.C.E. (**A**) and −1.5 V vs S.C.E. (**B**) potential. (**C**) Cyclic voltammogram of **Ir3** measured in deaerated acetonitrile using tetrabutylammonium hexafluorophosphate as a supporting electrolyte. Scan rate: 200 mV/s.

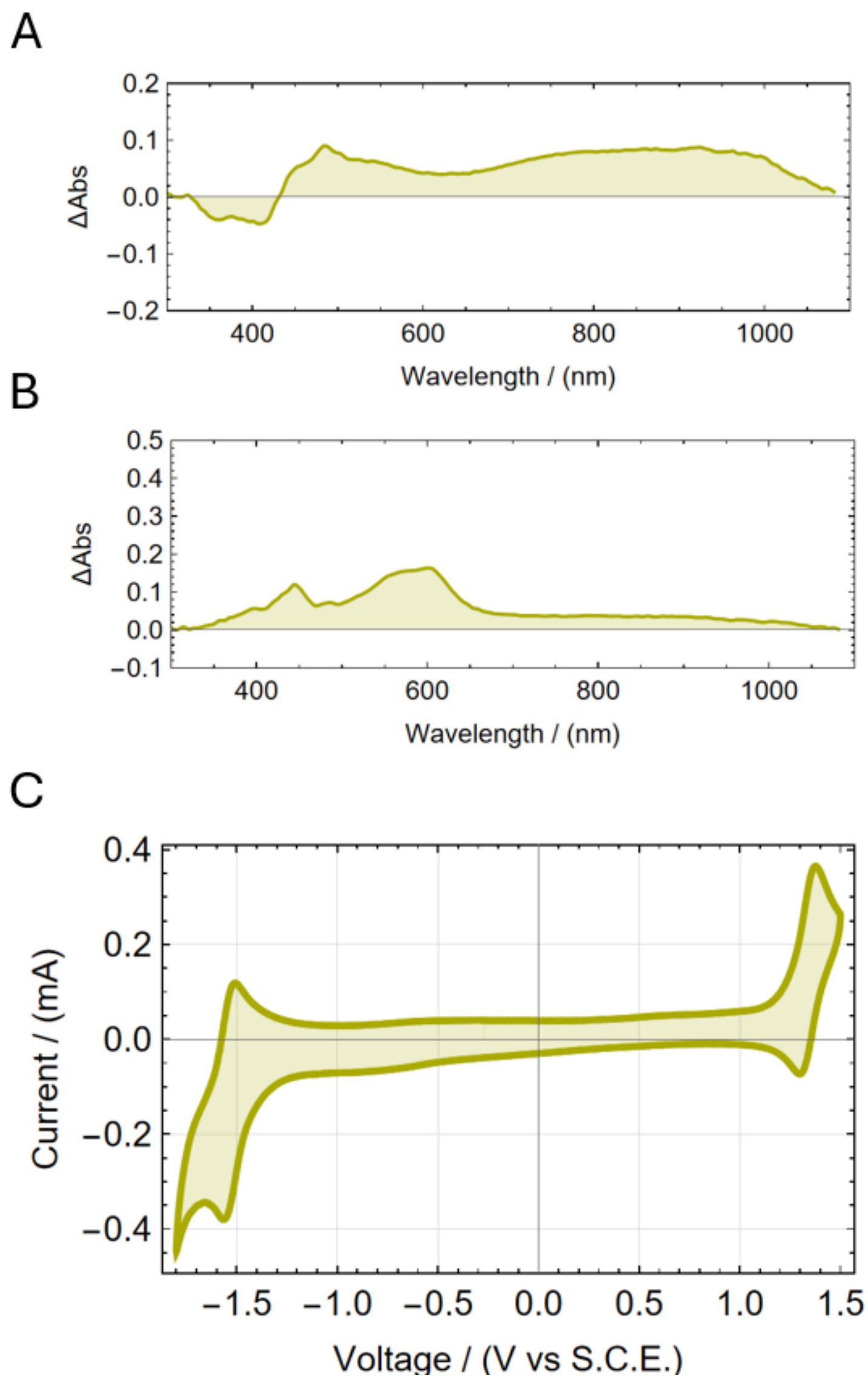


**Figure S20:** Change in absorbance of a solution of **Ir4** following application of a +1.5 V vs S.C.E. (**A**) and −1.7 V vs S.C.E. (**B**) potential. (**C**) Cyclic voltammogram of **Ir4** measured in deaerated acetonitrile using tetrabutylammonium hexafluorophosphate as a supporting electrolyte. Scan rate: 200 mV/s.

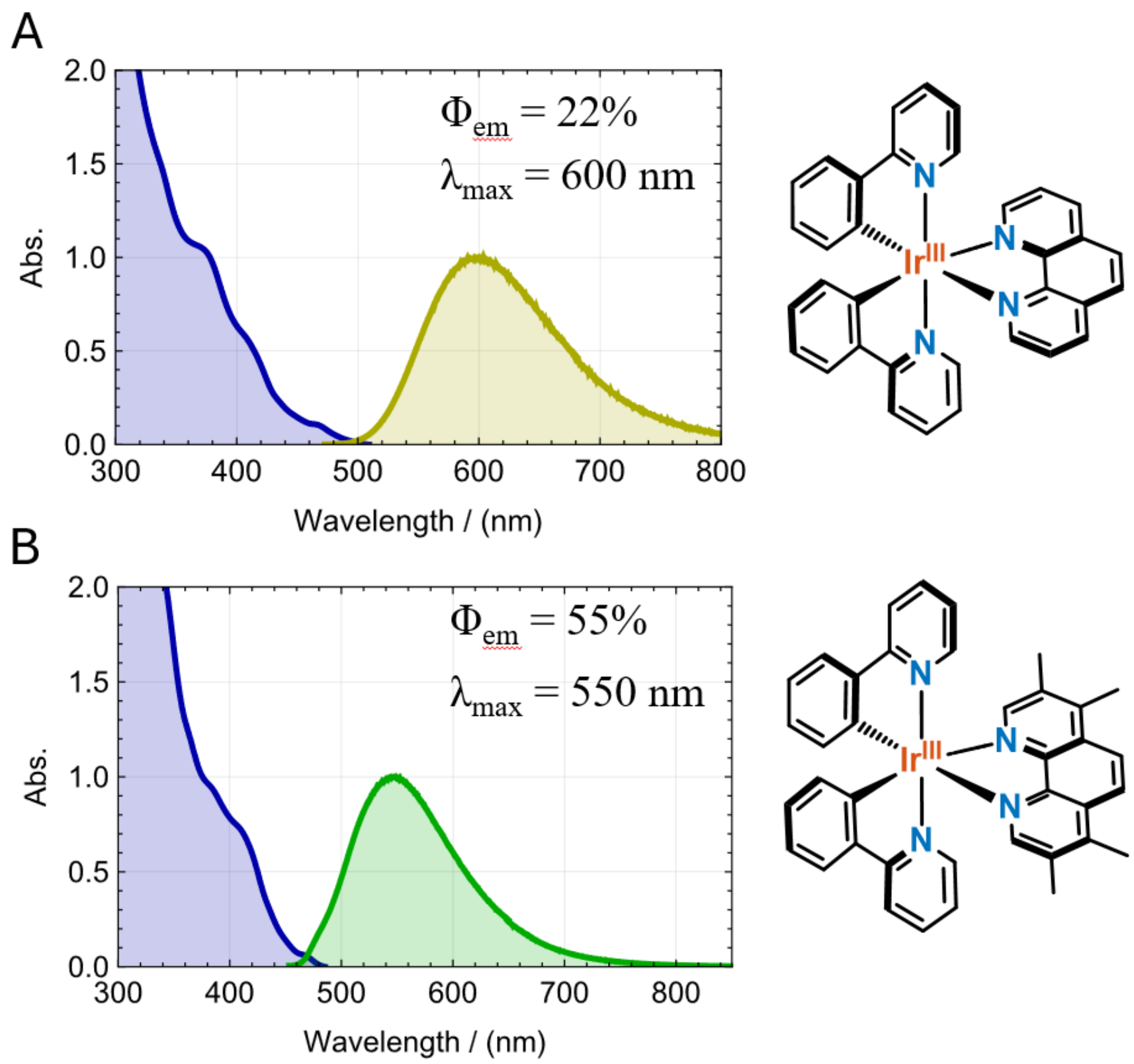


**Figure S21:** Steady-state absorbance and corrected emission spectra of **Ir3** (**A**) and **Ir4** (**B**) measured in aerated and deaerated acetonitrile, respectively. Emission spectra were measured following excitation with 450 nm light. Relative photoluminescent quantum yields are depicted. $k_p$ **Ir3**: 4.1 µs. $k_p$ **Ir4**: 3.8 µs.

**Table S4:** Ground- and excited-state reduction potentials of PSs used in this work. Peak separations are shown in parenthesis for the ground-state electrochemical potentials. All potentials are referenced to the S.C.E.

| PS | $Ir^{+/0}$ | $^{1}Ir^{2+/+}$ | $Em_{max}$ (eV) | $Ir^{*/-}$ | $Ir^{+/*}$ | ${}^{b}k_q \times 10^9$ ($M^{-1}s^{-1}$) |
|---|---|---|---|---|---|---|
| [5]**Ir1** | N/A | +0.63 (*60*) | 2.36 | +0.33 | −1.73 | 8.07 |
| [6]**Ir2** | −1.33 (*50*) | +1.72 (*80*) | 2.64 | +1.21 | −0.89 | 1.58 |
| [7]**Ir3** | −1.34 (*100*) | +1.29 (*110*) | 2.07 | +1.08 (+0.73)[a] | −0.78[a] | 2.01 |
| **Ir4** | −1.53 (*60*) | +1.35 (*80*) | 2.26 | +0.73[a] | −0.91[a] | 1.26 |

[a]Calculated from the emission maximum of the room-temperature photoluminescence spectra. [b]Bimolecular quenching rate constant measured by Stern-Volmer analysis for $[(p\text{–TMA})Fe^{III}\text{–Cl}]^{4+}$.

## *Section 6: Transient Absorption Spectroscopy*

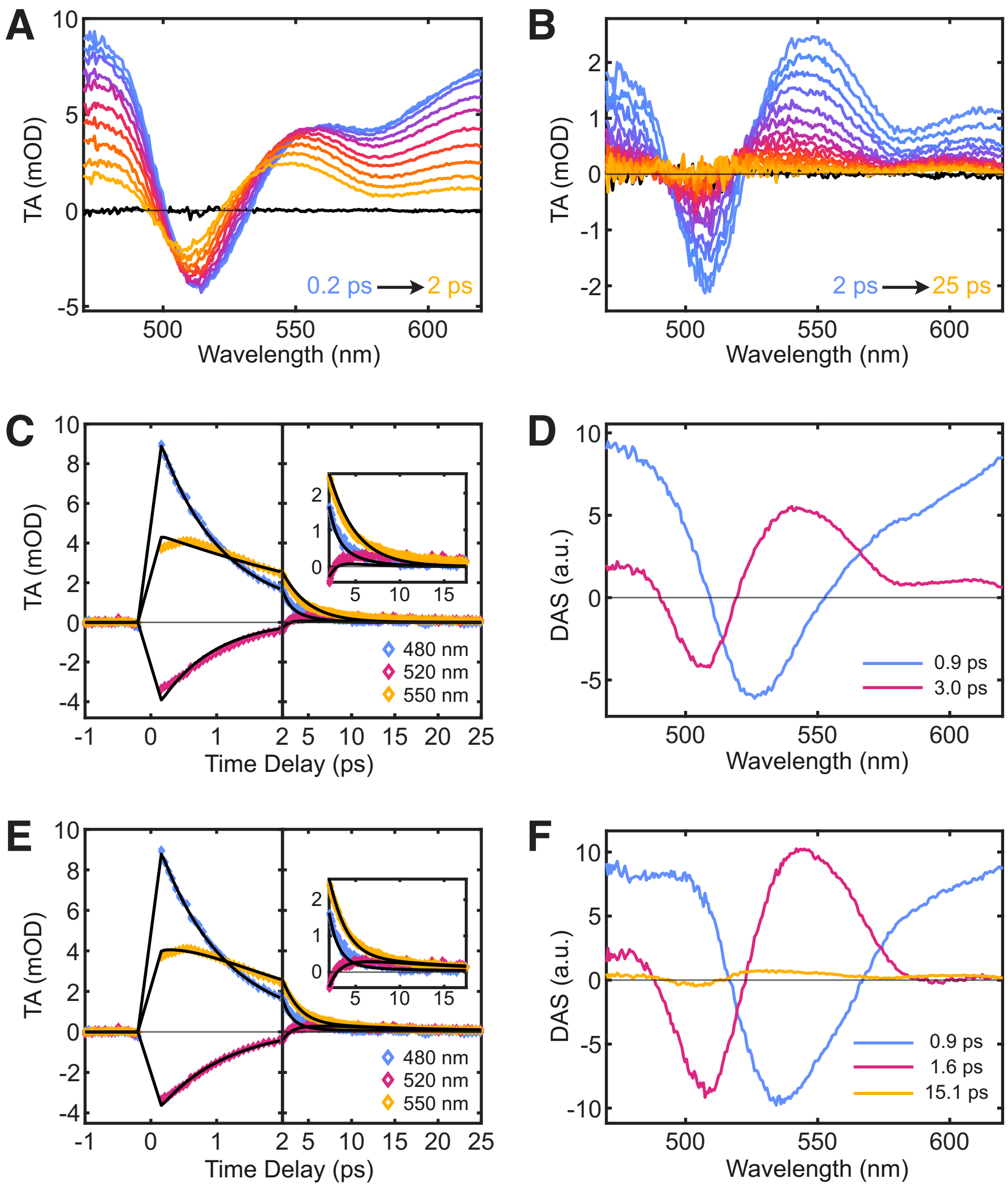


**Figure S22:** TA spectra of [(*p*–TMA)$Fe^{III}$–Cl] using 680 nm, 0.5 μJ, depolarized pump pulses. (**A**, **B**) Difference spectra for shorter and longer time regions. Multiexponential global fits to the data with (**C**, **D**) 2 and (**E**, **F**) 3 exponentials are presented. (**C**, **E**) Fits plotted alongside kinetic traces at various wavelengths. Inset shows a zoomed in view of the data at longer times. (**D**, **F**) Decay associated spectra (DAS) for the fits. Time constants corresponding to each DAS are given in the legend. Best fit achieved with three components: 0.9, 1.6, 15.1 ps.

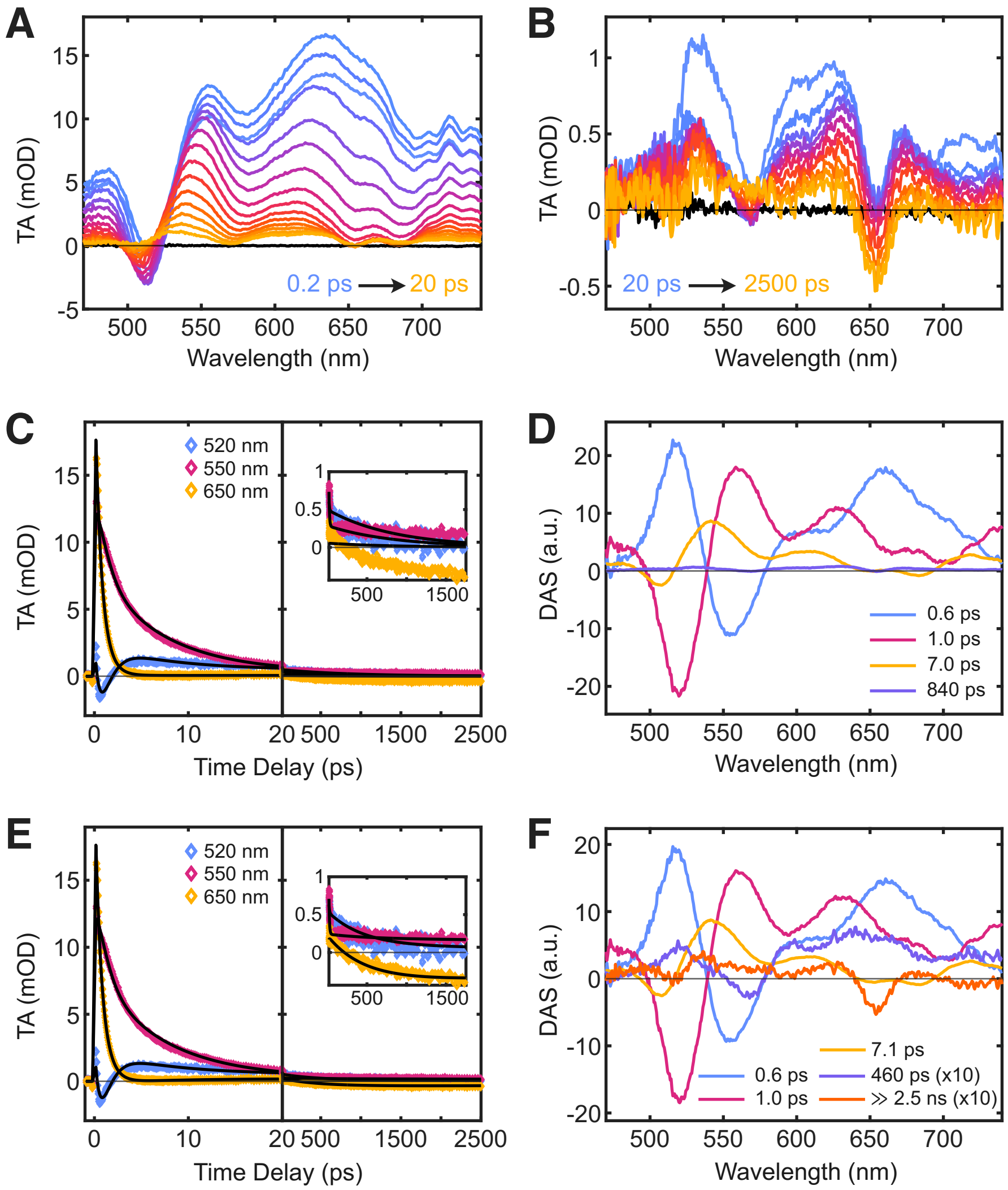


**Figure S23:** TA spectra of [(*p*–TMA)$Fe^{III}$–Cl] using 400 nm, 0.5 μJ, depolarized pump pulses. (**A**, **B**) Difference spectra for shorter and longer time regions. Multiexponential global fits to the data with (**C**, **D**) 4 and (**E**, **F**) 5 exponentials are presented. (**C**, **E**) Fits plotted alongside kinetic traces at various wavelengths. Inset shows a zoomed in view of the data at longer times. (**D**, **F**) Decay associated spectra (DAS) for the fits. Time constants corresponding to each DAS are given in the legend. Best fit achieved with five components: 0.6, 1.0, 7.1, 460, >2500 ps, where the latter is just a lower bound.

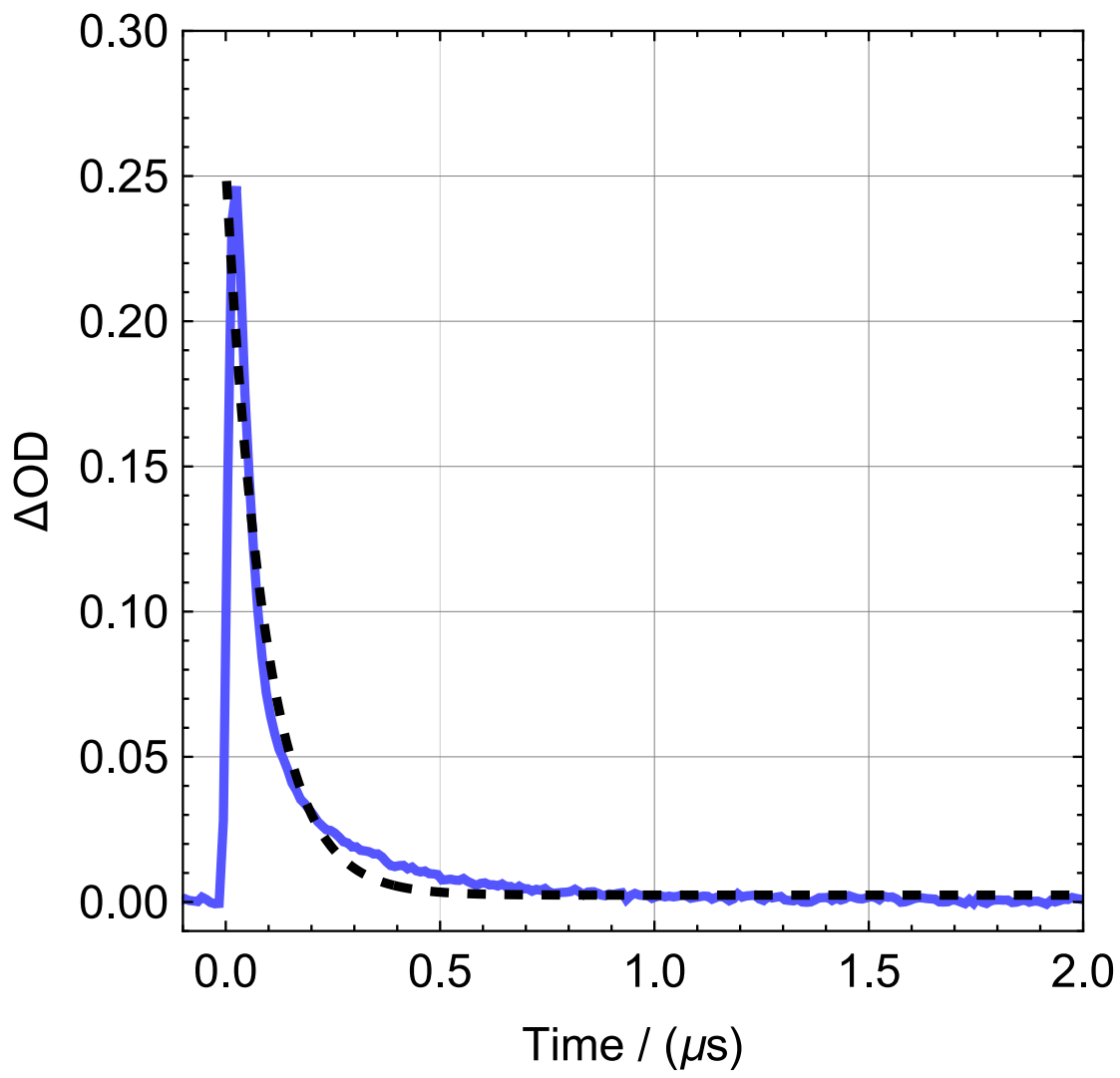


**Figure S24:** Nanosecond TA signal at $\lambda_{probe}$ = 800 nm of [(*p*–TMA)$Fe^{III}$–Cl] following a $\lambda_{pump}$ = 450 nm excitation pulse. Black dashed line represents the best fit single exponential decay curve, demonstrating a non-unimolecular first order decay process following excitation.

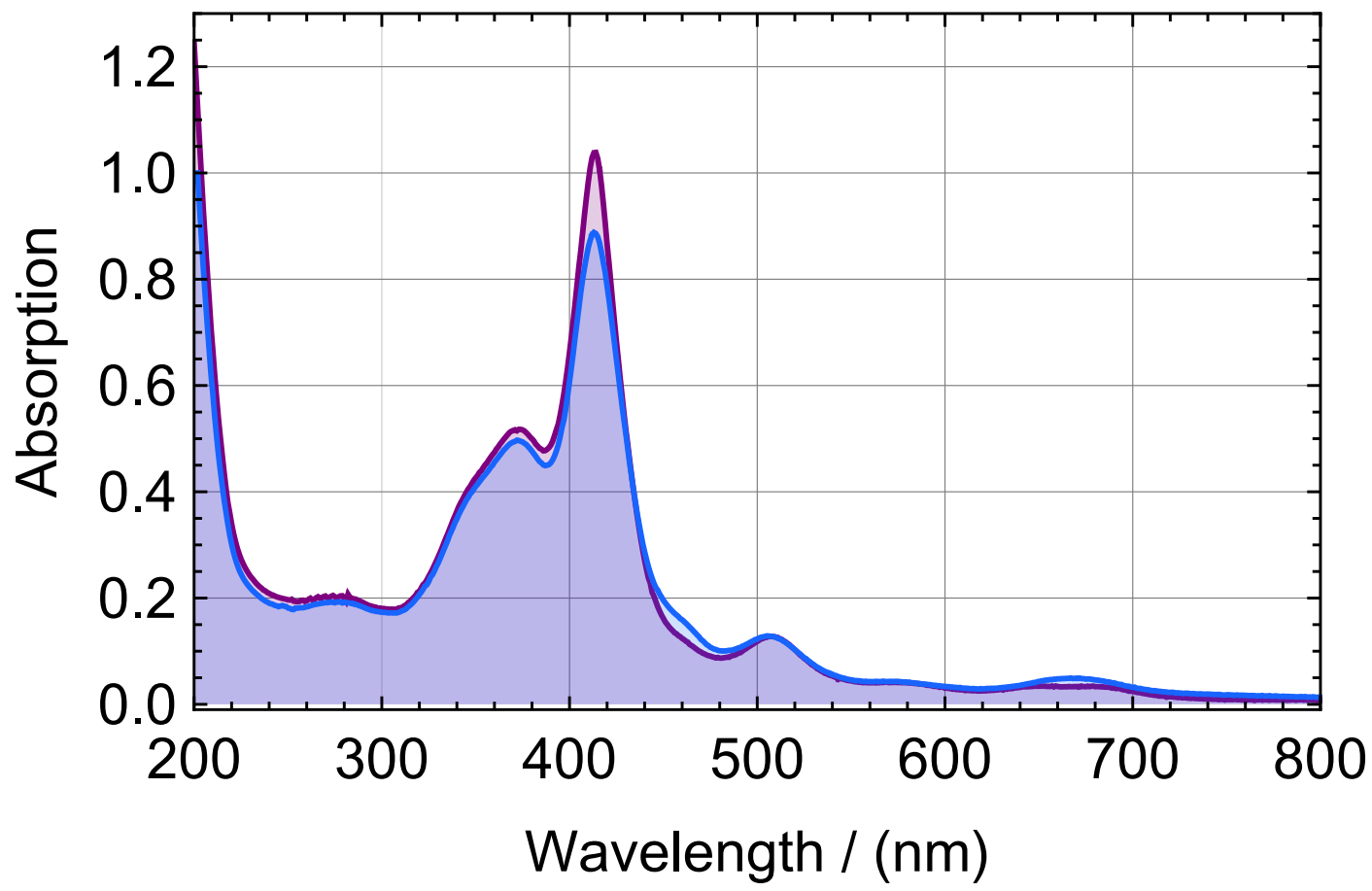


**Figure S25:** UV-vis absorption spectra of [(*p*–TMA)$Fe^{III}$–Cl] before (blue) and after (purple) irradiation with 390 nm light.

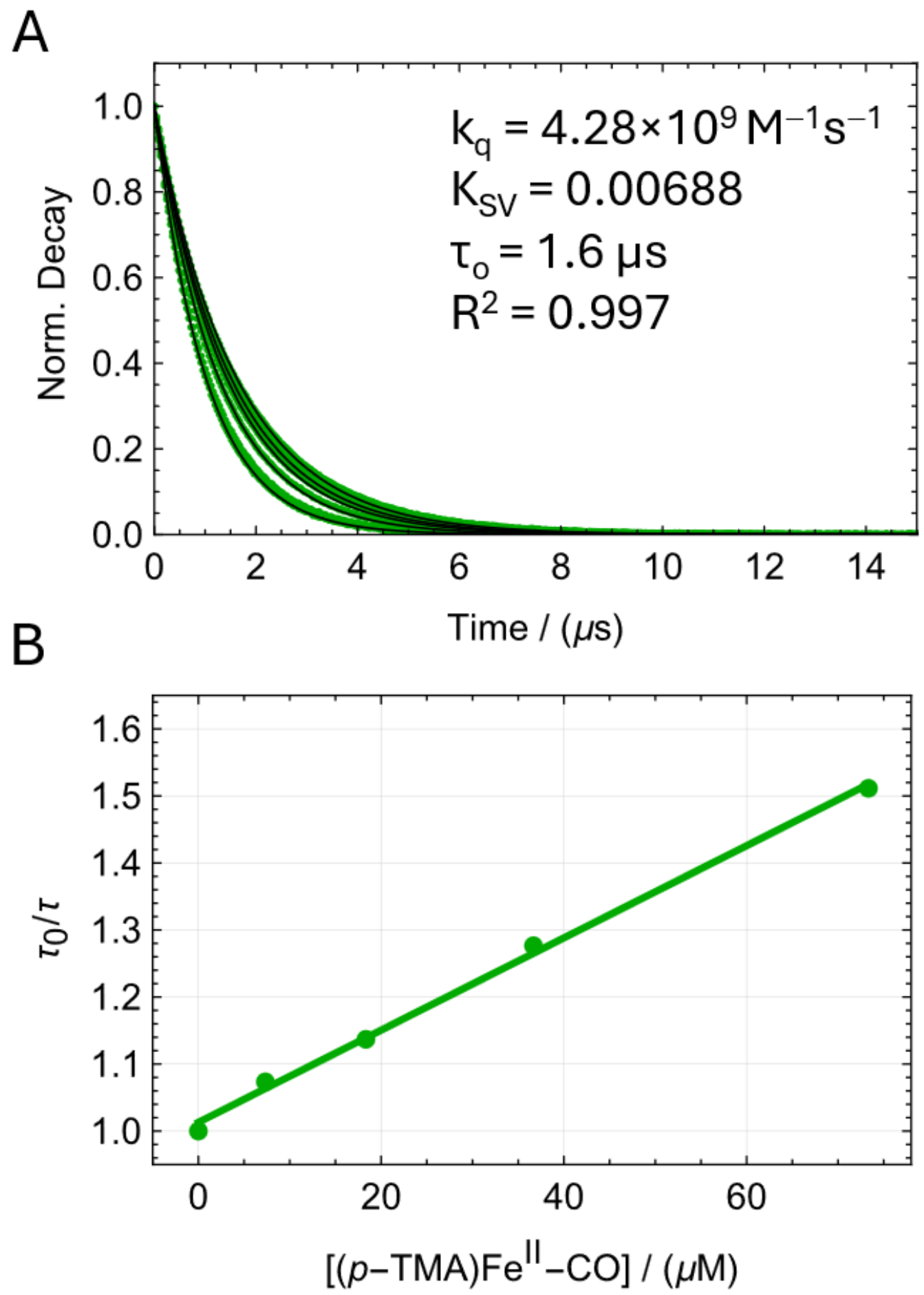


**Figure S26:** Time-resolved photoluminescent decay trace of **Ir1** with various concentration of [(*p*–TMA)$Fe^{II}$–CO] (**A**) and associated Stern-Volmer analysis (**B**). Fitting parameters for the Stern-Volmer plot are shown in (**A**).

# *Section 7: Cyclic Voltammetry of the $CO_2R$ catalyst*

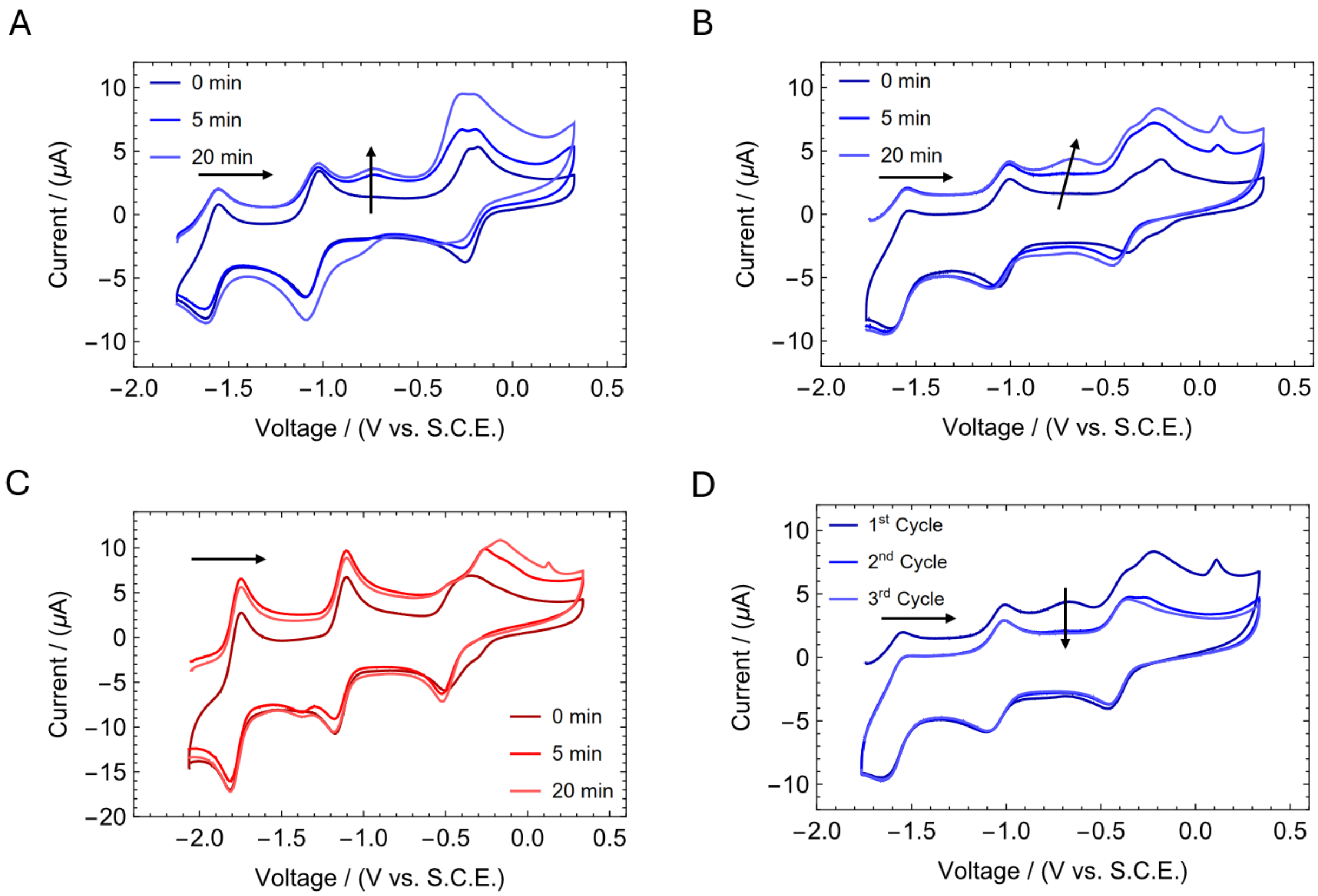


**Figure S27:** Cyclic voltammograms of (**A**) 0.5 mM [(*p*–TMA)$Fe^{III}$–Cl] in 0.1 M $TBAPF_6$/DMF, (**B**) 0.5 [(*p*–TMA)$Fe^{III}$–Cl] in 0.1 M $KPF_6$/DMF, and (**C**) 0.5 mM [(TPP)$Fe^{III}$–Cl] in 0.1 M $KPF_6$/DMF, recorded under Ar at a scan rate of 100 mV/s. CVs were recorded after holding the electrode potential at a value more negative than the Fe(I/0) couple for 0, 5, and 20 min. (**D**) Consecutive CV cycles of 0.5 mM [(*p*–TMA)$Fe^{III}$–Cl] in 0.1 M $KPF_6$/DMF under Ar at a scan rate of 100 mV/s, recorded after holding the electrode potential more negative than the $Fe^{I/0}$ couple for 20 min.

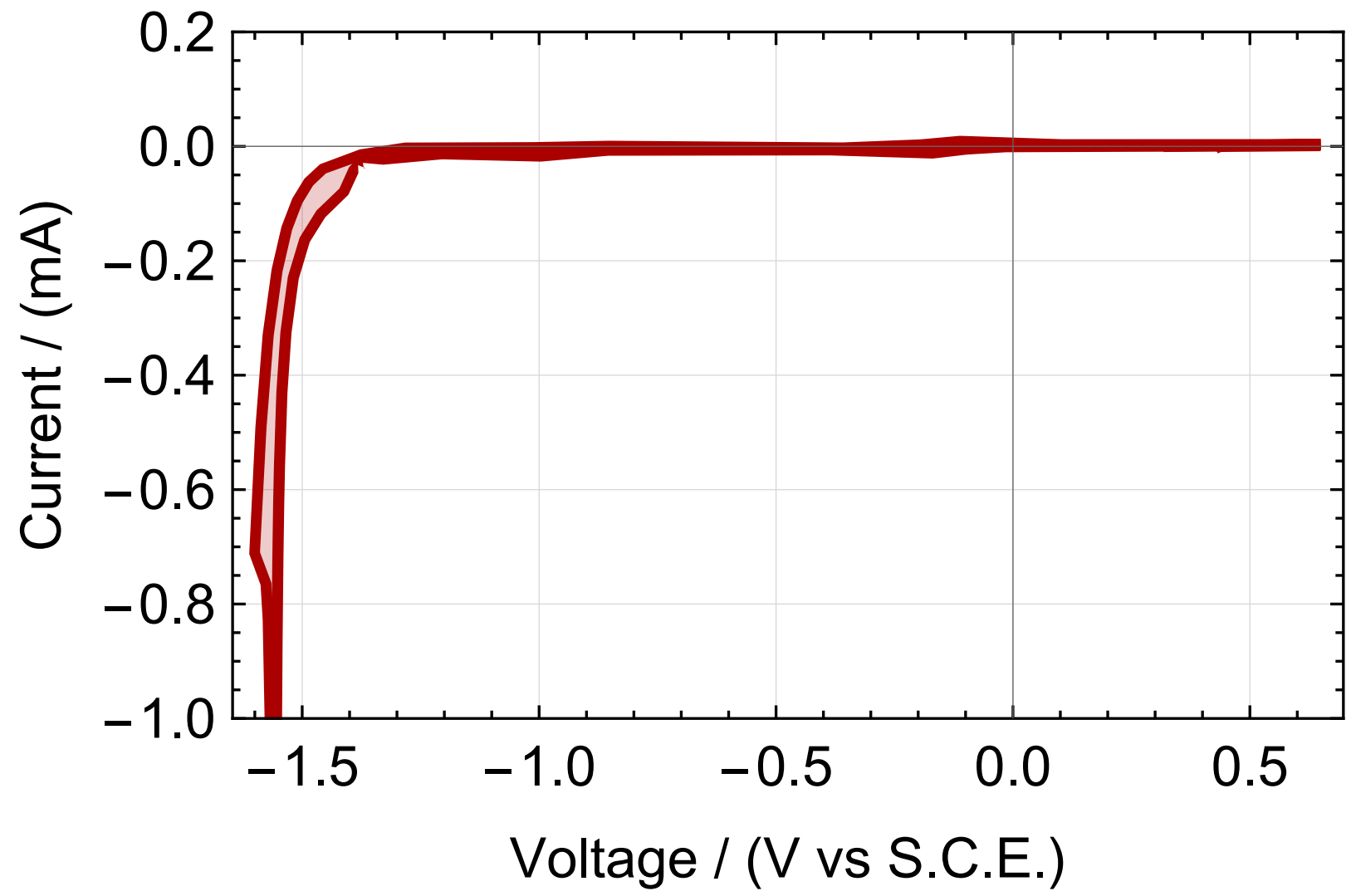


**Figure S28:** Cyclic voltammogram of [(*p*−TMA)$Fe^{III}$−Cl] (1 mM) in $CO_2$ sparged 30:1 vol:vol acetonitrile/$H_2O$ mixture. Water was used to solubilize [(*p*−TMA)$Fe^{III}$−Cl]. Scan rate: 100mV/s. Tetrabutylammonium hexafluorophosphate (100 mM) acted as the supporting electrolyte.

# *Section 8: Inner Filter Effects*

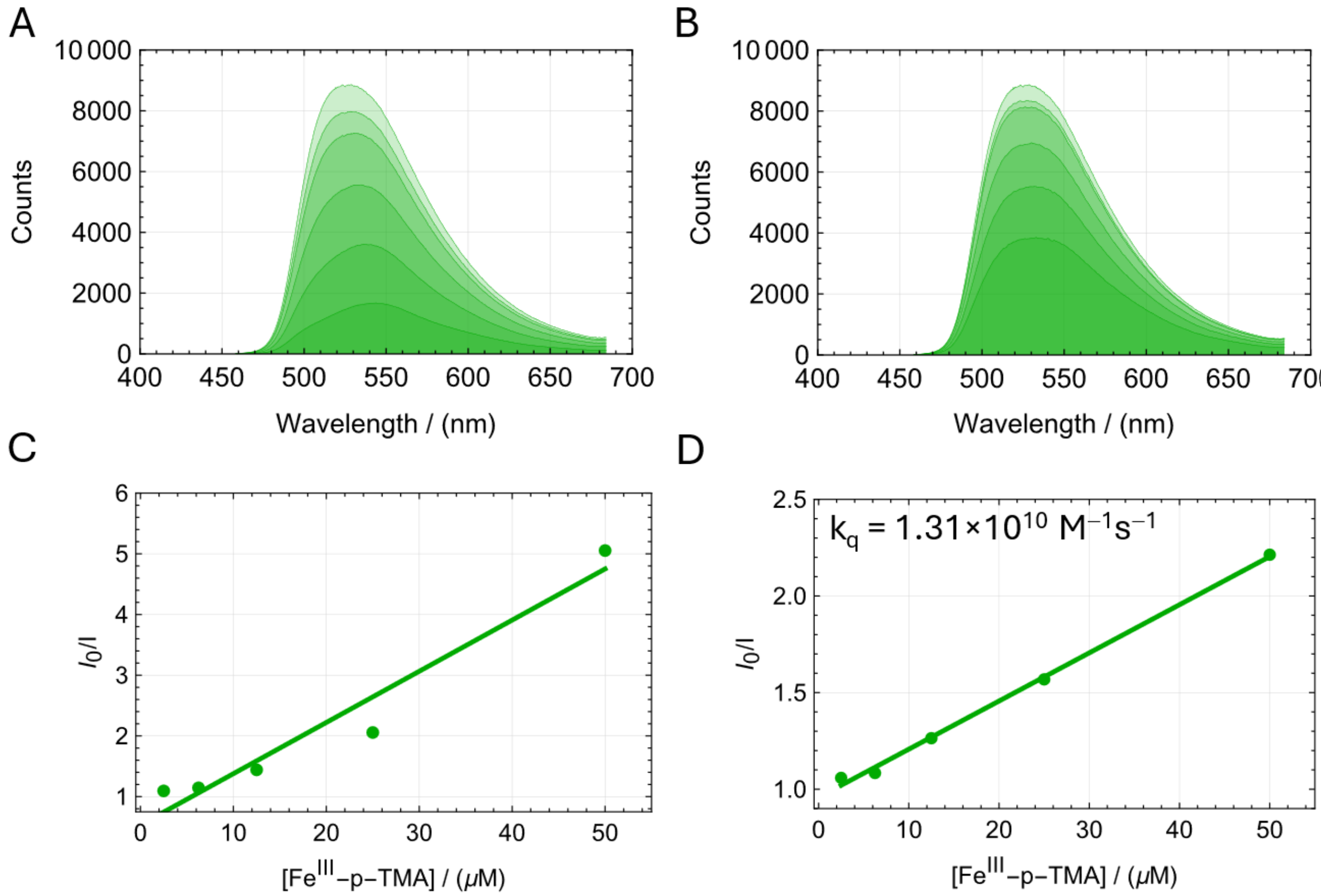


**Figure S29:** Steady-state photoluminescence of **Ir1** with various concentration of [(*p*–TMA)$Fe^{III}$–Cl] uncorrected (**A**) and corrected for primary and secondary inner filter effects (**B**). Associated Stern-Volmer analysis for the uncorrected (**C**) and corrected spectra (**D**).

[(*p*-TMA)$Fe^{III}$–Cl] absorbs strongly across the visible region of the electromagnetic spectrum, leading to possible primary or secondly inner filter effects that will attenuate the measured emission intensity irrespective of phenomena between the excited photosensitizer and [(*p*-TMA)$Fe^{III}$–Cl]. Primary effects include competitive absorption of the acceptor so that the steady-state concentration of PS* is lower, leading to diminished emission intensity. Secondary effects alternatively include absorption of emitted photons by the acceptor inside the cuvette. Consequently, the measured emission intensity as a function of concentration of quencher can be corrected if knowledge of absorbance at each concentration of quencher is known, in accordance with equation 6:

$$I_{corr} = I_{measured} * Log_{10}(OD_{exc} + OD_{em}(\lambda))/2) \qquad (6)$$

Raw photoluminescent spectra of **Ir1** at increasingly higher loadings of [(*p*-TMA)$Fe^{III}$–Cl] are shown in **Figure S29A** and clearly indicate a bathochromic shift in the measured $\lambda_{max}$ as [(*p*-TMA)$Fe^{III}$–Cl] increases, indicating secondary inner filter effects. The associated Stern-Volmer

plot using the steady-state photoluminescent spectra are not well fit by a linear equation (**Figure S29C**). Correction of the spectra in **Figure S29A** using equation 6 results in the spectra depicted in **Figure S29B**. The associated Stern-Volmer analysis using the integrated spectral profiles yields a plot well fit with a linear equation and an associated bimolecular quenching rate constant of $1.31\times10^{10}$ $M^{-1}s^{-1}$. This value slightly overestimates the measured value using time-resolved emission data ($8.07\times10^{9}$ $M^{-1}s^{-1}$) but is still in reasonable agreement.

# *Section 9: FRET Analysis*

Förster Resonance Energy Transfer (FRET) depends on numerous physical constants included in equation 7:

$$k(r)_{\mathrm{FRET}} = \frac{Q_D \kappa^2}{\tau_o r^6}\left(\frac{9000\, Ln(10)}{128\pi^5 N n^4}\right)\int_{\lambda}^{\lambda+d\lambda} F_D(\lambda)\varepsilon_A(\lambda)\lambda^4 d\lambda \qquad (7)$$

Where $F_D(\lambda)\varepsilon_A(\lambda)$ modulates the magnitude of $k(r)_{FRET}$ between specific excited-state energy donors and acceptors. These overlap integrals were calculated using a home-written Mathematica® script. First, the experimentally measured corrected photoluminescent spectra were area normalized to generate $F_D(\lambda)$. The product of the area normalized emission spectra at each wavelength with the respective molar absorptivity ($\varepsilon_A(\lambda)$) of [(*p*–TMA)$Fe^{III}$–Cl] or [(*p*–TMA)$Fe^{II}$–CO] was calculated and totaled to afford $\int_{\lambda}^{\lambda+d\lambda} F_D(\lambda)\varepsilon_A(\lambda)$. Plots of $F_D(\lambda)\varepsilon_A(\lambda)$ *vs.* λ for each PS with either [(*p*–TMA)$Fe^{III}$–Cl] or [(*p*–TMA)$Fe^{II}$–CO] are depicted in **Figure S30** and correlate well when considering the relative intensities of the absorption and emission spectra of the acceptors and donors respectively by inspection. For calculation of $J_{\mathrm{overlap}}$, nm was converted to wavenumber. When available, $k_p$ was taken from previously reported values. Otherwise. $k_p$ was measured using relative quantum yields with $[Ru(bpy)_3]^{2+}PF_6^-$ in acetonitrile (6.2%) as a standard. $\varepsilon_A(\lambda)$ for [(*p*–TMA)$Fe^{II}$–CO] was extracted from spectroelectrochemical data.

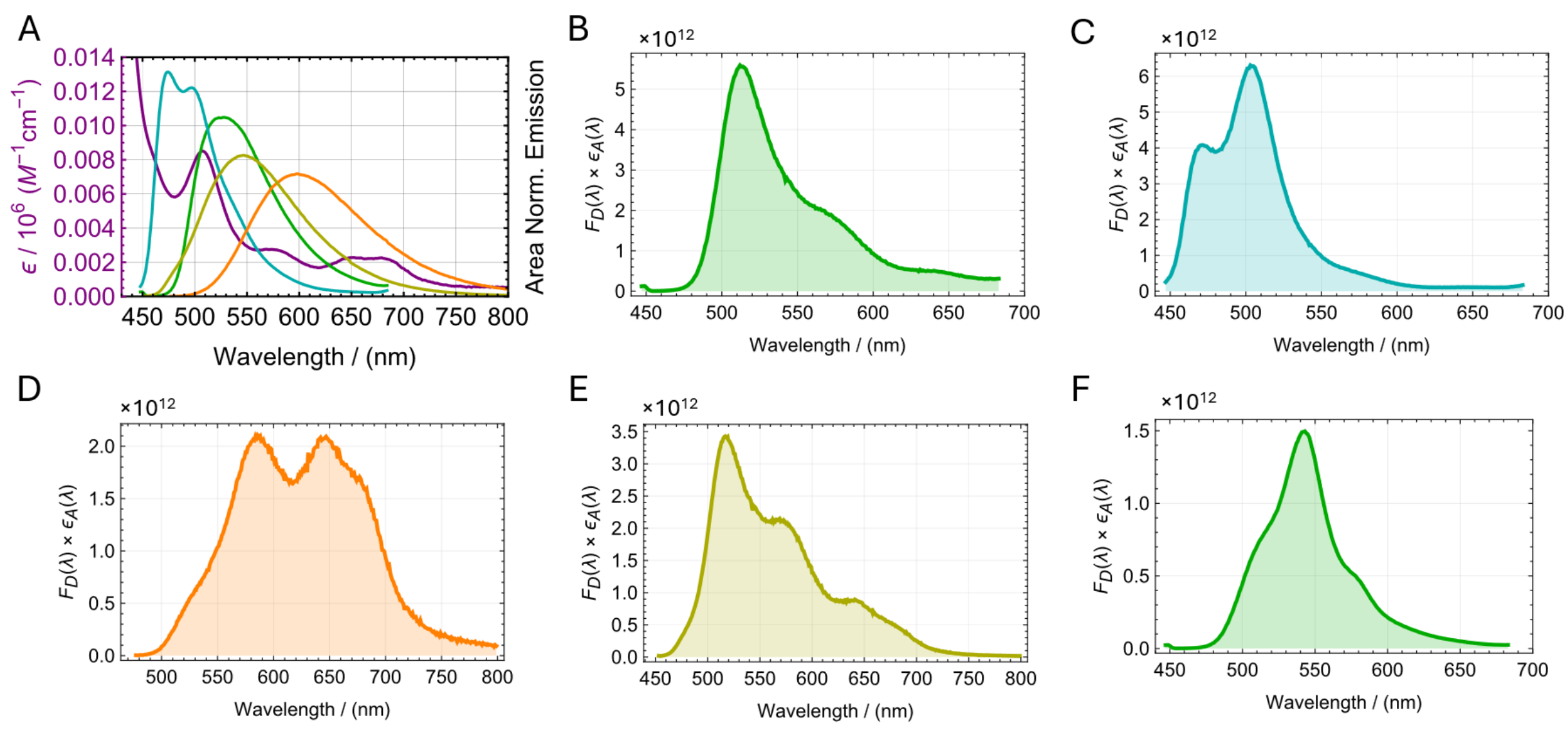


**Figure S30:** Absorption and emission spectra [(*p*–TMA)$Fe^{III}$–Cl] and **Ir1** (green), **Ir2** (teal), **Ir3** (orange), and **Ir4** (yellow) (**A**). Product spectra of $F_D(\lambda)$ and $\varepsilon_A(\lambda)$ for **Ir1** (**B**), **Ir2** (**C**), **Ir3** (**D**), and **Ir4** (**E**) and [(*p*–TMA)$Fe^{III}$–Cl]. (**F**) Product spectra of $F_D(\lambda)$ and $\varepsilon_A(\lambda)$ between **Ir1** and [(*p*–TMA)$Fe^{II}$–CO].

# *Section 11: Calibration Data*

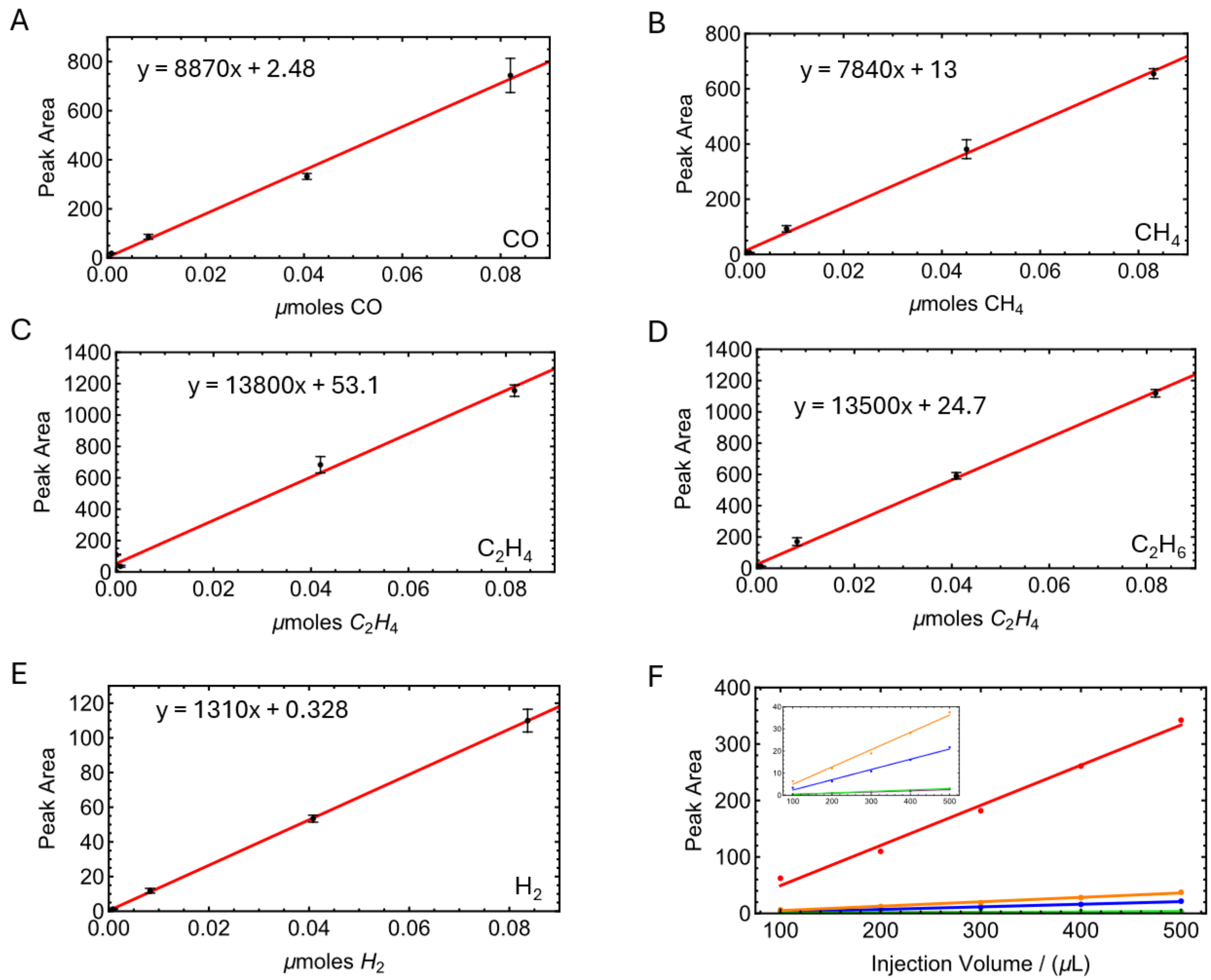


**Figure S31:** GC calibration data for CO (**A**), $CH_4$ (**B**), $C_2H_4$ (**C**), $C_2H_6$ (**D**), and $H_2$ (**E**). (**F**) Injection volume dependent responses for CO calibration gas.

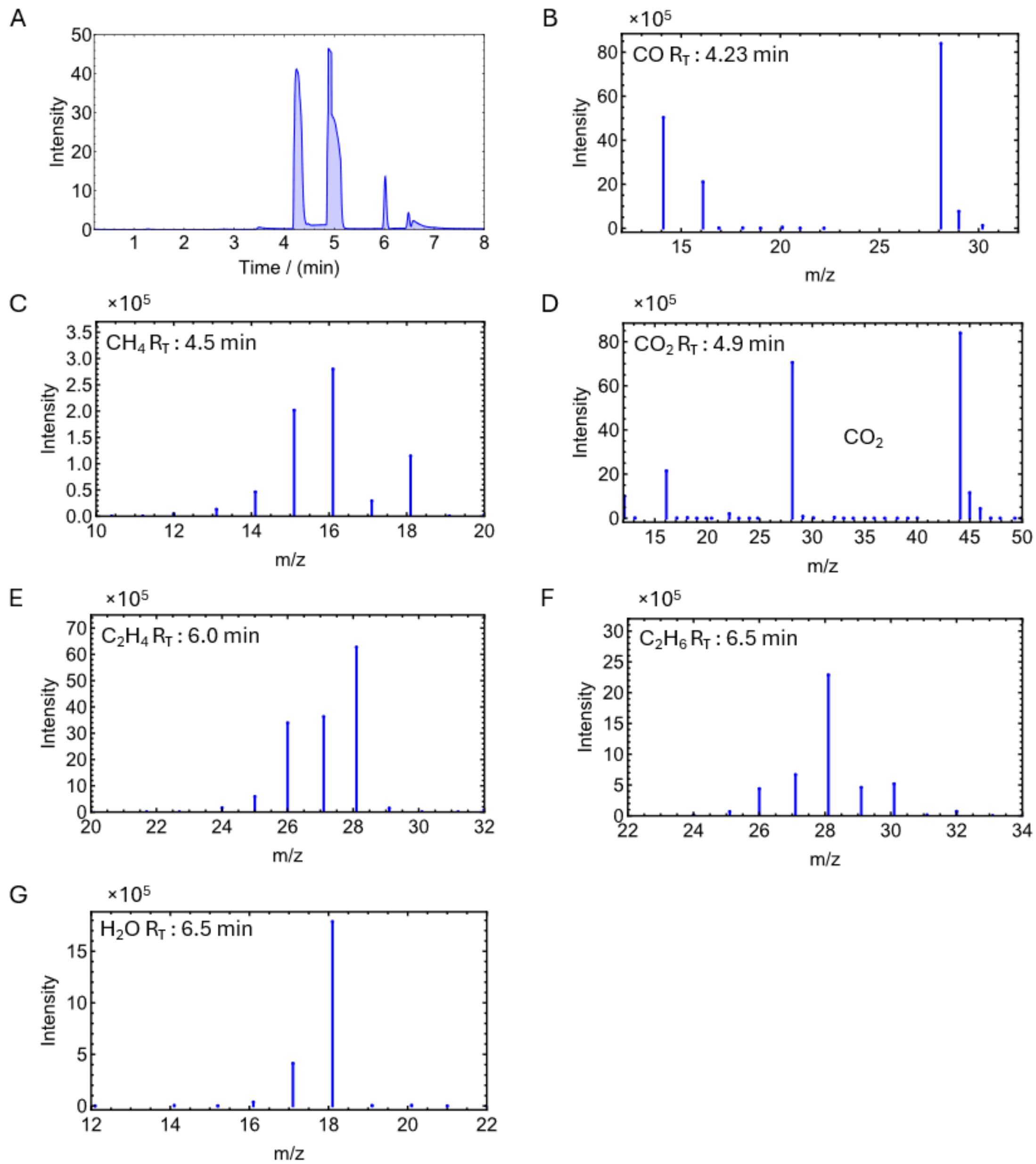


**Figure S32:** (**A**) Chromatogram from gas injections standards. Experimental splitting pattern for CO (**B**), $CH_4$ (**C**), $CO_2$ (**D**), $C_2H_4$ (**E**), $C_2H_6$ (**F**), and $H_2O$ (**G**). Retention times ($R_T$) are included in the inset for convenience.

# *Section 12: Synthesis and NMR Data*

**Synthesis of [Ir(2-phenylpyridine)$_2$(1,10-phenanthroline)]$^+$PF$_6^-$:** To a round bottom charged with a star bar was added 200mg of [Ir(Cl)(ppy)$_2$]$_2$, 74.0 mgs of phen (2.1 equivalents), and 15 mLs of ethylene glycol. The flask was then heated at 130 $^o$C for 16 hours in air. Upon cooling to room temperature, 25 mLs of a saturated $NaPF_{6(aq)}$ solution was added, which induced precipitation of a yellow solid and the sample was allowed to stir for 30 minutes to ensure ion exchange. The precipitate was collected via vacuum filtration and washed with 30 mLs of diethyl ether. The solid was redissolved in minimal acetonitrile and crystals were grown via the vapor diffusion method using acetonitrile/diethyl ether. $^1$H NMR (400 MHz, $CD_3CN$) δ 8.71 (d, *J* = 8.3, 1.4 Hz, 2H), 8.33 (d, *J* = 5.1, 1.4 Hz, 2H), 8.26 (s, 2H), 8.08 (d, *J* = 8.2 Hz, 2H), 7.92 – 7.83 (m, 4H), 7.81 (t, *J* = 7.8, 1.6 Hz, 2H), 7.48 – 7.40 (d, 2H), 7.11 (t, *J* = 7.6, 1.2 Hz, 2H), 6.99 (t, *J* = 7.5, 1.4 Hz, 2H), 6.88 (t, *J* = 7.4, 5.8, 1.4 Hz, 2H), 6.42 (t, *J* = 7.6, 1.2 Hz, 2H). $^{13}$C NMR (101 MHz, $CD_3CN$) δ 207.07, 168.03, 151.81, 150.38, 149.96, 147.40, 144.89, 139.04 (d, *J* = 6.0 Hz), 132.29, 132.17, 130.89, 128.83, 127.34, 125.42, 123.92, 123.18, 120.34.

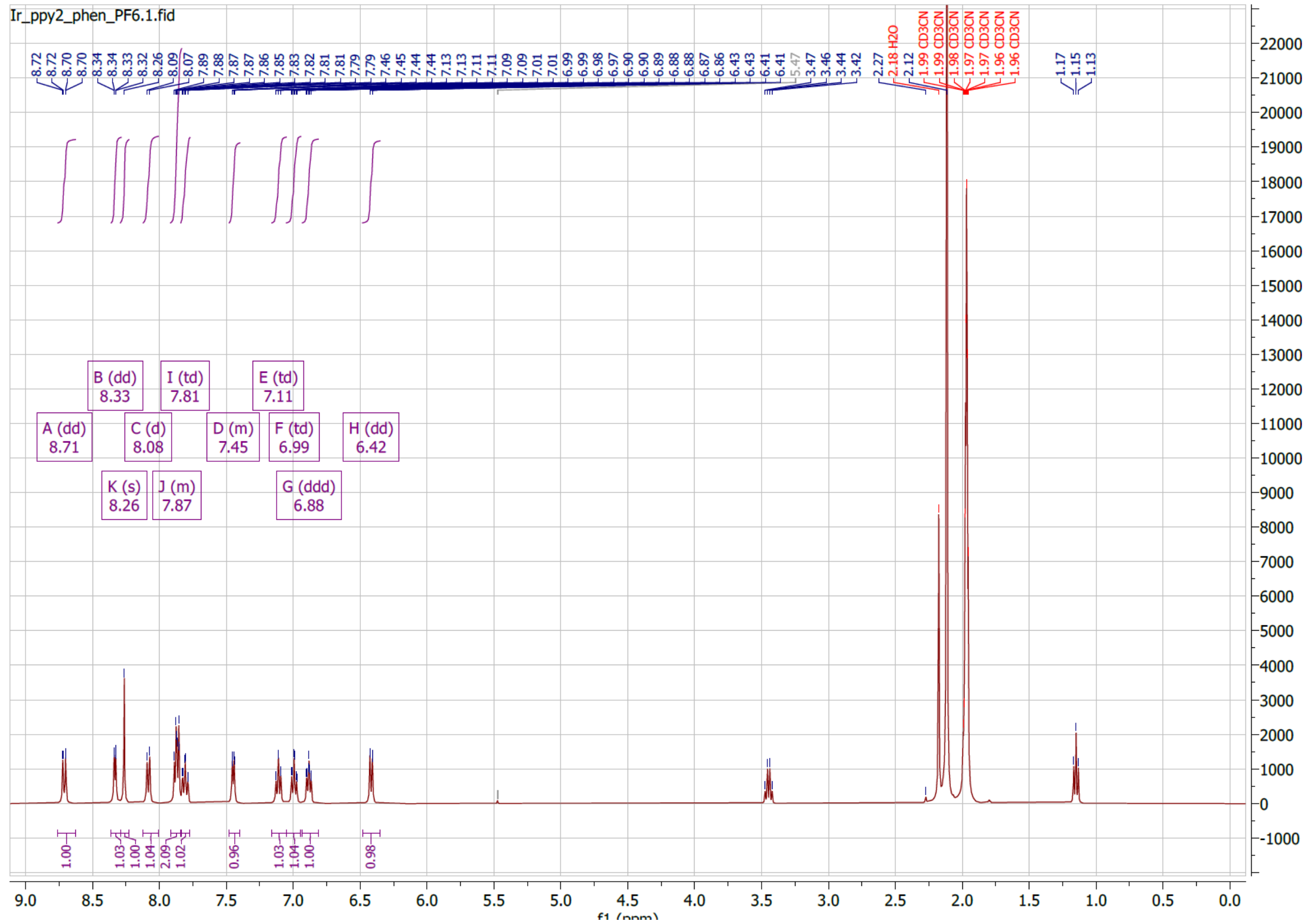


**Figure S33:** $^{1}H$ NMR spectra for **Ir3**.

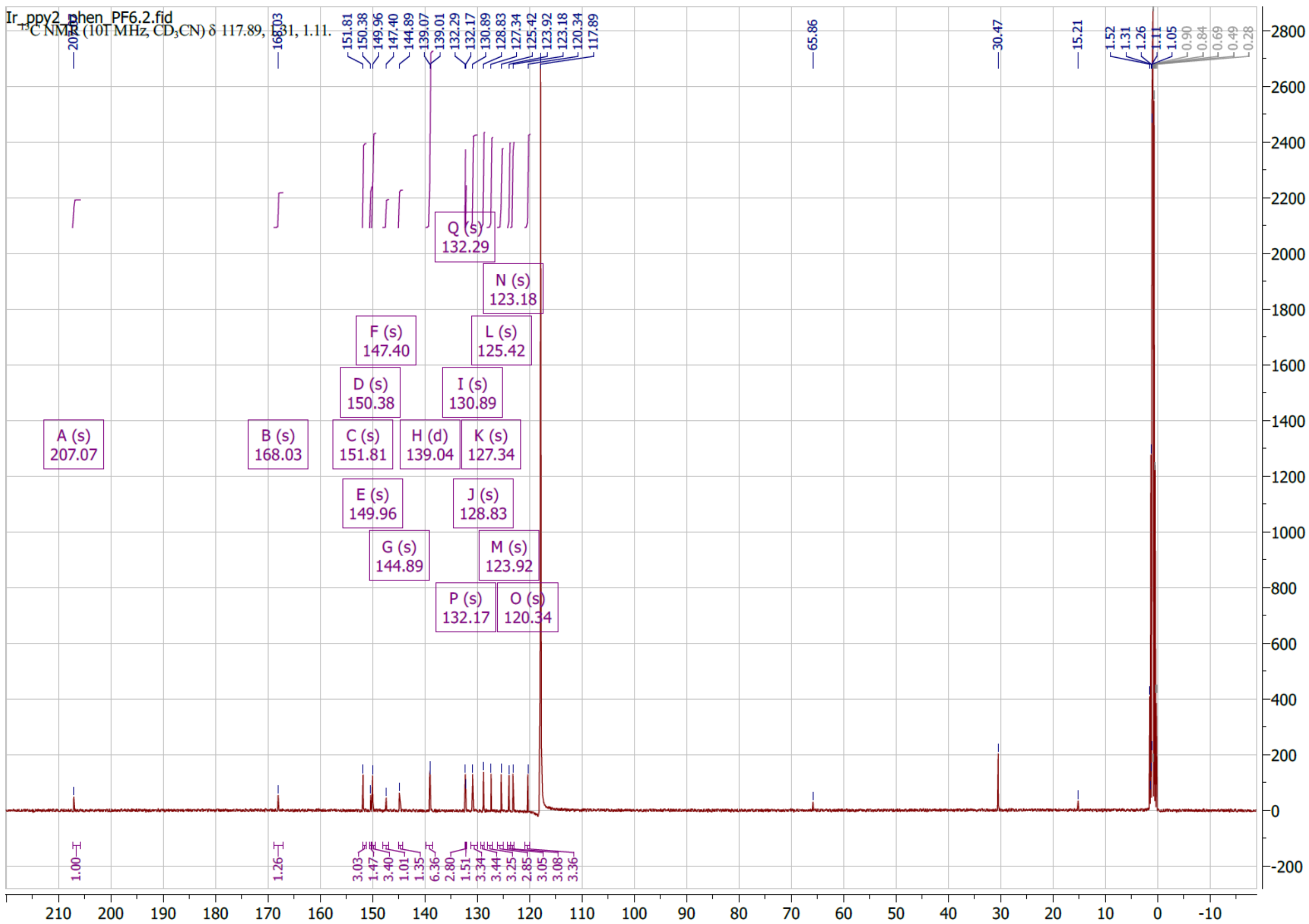

**Figure S34:** $^{13}$C NMR spectra for **Ir3**.

**Synthesis of [Ir(2-phenylpyridine)$_2$(3,4,7,8-Tetramethylphenanthroline)]$^+$PF$_6^-$:** To a round bottom charged with a star bar was added 100mg of [Ir(Cl)(ppy)$_2$]$_2$, 46.3 mgs of tetMphen (2.1 equivalents), and 10 mLs of ethylene glycol. The flask was then heated at 130 °C for 16 hours in air. Upon cooling to room temperature, 25 mLs of a saturated NaPF$_{6(aq)}$ solution was added, which induced precipitation of a yellow solid and the sample was allowed to stir for 30 minutes to ensure ion exchange. The precipitate was collected via vacuum filtration and washed with 30 mLs of diethyl ether. The solid was redissolved in minimal acetonitrile and crystals were grown via the vapor diffusion method using acetonitrile/diethyl ether. $^1$H NMR (400 MHz, CD$_3$CN) δ 8.39 (s, 2H), 8.07 (d, *J* = 8.2, 1.2 Hz, 2H), 8.01 (s, 2H), 7.85 (d, *J* = 7.7, 1.4 Hz, 2H), 7.80 (t, *J* = 8.2, 7.5, 1.6 Hz, 2H), 7.46 (d, *J* = 5.8, 1.6, 0.8 Hz, 2H), 7.10 (t, *J* = 7.5, 1.2 Hz, 2H), 6.98 (t, *J* = 7.4, 1.4 Hz, 2H), 6.87 (t, *J* = 7.4, 5.8, 1.4 Hz, 2H), 6.40 (d, 2H), 2.82 (s, 6H), 2.35 (s, 6H). $^{13}$C NMR (101 MHz, CD$_3$CN) δ 168.24, 151.99, 151.32, 149.73, 146.95, 145.93, 144.85, 138.81, 135.84, 132.25, 130.83, 130.49, 125.40, 124.86, 123.69, 122.93, 120.30, 17.69, 14.86.

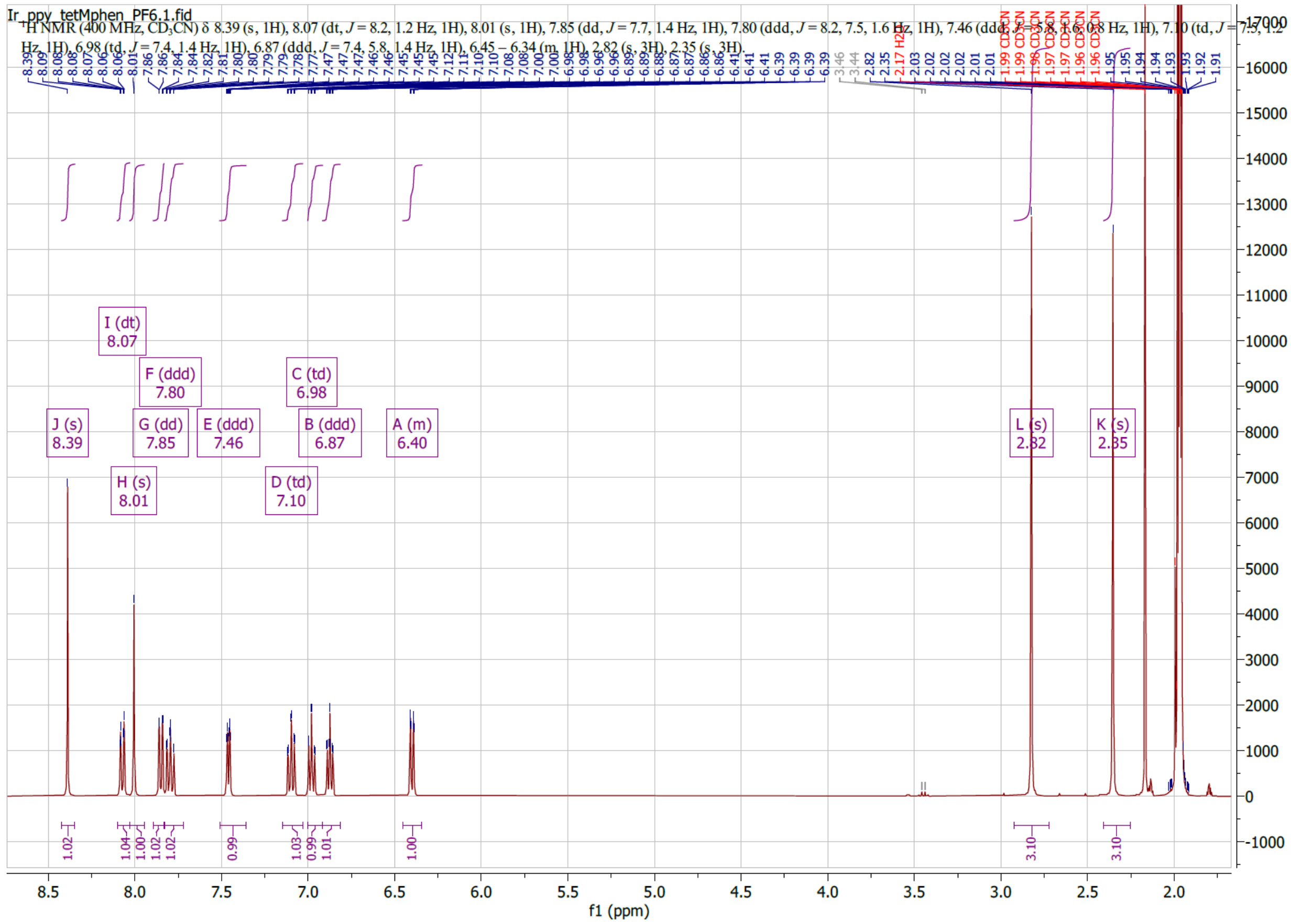

**Figure S35:** $^1H$ NMR spectra for **Ir4**.

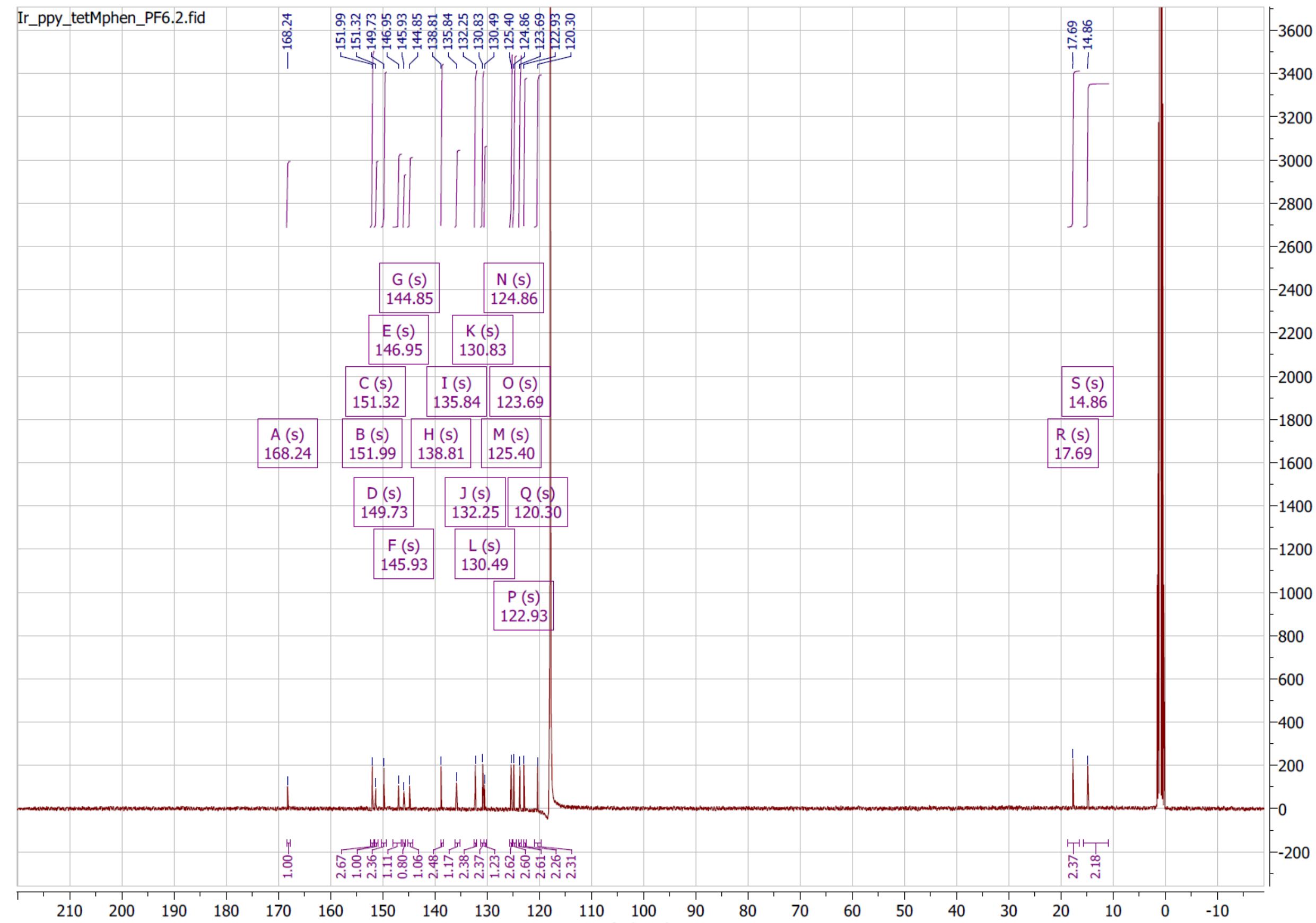


**Figure S36:** $^{13}$C NMR spectra for **Ir4**.

**Synthesis of Tetrabutylammonium$^{+}$ Oxalate$^{2-}$:** $(TBA)_2Ox^{2-}$ was synthesized in accordance with a previous report. $^{1}H$ NMR and $^{13}C$ NMR spectra are in agreement with those previously measured.[8] Note: $(TBA)_2Ox^{2-}$ is an incredibly hydroscopic solid. It was consequently stored under an argon atmosphere.

**Synthesis of [(*p*−TMA)Fe$^{III}$−Cl]:** [(*p*−TMA)Fe$^{III}$−Cl] was synthesized in accordance with a previous report.[9]

**Photochemical synthesis of [(*p*−TMA)$Fe^{II}$−CO]:** A freshly prepared 120 μM stock solution of [(*p*−TMA)$Fe^{III}$−Cl] in acetonitrile was transferred to a 1 cm quartz cuvette equipped with a PTFE screw cap vial. The solution was first sparged with Argon for 5 minutes, followed by CO for 10 minutes. The cap was covered with electrical tape and the sample was irradiated using a 370 nm Kessil LED operating at 25% power efficiency. The cuvette was placed 5 cm away from the Kessil LED. Irradiation ceased when no further changes in the UV-vis spectrum were observed.

**References:**


[1] Sattler, W.; Ener, M. E.; Blakemore, J. D.; Rachford, A. A.; LaBeaume, P. J.; Thackeray, J. W.; Cameron, J. F.; Winkler, J. R.; Gary, H. B. Generation of Powerful Tungsten Reductants by Visible Light Excitation. *J. Am. Chem. Soc*. **2013**, *135*, 10614–10617.

[2] DiLuzio, S.; Valloli, L. K.; Kudisch, M.; Chambers, D. T.; Rumbles, G.; Reid, O. G.; Bird, M. J.; Sayre, H. J. Reconceptualizing the $Ir^{III}$ Role in Metallaphotoredox Catalysis: From Strong Photooxidant to Potent Energy Donor. *ACS Catal*. **2024**, *14* (15), 11378–11388.

[3] Cusumano, A. Q.; Chaffin, B. C.; Cagan, D. A.; DiLuzio, S. Sutcliffe, E.; Hadt, R. G.; Doyle, A. G. Light-Promoted C($sp^3$)–C($sp^3$) Reductive Elimination from Dialkyl $Ni^{II}$ Complexes. *J. Am. Chem. Soc*. **2025**, 147, 32941–32950.

[4] Cagan, D. A.; Bím, D.; Silva, B.; Kazmierczak, N. P.; McNicholas, B. J.; Hadt, R. G. Elucidating the Mechanism of Excited-State Bond Homolysis in Nickel–Bipyridine Photoredox Catalyst. *J. Am. Chem. Soc*. **2022**, *144*, 6516–6531.

[5] Monti, F.; Baschieri, A.; Sambri, L.; Armaroli, N. Excited-State Engineering in Heteroleptic Ionic Iridium(III) Complexes. *Acc. Chem. Res*. **2021**, 54, 1492–1505.

[6] Lowry, M. S.; Goldsmith, J. I.; Slinker, J. D.; Rohl, R.; Pascal, R. A.; Malliaras, G. G.; Bernhard, S. Single-Layer Electroluminescent Devices and Photoinduced Hydrogen Production from an Ionic Irirdium(III) Complex. *Chem. Mater*. **2005**, *17* (23), 5712–5719.

[7] Draper, F.; DiLuzio, S.; Sayre, H. J.; Pham, L. N.; Coote, M. L.; Doeven, E. H.; Francis, P. S.; Connell, T. U. Maximizing Photon-to-Electron Conversion for Atom Efficient Photoredox Catalysis. *J. Am. Chem. Soc*. **2024**, *146* (39), 26830–26843.

[8] Beattie, J. W.; White, D. S.; Bheemaraju, A.; Martin, P. D.; Gorysman, S. Recyclable chemosensor for oxalate based on bimetallic complexes of a dinucleating bis(iminopyridine) ligand. *Dalton Trans.*, **2014**, *43*, 7979–7986.

[9] Costentin, C.; Robert, M.; Saveant, J.; Tatin, A.; Azcarate, I. Selective Porphyrin-Catalyzed Electrochemical Reduction of $CO_2$ into CO in Water. 20180023204, January 25, 2018.
